# Stability Analysis and Heterotic Studies in Maize (*Zea mays* L.) Inbreds to Develop Hybrids With Low Phytic Acid and High-Quality Protein

**DOI:** 10.3389/fpls.2021.781469

**Published:** 2022-01-19

**Authors:** J. Lydia Pramitha, John Joel, Ravikesavan Rajasekaran, D. Uma, Kumari Vinothana, Meenakumari Balakrishnan, K. R. V. Sathyasheela, Raveendran Muthurajan, Firoz Hossain

**Affiliations:** ^1^Tamil Nadu Agricultural University, Coimbatore, India; ^2^School of Agriculture and Biosciences, Karunya Institute of Technology and Sciences, Coimbatore, India; ^3^Division of Genetics, Indian Agricultural Research Institute, New Delhi, India

**Keywords:** lpa (low phytic acid), tryptophan, combining ability, AMMI, GGE biplot, heterotic grouping

## Abstract

Maize is a major staple crop with high value as food and feed in the poultry sector. Considering the overall nutritional value, maize-based diets comprise two major constraints, i.e., higher phytic acid (PA) and lack of tryptophan. To overcome these issues, a set of identified stable donors for low PA (*lpa*) and higher tryptophan were crossed in a line × tester fashion, and the hybrids obtained were evaluated at three locations with two replications. Among the inbreds for yield, UMI 1201 and UMI 1205 were the stable good combiners, and for PA, UMI 447 and LPA-2-285 were identified as efficient combiners across locations. Subsequently, 72 hybrids developed from these inbreds had a reduced phytate and higher tryptophan compared with checks having alterations in their yield levels. From Additive Main Effects and Multiplicative Interaction (AMMI) and Genotype main effect plus genotype-by-environment interaction (GGE) biplots, DMR-QPM-09-13-1 × UMI 1099 (PA:9.38 mg/g, trp:0.06%, and yield:184.35 g) and UMI 1205 × UMI 467 (PA:7.04 mg/g, trp:0.06%, and yield:166.39 g) were stable for their high yield with medium PA and tryptophan. Also, across environments, UMI 1200 × UMI 467 had a stable average yield of 129.91 g along with the lowest PA of 4.50 mg/g and higher tryptophan of 0.07%. Thus, these hybrids could be selected and evaluated in upcoming biofortification trials to benefit the poultry sector. Furthermore, the parental inbreds utilized were grouped into heterotic pools to serve as a source population for the development of *lpa* hybrids in future programs.

## Introduction

Maize is a C_4_ cereal that serves as a major staple food and feeds for poultry as well as swine due to its easier availability, palatability, and nutritional emolument (Shah Rouf et al., 2016). It is rich in carbohydrates, vitamins, minerals, proteins, and trace elements such as selenium and magnesium (Farnham et al., 2003). Although it has a higher nutritional value, the presence of phytic acid (PA) and the lack of tryptophan in its zein have been an issue in ascertaining the bioavailability of nutrients and proteins in maize diets (Naidoo et al., 2012). The higher PA in maize hinders nutrient absorption in monogastric animals due to its polyanionic nature and causes the chelation of positively charged mineral cations such as iron and zinc (Raboy et al., 2001). Unlike plants, the nonruminants lack the enzyme phytase for dissolution, and this PA complex from maize feeds thus obstructs mineral absorption.

An alternative approach to resolve this is by supplementing artificial fungal phytase in poultry feeds increased the feed cost rather than enhancing the micronutrient bioavailability (Lelis et al., 2012). Hence, developing low PA (*lpa*) lines would offer a possible solution to mineral absorption in maize-based foods. Speaking of nutrition, another subsequent lacuna in maize zein is the absence of tryptophan, and it is an essential amino acid with numerous regulatory functions (Vasal, 2000). The solution to this was effected by incorporating *opaque-2* with its genetic modifiers in the endosperm (Ignjatovic-Micic et al., 2014). However, this remains incomplete without manipulating the PA in maize for efficient absorption of minerals to achieve overall nutritional value. Hence, combining *lpa* and higher tryptophan in breeding programs will overcome the prime nutritional lacuna faced in maize-based foods. Furthermore, these improved hybrids would eventually increase the body mass ratio and nutrient uptake in chicks with a simultaneous cutdown of additional chemical supplements in diets (Lelis et al., 2012).

In addition, these nutritional improvisations should also be accompanied by increased yield for their commercialization, and this reinstates the utilization of heterosis in maize by hybridizing the identified donors with elite lines (Sureshkumar et al., 2014). Hybrid development for cultivation necessitates its stable performance as PA and tryptophan in maize are reported to vary across locations (Zaidi et al., 2008; Brankovic et al., 2016). Hence, identifying stable hybrids with uniform genotype and environment (G × E) interactions by effective models such as Additive Main Effects and Multiplicative Interaction (AMMI) and Genotype main effect plus genotype-by-environment interaction (GGE) biplot enhances the selection for these traits in breeding trials. These numerical and graphical approaches in stability are predominantly used to discriminate the genotypes for response variable and mega environments graphically thereby locating stable lines in crop improvement programs (Akhtar et al., 2018; Vaezi et al., 2019).

Speaking of both *lpa* and tryptophan in a hybrid, this is an initial program aimed to produce a high-yielding stable maize hybrid with a desirable lower PA and higher tryptophan content. Therefore, this could be used as a base to formulate the upcoming integrated breeding approaches in maize. In this study, a set of 17 inbreds identified as potential donors for *lpa* and higher tryptophan from our previous findings (Pramitha et al., 2020b) were hybridized in a line × tester mating design, and the resultant hybrids were raised across diverse locations for analyzing its stability. To further dissect its genetic and molecular backgrounds, these stable parental inbreds have been grouped in heterotic pools to facilitate the production of high-performing hybrid combinations with superior nutritional parameters in near future.

## Materials and Methods

The stable inbreds were selected from the framed germplasm reference set in maize (Pramitha et al., 2020a). This included eight inbreds for *lpa*, six for higher tryptophan, and three for yield. They were obtained from the Department of Millets, TNAU, and were intercrossed in a line × tester fashion. The six inbreds for tryptophan and three high-yielding lines were used as females, with eight *lpa* inbreds as male (Table 1). The resultant 72 hybrids with two agronomically superior checks, namely, CO 6 and COH (M) 8, were raised at three locations, namely, E_1_: Coimbatore, E_2_: Vagarai, and E_3_: Bhavanisagar (Table 2). The parents and the hybrids were evaluated in a randomized block design with two replications in each location (Pramitha et al., 2020b).

**Table 1:** The parental inbreds (female lines and male testers) used in the study.

**S.No**	**Lines**	**Code**	**Days to maturity**	**S.No**	**Testers (*lpa* inbreds)**	**Code**	**Days to maturity**
1.	UMI 1200	L1	117.00	1.	LPA-2-285	T1	111.67
2.	UMI 1201	L2	119.83	2.	LPA-2-395	T2	118.50
3.	UMI 1205	L3	117.83	3.	UMI 447	T3	119.50
4.	DMR-QPM-01-06-02	L4	117.00	4.	UMI 467	T4	118.00
5.	DMR-QPM-03-72	L5	115.50	5.	UMI 158	T5	115.17
6.	DMR-QPM-04-05	L6	116.67	6.	UMI 1099	T6	119.67
7.	DMR-QPM-06-12	L7	114.50	7.	K-155	T7	115.33
8.	DMR-QPM-09-13-1	L8	117.67	8.	UMI 113	T8	117.67
9.	DMR-QPM-11-17	L9	114.67				

**Table 2:** Description of the three locations in stability.

**S.No**	**Location**	**Latitude**	**Longitude**	**Soil type**	**Annual rainfall**
1.	Coimbatore	11.00° N	76.98° N	Red loamy	827 mm
2.	Bhavanisagar	11.46° N	77.10° N	Sandy loam soil	717 mm
3.	Vagarai	10.58° N	77.56° N	Black soil	600 mm

Both hybrids and parental lines were screened for 20 morphological traits, namely, days to 50% tasseling, days to 50% silking, anthesis silking interval, plant height, tassel length, number of tassel branches, cob placement height, cob length, cob girth, number of rows per cob, number of kernels per row, cob weight, shank weight, shelling percentage, 100 seed weight, seed length, seed girth, seed thickness, single plant yield, and seedling vigor index (Abdul-Baki and Anderson, 1973); and for four biochemical traits, namely, PA (Davies and Reid, 1979), inorganic phosphorus (Raboy, 2000), starch (Clegg, 1956), and tryptophan (Galicia et al., 2009). Finally, the iron and zinc of the selected stable hybrids with checks were also analyzed (Palmer and Piper, 1966).

### Biochemical Estimation

The standard protocols for the five nutritional traits are furnished below.

#### Phytic Acid

A finely ground whole grain maize sample (0.5 g) was taken from each genotype and continuously stirred for 3 h with 10 ml of 0.5 M HNO_3_. The sample was then filtered *via* Whatman No. 1 paper, and 200 μl of the aliquot was transferred to Eppendorf tubes having 200 μl of freshly prepared ferrous ammonium sulfate (2.16 mg/ml). These tubes were shaken and incubated in a boiling water bath for 20 min. Following this, the tubes were cooled, and 1 ml of isoamyl alcohol and 20 μl of ammonium thiocyanate (5 g/50 ml) were added. The tubes were shaken well and then centrifuged at 3,000 rpm for 10 min. The color developed was read at 460 nm, and it was spectrophotometrically analyzed in two replicates with sodium phytate as a standard (Davies and Reid, 1979).

#### High Inorganic Phosphorous (HIP) Assay

The grounded whole maize kernel samples of 0.1 g were soaked overnight in 0.4 M HCl. The next day after shaking, 100 μl of the extract was separately taken in another tube, and 900 μl of freshly prepared Chen's reagent (6N H_2_SO_4_:2.5% ammonium molybdate:10% ascorbic acid:H_2_O [1:1:1:2, v/v/v/v]) was added. The developed phosphomolybdate complex after an incubation of 30 min was read at 660 nm in a spectrophotometer with two replications having serial dilutions of potassium dihydrogen phosphate as a standard (Raboy, 2000).

#### Starch

Two grams of powdered whole grain samples were homogenized with 80% ethanol and centrifuged at 12,000 rpm for 15 min. This centrifugation was repeated until the washings of the supernatant with anthrone were colorless. When a colorless washing with anthrone was obtained, the supernatant was discarded, and the residue was well dried in a hot water bath. After drying, 5 ml of water and 6.5 ml of 52% perchloric acid were added and incubated for 20 min at 0°C. The supernatant was then transferred to a volumetric flask, and again, the extraction with 52% perchloric acid and water was repeated. This second supernatant was also added to the same volumetric flask, and the volume was made up to 100 ml with water. Notably, 0.1 ml of the pooled extracts were taken and made up to 1 ml with water. To this, 4 ml of anthrone (200 mg in 5 ml distilled ethanol made up to 100 ml with ice-cold 95% sulfuric acid) was added and kept in a hot water bath. The color developed was read at 630 nm in two replications in a spectrophotometer, and glucose was used as a standard (Clegg, 1956).

#### Tryptophan

Two grams of powdered whole grain samples were placed in a filter paper envelope and were defatted in a soxhlet apparatus with hexane for 3 h. Notably, 80 mg of the defatted powder was added in a falcon tube with 3 ml of papain solution 40 mg of papain dissolved in 0.165 N of sodium acetate (pH:7.5). These tubes were vortexed and kept in a hot air oven at 64°C for 16 h. After cooling, the tubes were centrifuged at 3,600 rpm for 5 min, and 1 ml of hydrolysate was added to another tube containing 3 ml of freshly prepared reagent D (20 ml of 1.8 mM ferric chloride prepared in 100 ml of glyoxylic acid stock (0.9025 g of glyoxylic acid in 100 ml conical flask is added with 50 ml of 7N H_2_SO_4_ and made up to 100 ml) + 20 ml of 30 N H_2_SO_4_) was vortexed. This was further incubated at 64°C for 30 min, and the tubes after cooling were read at 560 nm in two replications with a series of tryptophan (Galicia et al., 2009) as standards in the spectrophotometer.

#### Iron and Zinc

To 0.5 g of whole grain maize samples, 10 ml of a triacid mixture (9 ml nitric acid:2 ml sulfuric acid:1 ml perchloric acid) was added and allowed for digestion at room temperature overnight. On the following day, another digestion of these samples was carried out in a hot sand bath until the digest was clear and colorless. After cooling, the contents were diluted to 50 ml with double distilled water, and the Fe and Zn (Palmer and Piper, 1966) were read at 248.33 and 213.86 nm, respectively, in an atomic absorption spectrophotometer.

### Statistical Analysis

The combining ability analysis (Kempthorne, 1957) and the standard heterosis (Meredith and Bridge, 1972) with a high yielding check CO 6 were estimated in the statistical package of TNAUSTAT. The pooled ANOVA, including AMMI and GGE biplot for stability given by Gauch (1992), was analyzed in R studio 3.6.2 and PBTools 1.4 (Pramitha et al., 2020b). The classification of inbreds based on their combining ability in heterotic pools was carried out based on the diverse general combining ability (*gca*) effects adopted by Legesse et al. (2009).

## Results and Discussion

The main focus of this study was to overcome the major nutritional stern in maize, and it was successful with the efficient donors for *lpa* and tryptophan (Pramitha et al., 2020b). This is the initial research framed to combine these two traits in hybridization, and the observations of this study could be used in other integrated programs of maize. Maize is well known for its heterotic expression from the eras of Shull (1909), and in this, the expression of a phenotype across locations has revealed the importance of attributing traits beneath a response factor. The phenotypic expression of a genotype depends on the G × E interaction. In addition, several contributing traits and genes over locations here have influenced the ability to transfer the favorable alleles in a cross for complex characters (Ferreira et al., 2018; Murtadha et al., 2018).

Due to this, the *per se* and combining ability of a parent in a crossing program is important, and it confers the ability to allocate favorable genes in a hybrid (Fasahat, 2016). Thus, this study across locations enables us to understand the variations in mean, combining ability effects and heterotic potential among the parental inbreds and hybrids for yield, PA, and tryptophan. The ANOVA for pooled combining ability analysis revealed a significant variation for lines and testers (Table 3). Accordingly, these varying genotypes and their cross combinations were evaluated at three locations to identify stable parents and hybrids for improved yield, tryptophan, and *lpa* (Lahane et al., 2014; Kumar et al., 2016). The selected desirable hybrids from each location were finally subjected to stability analysis to identify an ideal hybrid for further evaluation.

**Table 3:** ANOVA for combining ability across locations.

**SOURCE**	**REP**	**CROSS**	**LINE**	**TESTER**	**LINE × TESTER**	**ERROR**
**df**	**1**	**71**	**8**	**7**	**56**	**71**
50 DT^*^	0.174	10.102^**^	21.804^**^	13.213^**^	8.042^**^	0.089
50 DS	0.037	8.740^**^	17.542^**^	13.517^**^	6.885^**^	0.110
ASI	0.050	0.874^**^	1.460^**^	0.441^**^	0.845^**^	0.059
CPH	0.293	196.478^**^	296.569^**^	479.531^**^	146.798^**^	5.037
PH	71.925	587.303^**^	1,420.834^**^	1,227.313^**^	388.226^**^	20.126
TL	3.444	25.321^**^	12.351^**^	35.350^**^	25.921^**^	0.945
TBR	0.499	5.817^**^	7.980^**^	6.545^**^	5.417^**^	0.307
CL	0.246	1.892^**^	2.920^**^	3.211^**^	1.580^**^	0.208
CG	0.004	0.974^**^	1.730^**^	3.408^**^	0.561^**^	0.128
NKr/R	0.418	15.774^**^	33.714^**^	14.309^**^	13.394^**^	0.325
NR/C	0.015	1.302^**^	2.221^**^	1.829^**^	1.105^**^	0.078
CW	4.275	1,030.117^**^	1,471.981^**^	2,155.517^**^	826.319^**^	5.045
SHW	0.027	16.420^**^	32.671^**^	37.710^**^	11.437^**^	0.732
SH%	2.069	6.824^**^	15.152^**^	4.032^**^	5.984^**^	0.620
SPY	12.320	873.697^**^	1,295.440^**^	1,657.901^**^	715.423^**^	4.375
100 SW	0.057	25.554^**^	74.081^**^	85.264^**^	11.157^**^	0.113
SL	0.001	0.008^**^	0.006^**^	0.033^**^	0.005^**^	0.001
SG	0.002	0.010^**^	0.014^**^	0.036^**^	0.007^**^	0.0001
ST	0.000	0.003^**^	0.005^**^	0.005^**^	0.002^**^	0.0001
SVI	39,724.556	832,227.331^**^	1,769,444.016^**^	1,169,860.154^**^	656,135.131^**^	9,410.735
STR	1.136	34.601^**^	53.559^**^	65.108^**^	28.080^**^	0.533
HIP	0.002	0.062^**^	0.058^**^	0.040^**^	0.065^**^	0.002
TRP	0.001	0.001^**^	0.001^**^	0.001^**^	0.001^**^	0.0001
PA	0.008	3.330^**^	2.837^**^	4.838^**^	3.212^**^	0.030

*50 DT, days to 50% tasseling; 50 DS, days to 50% silking; ASI, anthesis silking interval; CPH, cob placement height, PH, plant height; TL, tassel length; TBR, number of the tassel of branches; CL, cob length; CG, cob girth; Nkr/R, number of kernels per row; NR/C, number of rows per cob, CW, cob weight; SHW, shank weight; SH%, shelling percentage; SPY, single plant yield; 100 SW, hundred seed weight; SL, seed length; SG, seed girth; ST, seed thickness; SVI, seedling vigor index; STR, starch content; HIP, high inorganic phosphorus; TRP, tryptophan; PA, phytic acid*.

* *significant at 5%*;

** *significant at 1%*.

### Identification of Elite Parents Across Locations

**Figure 1:**
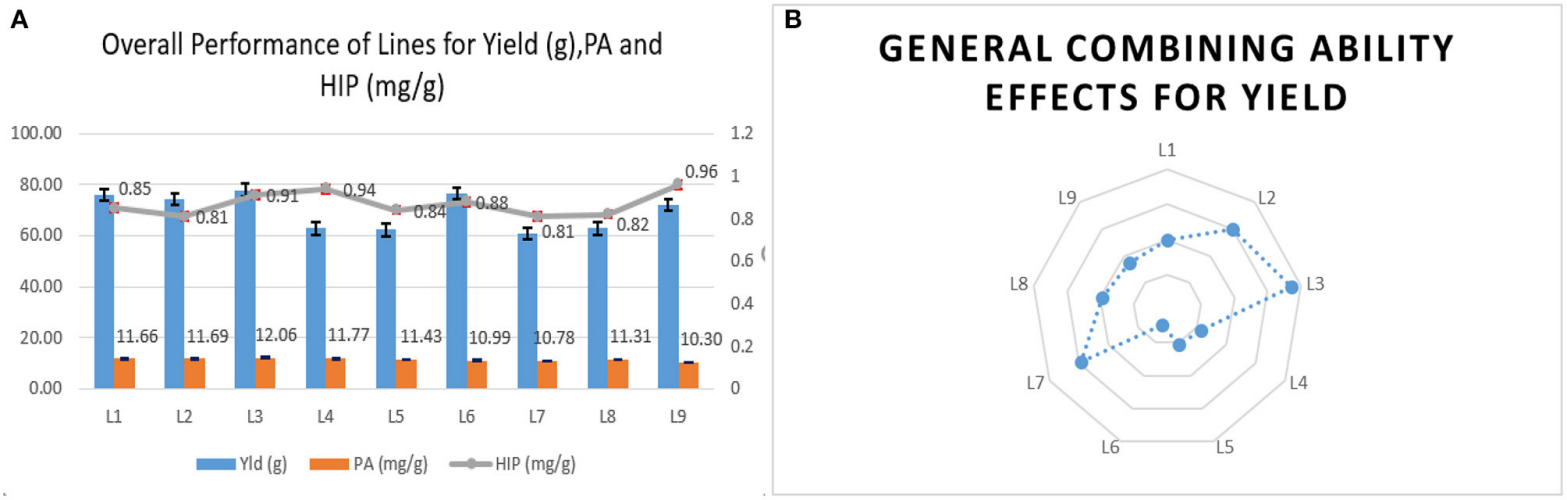
The general mean performance and combining ability of female lines. **(A)** The overall mean performance of the female lines for single plant yield (g), phytic acid (PA), and high inorganic phosphorus (HIP) in mg/g. **(B)** The overall performance of the female lines in a series of crosses (*gca*) is depicted.

The female parents selected for the study were found agronomically superior to the *lpa* testers for yield. Among lines, the mean and general combining ability was positively significant in UMI 1201 for 15 yield contributing traits in location E_1_ and 10 traits in E_2_. Although UMI 1201 had a higher mean performance for yield in E_1_, UMI 1205 surpassed this in E_2_ with a better performance in 16 attributing traits. In E_3_, there were similar performances for yield in UMI 1205. Following this, UMI 1201 had a high nonsignificant yield with a significant *gca* effect. Hence, among the lines, UMI 1205 and UMI 1201 were observed to perform better with a higher mean cum *gca* effects across locations (Figures 1A,B). Including yield, these inbreds had higher *per se* cum *gca* effects for cob weight, cob placement height, 100 seed weight, and starch content (Supplementary Material). These attributing traits were observed to be stable and higher in these inbreds, and this states their contribution toward the potential combining ability across locations (Al-Naggar et al., 2016; Kuselan, 2017). Hence, UMI 1205 and UMI 1201 could be used as female lines for producing elite hybrids in maize (Lahane et al., 2014; Kumar et al., 2016).

**Figure 2:**
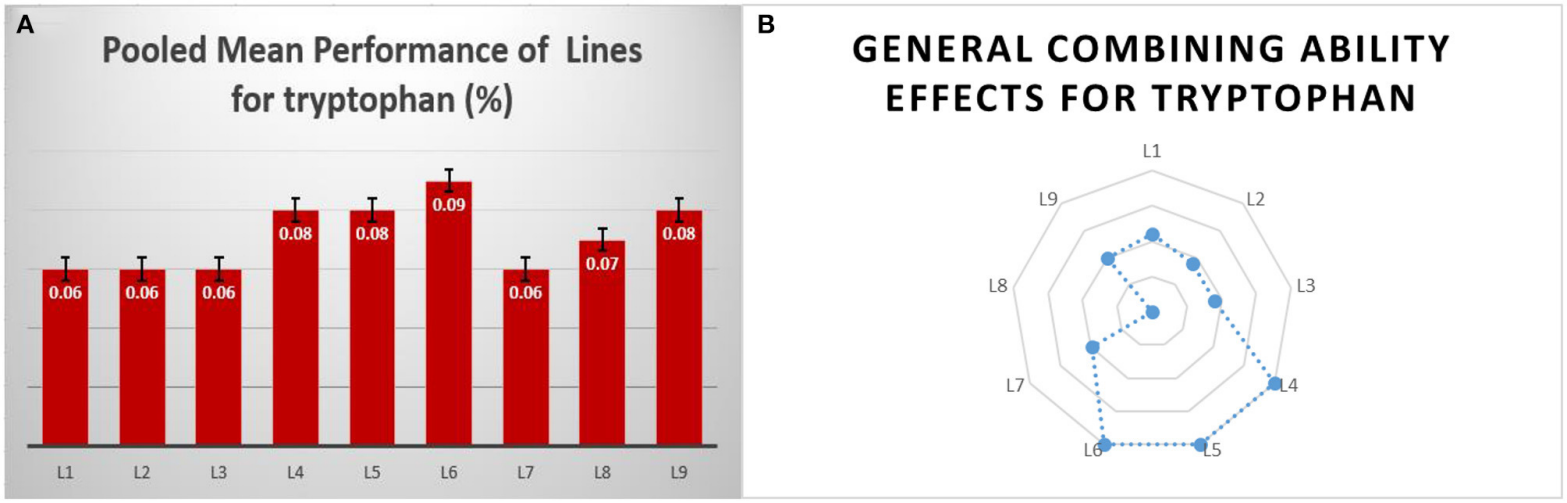
The overall pooled mean performance and general combining ability of female lines for tryptophan. **(A)** The pooled mean performance of lines for tryptophan content. **(B)** The general performance of lines in a series of crosses for tryptophan.

The identified tryptophan donors were used as lines, and among them, DMR-QPM-04-05 and DMR-QPM-01-06-2 had significant positive *gca* effects for tryptophan in E_1_ and E_3_. Although DMR-QPM-01-06-02 in E_1_ had a higher mean for tryptophan, in E_2_, it failed to exhibit a significant mean. Across locations, it is observed that DMR-QPM-04-05 had a positive significant mean and *gca* for tryptophan. The significant *gca* of this inbred across locations describes a higher heritability with less environmental influence for tryptophan (Fasahat, 2016). Hence, this line could be used as an efficient donor for tryptophan (Figures 2A,B).

The stable donors for *lpa* as testers had a lower phytate. The negative *gca* effect for PA in them is a positive indication for developing *lpa* hybrids, as they eventually reduce the PA content in a series of cross combinations. Among the eight testers, LPA-2-285, UMI 447, and UMI 467 had significantly lower mean and negative *gca* effects for PA in E_1_ and E_3_. In E_2_, except for UMI 467, the other lines had a negative *gca* for PA. Although UMI 467 was stable in lower mean performance, its reliability as a parent in reducing the phytate in hybrids by negative significant *gca* was not evident in E_2_. Hence, LPA-2-285 and UMI 447 could be used as stable potential low phytate combiners in hybridization programs (Lahane et al., 2014).

**Figure 3:**
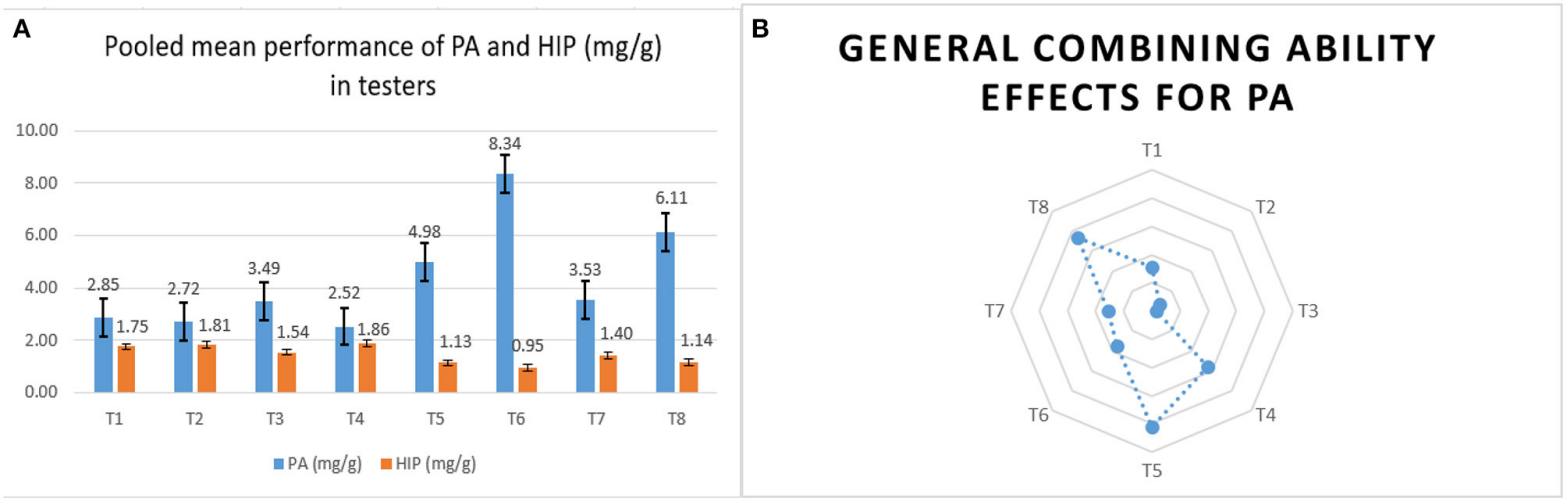
The overall mean performance and combining ability of the male testers. **(A)** The comparative pooled mean performances of PA and HIP in mg/g in male testers. **(B)** The overall performance of male testers in crosses (*gca*) for PA content.

The tester, UMI 467, had the lowest PA (2.52 mg/g) and HIP (1.86 mg/g) across locations, but it failed to reinstate its utility as a *lpa* parent in hybridization due to its positive *gca* in E_2_. Hence, UMI 467 could be used in introgression programs for developing an elite *lpa* inbred in maize. Similar findings of these parental inbreds were also reported earlier by Chandana et al. (2018) and Vengilat et al. (2019). On the whole, among the parents, the testers had a lower starch and average growth attribute than the lines due to the reduction in PA as reported by Sparvoli and Eleonora (2015). Therefore, these pleiotropic effects in *lpa* lines have to be manifested for developing elite hybrids in the future (Figures 3A,B).

### Best Performing Single Cross Hybrids Over Locations

**Figure 4:**
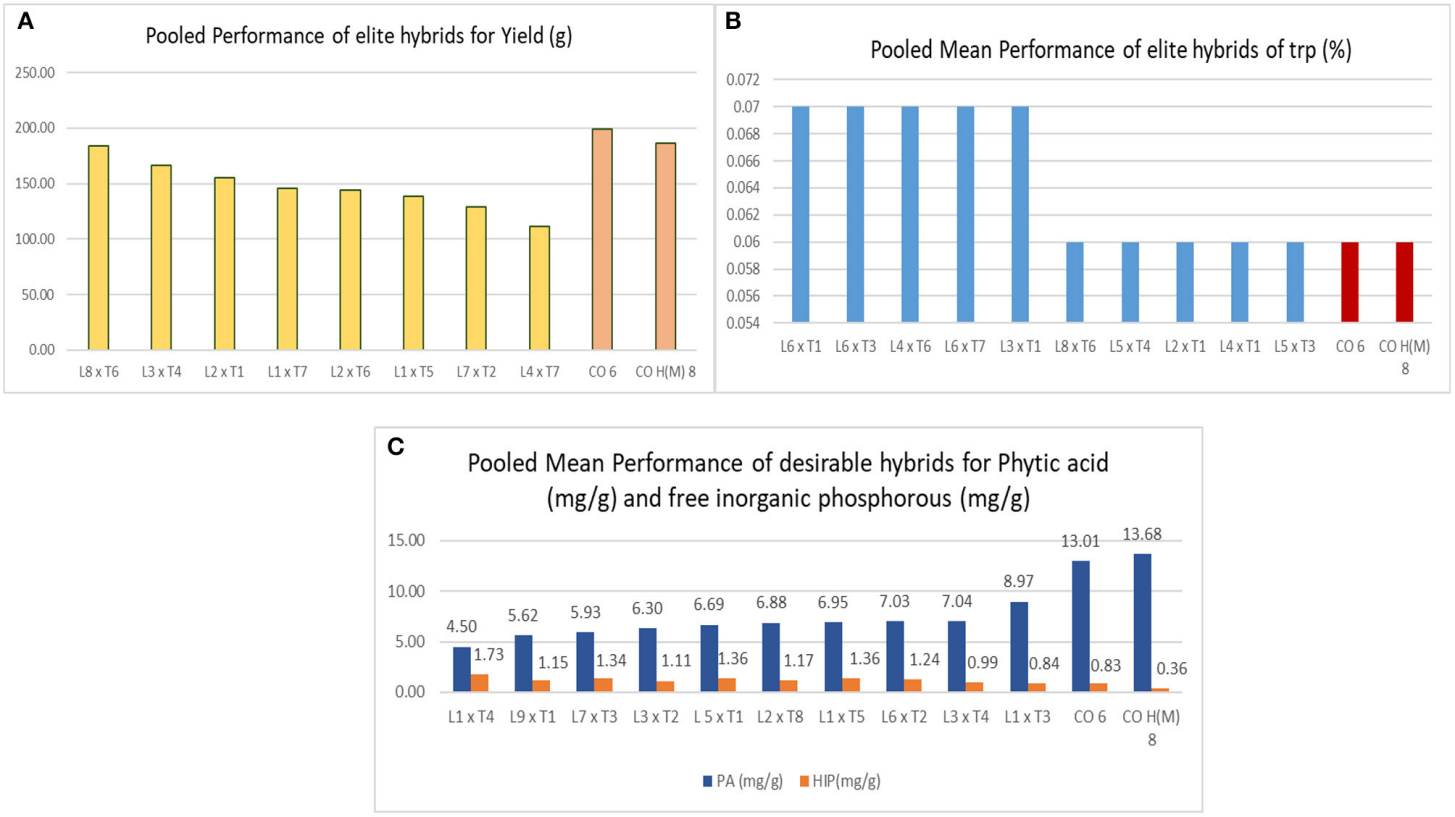
Depicts the top hybrids for the response factors, such as single plant yield, tryptophan, PA, and HIP. **(A)** The top hybrids for yield among all locations with checks highlighted in orange. **(B)** The top hybrids for tryptophan content across locations. and **(C)** the top hybrids with low PA and HIP (mg/g).

The ratio of GCA/SCA variance was lesser than unity for all traits across locations (Table 3), and this presented the preponderance of nonadditive gene action for hybrid vigor, which is observed in all the traits over locations (Malik et al., 2004; Mural, 2012; Ferreira et al., 2018). Among all the hybrids evaluated in E_1_, E_2_, and E_3_, DMR-QPM-09-13-1 × UMI 1099 recorded the highest yield and specific combining ability (*sca*) effect. Following this, DMR-QPM-06-12 × LPA-2-285, UMI 1205 × UMI 467, DMR-QPM-01-06-02 × K-155, and UMI 1201 × LPA-2-285 had a significantly higher yield and *sca* effects for yield in E_1_. The top yielders with significant *sca* effects in E_2_ after DMR-QPM-09-13-1 × UMI 1099 had slight variations from E_1_, and they were DMR-QPM-06-12 × LPA-2-285, UMI 1201 × LPA-2-285, UMI 1205 × UMI 467, and UMI 1200 × UMI 158. Similarly, in E_3_, the successive yielders and superior hybrids were DMR-QPM-06-12 × LPA-2-285, UMI 1205 × UMI 467, UMI 1200 × K-155, and UMI 1201 × UMI 1099. Hence, it is clear that DMR-QPM-09-13-1 × UMI 1099 followed by DMR-QPM-06-12 × LPA-2-285 recorded the highest mean and *sca* effect in all the environments for yield. Therefore, regarding the performance of the hybrids for yield, DMR-QPM-09-13-1 × UMI 1099, DMR-QPM-06-12 × LPA-2-285, UMI 1205 × UMI 467, UMI 1201 × LPA-2-285, DMR-QPM-01-06-02 × K-155, UMI 1200 × K-155, UMI 1200 × UMI 158, and UMI 1201 × UMI 1099 were elite in individual locations and were forwarded to stability models for identifying an ideal hybrid (Figure 4A).

The tryptophan content in hybrids was higher than checks, but none of them had a similar higher performance in other locations as observed for yield. The crosses DMR-QPM-04-05 × LPA-2-285, DMR-QPM-04-05 × UMI 447, DMR-QPM-09-13-1 × UMI 1099, DMR-QPM-01-06-02 × UMI 1099, and DMR-QPM-03-72 × UMI 467 in E_1_, UMI 1201 × LPA-2-285, DMR-QPM-01-06-02 × LPA-2-285, and DMR-QPM-03-72 × UMI 447 in E_2_, and DMR-QPM-04-05 × K-155 and UMI 1205 × LPA-2-285 in E3 recorded a higher tryptophan (0.09%) with a poor yield (Lorenz et al., 2008). Thus, all these hybrids were subjected to a stability model for identifying a stable *lpa* hybrid with higher tryptophan in them (Figure 4B).

Considering the *lpa* here, all the hybrids had a significantly lower PA than the checks, and among them, UMI 1200 × UMI 467 exhibited a lower phytate with negative *sca* in E_2_ and E_3_. In E_1_, the lowest PA was observed in DMR-QPM-11-17 × LPA-2-285, and following this, UMI 1200 × UMI 467, UMI 1200 × LPA-2-285, DMR-QPM-04-05 × LPA-2-395, and DMR-QPM-06-12 × UMI 447 had a lower *per se* cum *sca* for phytate. In E_2_ after UMI 1200 × UMI 467, the hybrids UMI 1205 × UMI 467, UMI 1201 × UMI 113, DMR-QPM-03-72 × LPA-2-285, and UMI 1200 × UMI 447 were desirable for their lower mean and negative significant *sca*. Moreover, at E_3_ with a minor difference in the order following UMI 1200 × UMI 467, the hybrids UMI 1205 × LPA-2-395, UMI 1200 × UMI 158, UMI 1200 × LPA-2-285, and DMR-QPM-11-17 × LPA-2-285 were lower in their mean with significant negative *sca* effects. Hence, the identified *lpa* hybrids from individual locations that were subjected in the stability models were DMR-QPM-11-17 × LPA-2-285, UMI 1200 × UMI 467, DMR-QPM-04-05 × LPA-2-395, DMR-QPM-06-12 × UMI 447, UMI 1205 × UMI 467, UMI 1201 × UMI 113, DMR-QPM-03-72 × LPA-2-285, UMI 1200 × UMI 447, UMI 1205 × LPA-2-395, and UMI 1200 × UMI 158 (Figure 4C). Considering all the three traits and the overall selection of hybrids based on their mean and *sca* for yield, PA and tryptophan presented a set of 17 hybrids and these were further subjected to a stability analysis in AMMI and GGE biplot (Tables 4, 5).

**Table 4:** ANOVA for pooled analysis of hybrids for yield and phytic acid (PA).

**Source**	**df**	**Phytic acid**	**Single plant yield**
Environment	2	6.373133^**^	2362.574^**^
Replication (Environment)	3	0.08075	13.05783^*^
Genotypes	73	13.22204^**^	3,112.771^**^
Environment × Genotype	146	3.545372^**^	686.1868^**^
Residuals	219	0.159932	16.42773

* *significant at 5%*;

** *significant at 1%*.

**Table 5:** The codes for the selected hybrids subjected to AMMI and GGE.

**S.No**	**Codes**	**Hybrids**	**S.No**	**Codes**	**Hybrids**
1	H1	UMI 1200 × LPA-2-285	14	H14	DMR-QPM-06-12 × LPA-2-285
2	H2	UMI 1200 × UMI 447	15	H15	DMR-QPM-06-12 × UMI 447
3	H3	UMI 1200 × UMI 467	16	H16	DMR-QPM-09-13-1 × UMI 1099
4	H4	UMI 1200 × UMI 158	17	H17	DMR-QPM-11-17 × LPA-2-285
5	H5	UMI 1200 × K-155	18	C1	CO 6
6	H6	UMI 1201 × LPA-2-285	19	C2	CO H(M) 8
7	H7	UMI 1201 × UMI 1099			
8	H8	UMI 1201 × UMI 113			
9	H9	UMI 1205 × LPA-2-395			
10	H10	UMI 1205 × UMI 467			
11	H11	DMR-QPM-01-06-02 × K-155			
12	H12	DMR-QPM-03-72 × LPA-2-285			
13	H13	DMR-QPM-04-05 × LPA-2-395			

*significant at 5%; **significant at 1%.

### Stability of the Desirable Hybrids With *lpa* and Higher Yield by AMMI and GGE Biplot

**Figure 5:**
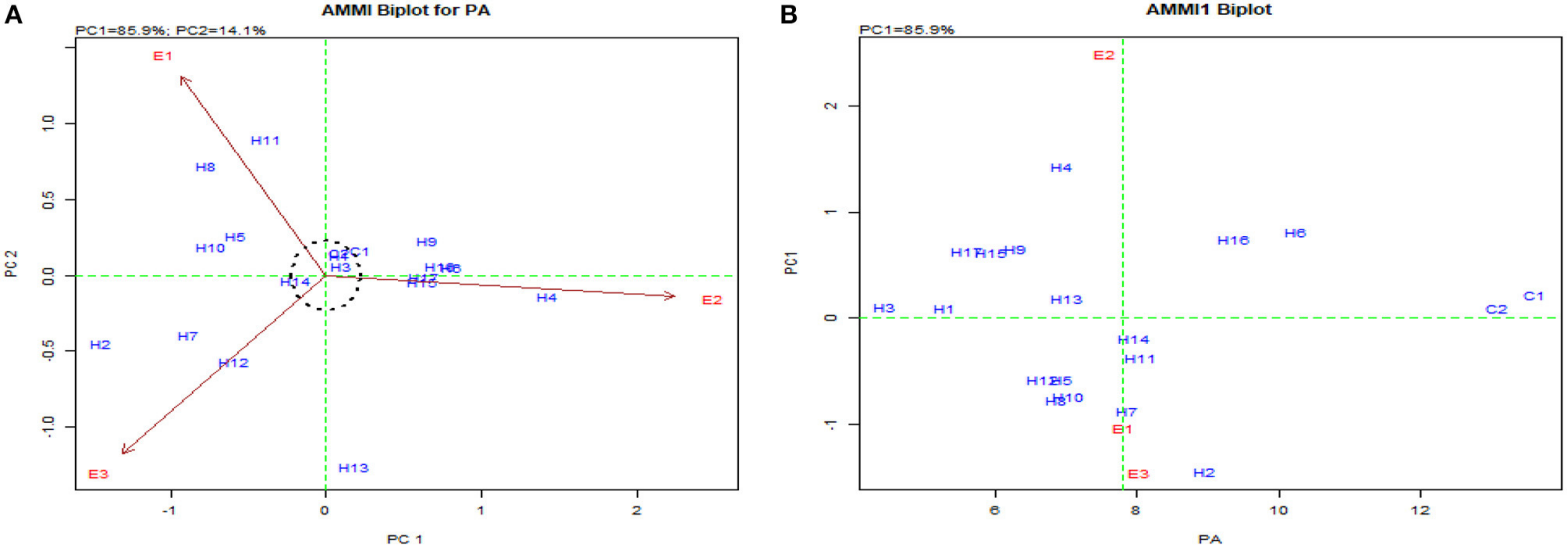
AMMI biplot for PA in the hybrids. **(A)** AMMI biplot PC1 vs. PC2 for PA and **(B)** AMMI biplot for the mean of PA vs. PC1.

The pooled ANOVA for PA and yield revealed a significant G × E interaction for 72 hybrids raised in three locations (Table 4). The efficient graphical stability models such as AMMI and GGE biplot were performed for 17 desirable hybrids identified in individual locations for yield and *lpa* from the “Best performing single cross hybrids over locations” section to overcome the noise in graphs as reported by Gauch (1992) (Table 5). The AMMI biplot for PA had two principal components (PCs), with PC1 encountering a maximum G × E interaction of 85.90%, followed by PC2 with 14.10%. From AMMI, UMI 1200 × UMI 467, UMI 1200 × LPA-2-285, CO 6, CO H(M) 8, and DMR-QPM-06-12 × LPA-2-285 were stable for their PA with desirable PC scores falling inside the circle of origin (Figure 5A). Among these stable hybrids, UMI 1200 × UMI 467 and UMI 1200 × LPA-2-285 had a *lpa*, while the rest of them were stable for their higher phytate ranges (Figure 5B). The hybrids that exactly fall on the origin of the circle possess a consistent performance for PA, and in this, UMI 1200 × UMI 467 was found to be stable for *lpa* across locations (Yates and Cochran, 1938).

**Figure 6:**
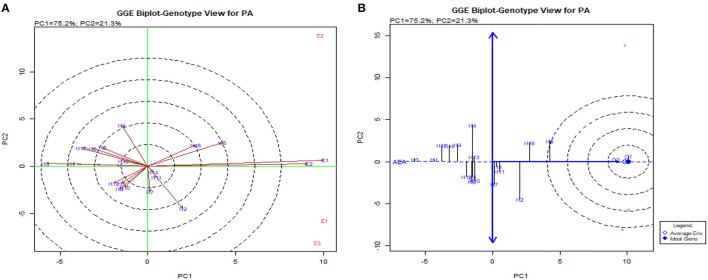
GGE biplot for PA in hybrids. **(A)** The genotype view of the hybrids in the GGE plot. **(B)** The ideal genotype plot for hybrids in the GGE biplot.

**Figure 7:**
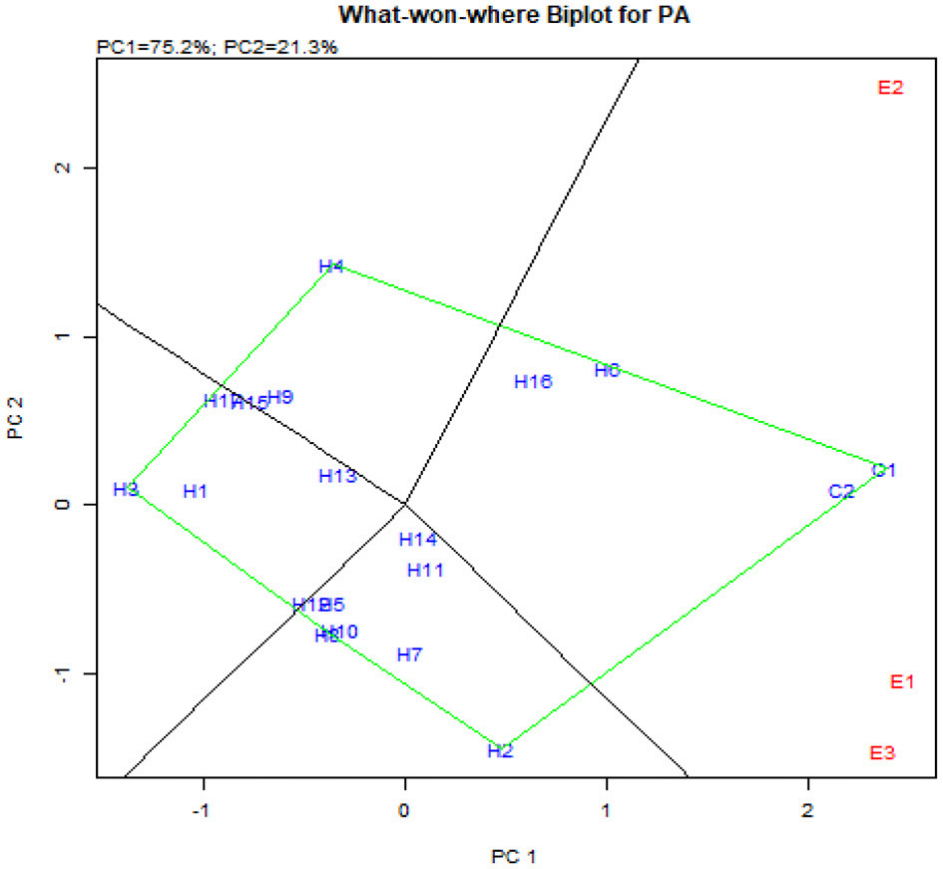
What-won-where biplot for PA depicting the grouping of environments and genotypes based on their G × E interaction.

The GGE biplot was similar to AMMI with 75.20% in PC1 and 21.30% in PC2 for G × E interactions (Balestre et al., 2009). The genotype view of the GGE biplot exhibited the stable performance of UMI 1200 × UMI 467 and UMI 1200 × LPA-2-285 with their arrows falling in the negative quadrant near the PC2 axis (Figure 6). The ideal genotype plot in GGE depicts the check CO 6 as ideal for its higher stable PA. For stable *lpa*, UMI 1200 × UMI 467 that fell on a similar origin in the negative quadrant is ideal (Balestre et al., 2009). For PA in the what-won-where biplot, E_1_ and E_3_ (Figures 6, 7) had a similar action toward the crosses raised, and this stated that any one of these locations could be omitted in other multilocational trials for studying the environmental variations in PA (Yates and Cochran, 1938).

**Figure 8:**
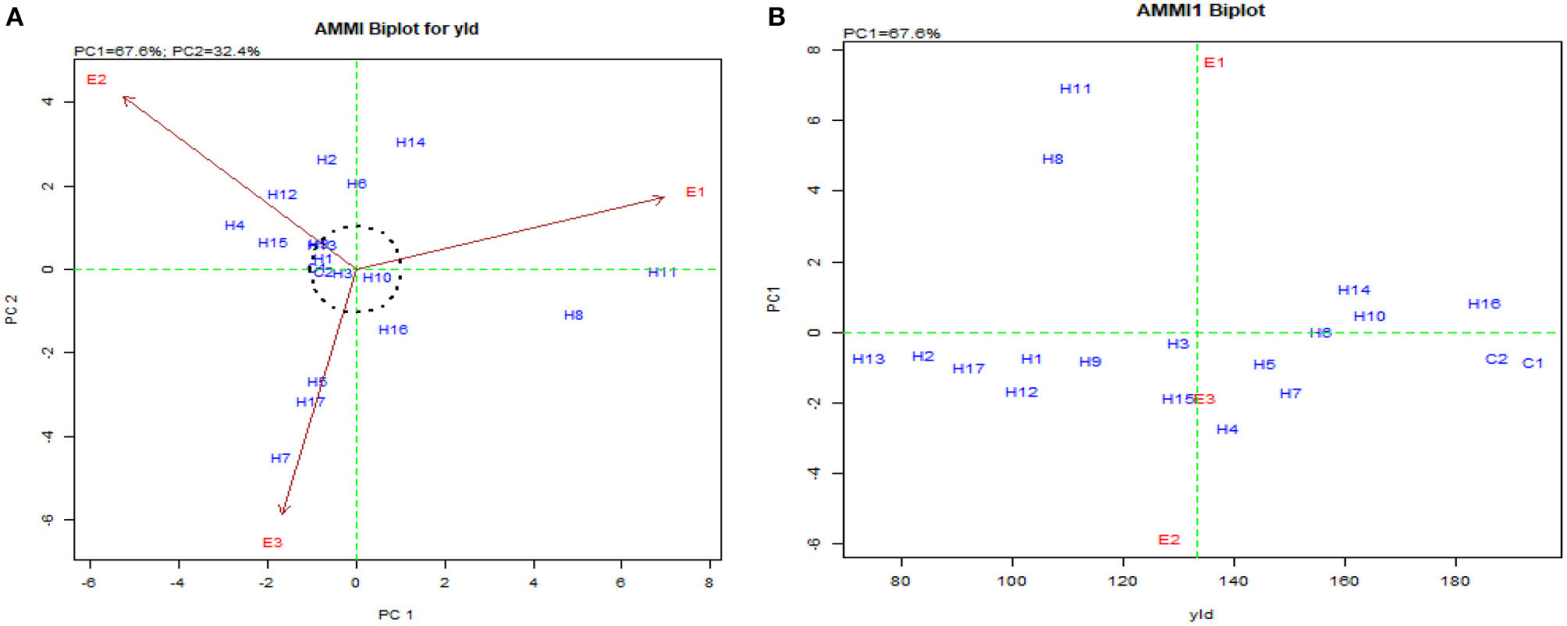
AMMI biplot for single plant yield in the hybrids. **(A)** AMMI biplot PC1 vs. PC2 for single plant yield and **(B)** AMMI biplot for the mean of single plant yield (yld) vs. PC1.

The stability for the second response factor in AMMI biplot for yield presented the hybrids such as UMI 1205 × UMI 467, UMI 1200 × UMI 467, CO H(M) 8, CO 6, and DMR-QPM-04-05 × LPA-2-395 as stable. They were found near the origin within the concentric circle (Balestre et al., 2009). Although these hybrids were stable, the highest stable performance for yield was exhibited by the checks CO 6 and CO H (M) 8, which were released hybrids (Nallathambi et al., 2012). The potent yielder, DMR-QPM-09-13-1 × UMI 1099, had stable performances in E_1_ and E_3_, but due to the prevailing rainfed situations in E_2_, a reduction in yield was observed (Pramitha et al., 2020). From the overall mean and PC performance in AMMI for yield (Figure 8A), DMR-QPM-09-13-1 × UMI 1099 with the highest mean and a higher PC1 in the positive quadrant near the x-axis could be identified as an elite stable hybrid (Figure 8B).

**Figure 9:**
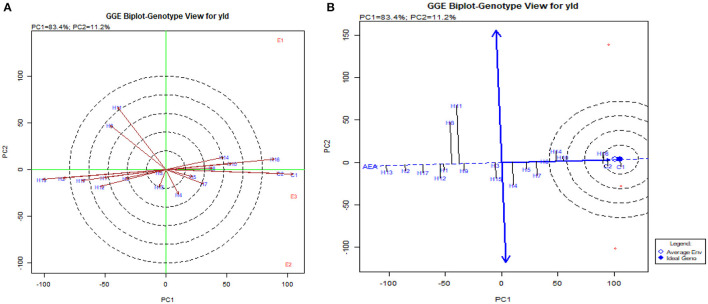
GGE biplot for single plant yield in hybrids. **(A)** The genotype view of the hybrids for single plant yield in the GGE plot. **(B)** The ideal genotype plot of hybrids for single plant yield in the GGE biplot.

**Figure 10:**
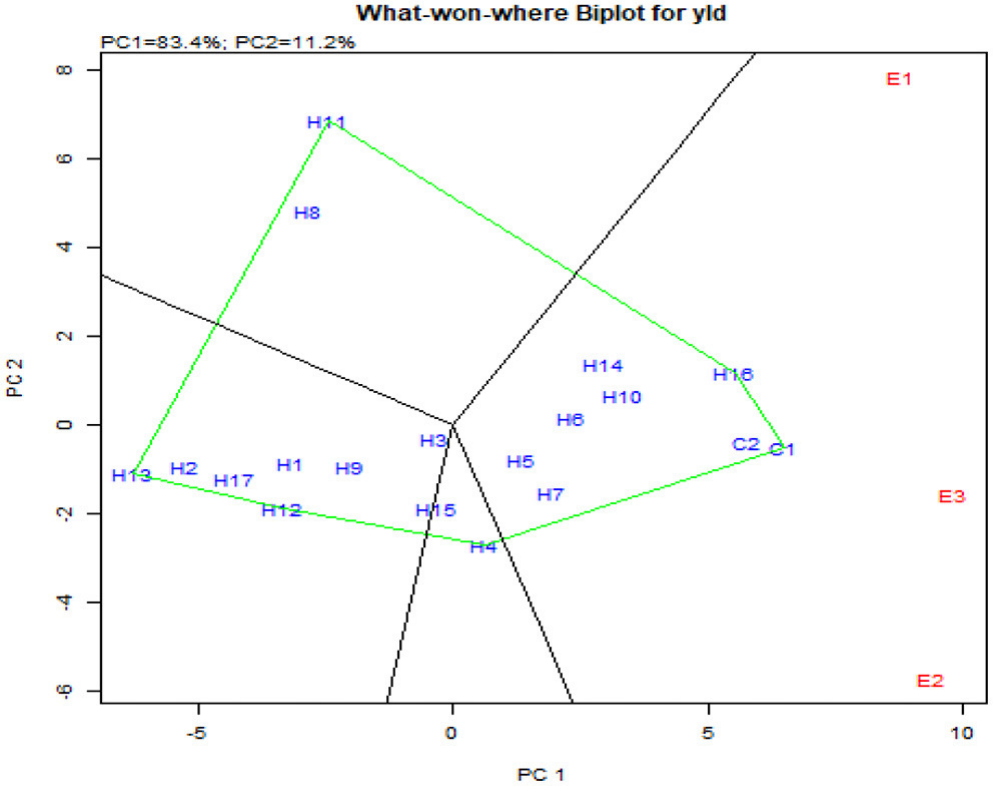
What-won-where biplot for single plant yield depicting the grouping of environments and genotypes based on their G × E interaction.

Concomitantly, the GGE biplot also had similar results with CO 6, CO H(M) 8, DMR-QPM-09-13-1 × UMI 1099, and UMI 1205 × UMI 467 as stable high-yielding genotypes in the positive quadrant of the x-axis (Figure 9A). Also, as a confirmation for ideal hybrid in the GGE biplot, CO 6, CO H(M) 8, and DMR-QPM-09-13-1 × UMI 1099 were falling near the origin of the ideal axis (Akhtar et al., 2018). All the environments in the what-won-where plot for yield had a differential interaction toward the hybrids, and this states that other multilocational trials for yield could be conducted in larger plots across these places in the future (Figures 9, 10).

### Selection of Desirable Hybrids for Evaluation

All the hybrids obtained had a higher tryptophan and lower PA than the checks. Although the hybrids were superior to the checks in terms of nutritional quality, none of them were found to surpass the checks for yield and starch (Supplementary Tables 8–23). These effects in the overall reduction for yield and starch were due to the significant positive correlation of yield with PA and starch (Pramitha et al., 2019). Since PA is involved in the myoinositol pathway that includes starch synthesis, its reduction has effected altered grain starch content in hybrids. These pleiotropic effects of PA on yield attributing traits were coherently reported by Brankovic et al. (2016) in wheat. Consequently, across locations, all the hybrids exhibited a significant G × E interaction for PA and yield (Balestre et al., 2009).

In addition to this, as the attributing traits were varying across locations, the tryptophan in hybrids also varied, whereas they were higher than the checks that were used. Hence, to identify a desirable hybrid with *lpa* and tryptophan, the preliminary selection indices were focused initially on dissecting a stable hybrid with *lpa* and yield.

From these selected stable hybrids from AMMI and GGE biplots, the higher tryptophan hybrids were then scrutinized to identify a desirable genotype with all three response factors. This variation of tryptophan across locations was also reported by Zaidi et al. (2008), and the top-performing hybrids for tryptophan from individual locations in the “Best performing single cross hybrids over locations” section have to be evaluated simultaneously in the future to identify the attributing traits for its stability.

**Figure 11:**
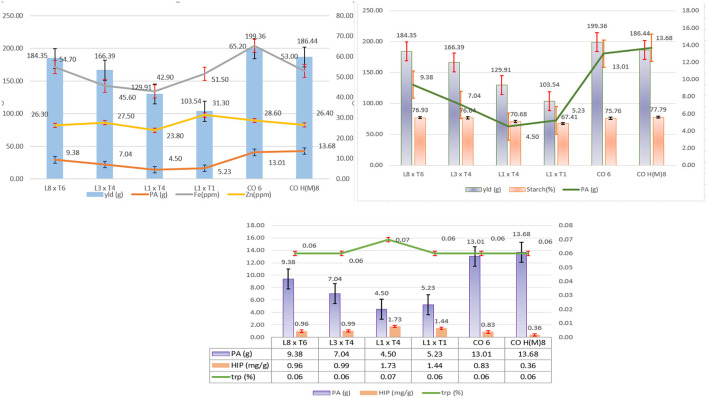
The overall performance of the selected and identified hybrids with standard checks CO 6 and CO H(M) 8 for single plant yield (g), PA (mg/g), iron (ppm), zinc (ppm) starch (%), HIP (mg/g), and tryptophan (%). Comparative performances of yield with PA, HIP, tryptophan, iron zinc, and starch are depicted.

Among the 72 hybrids raised in locations, four of them, namely, DMR-QPM-09-13-1 × UMI 1099, UMI 1205 × UMI 467, UMI 1200 × UMI 467, and UMI 1200 × LPA-2-285, were stable in AMMI and GGE biplots for their yield and PA with a higher tryptophan content (Figure 11) (Yates and Cochran, 1938). The buffering in yield among all hybrids was noticed due to the highest interaction of E_2_ (Oomah et al., 1996; Kayodé Polycarpe et al., 2006; Balestre et al., 2009). Even the highest yielder DMR-QPM-09-13-1 × UMI 1099 had a reduction in its yield due to the prevailing rainfed situations in E_2_ (Zaidi et al., 2008; Brankovic et al., 2016). The PA in crops is known to regulate anthesis, pollination, seedling vigor, starch accumulation, and stress tolerance in plants (Pramitha et al., 2021). Thus, its reduction by *lpa* donors has caused the average performance of hybrids as compared to the checks across locations. Thereby, the compensation of these negative pleiotropic effects such as reduced starch, cob girth, and single plant yield from the *lpa* donors has to be reinforced in upcoming biofortification trials of maize (Bregitzer et al., 2008; Lorenz et al., 2008).

From the superior hybrids, the highest yielder DMR-QPM-09-13-1 × UMI 1099 consisted of parents in a combination of low *gca* × high *gca* effect (Supplementary Tables 8–23). This explains that the heterotic expression has been resulted by combined additive effects of favorable alleles in UMI 1099 and an epistatic effect of desirable alleles in DMR-QPM-09-13-1 (Fasahat, 2016). Similar heterotic combinations of parents with varying *gca* effects were observed in UMI 1200 × UMI 467 and UMI 1200 × LPA-2-285. Such kinds of heterotic effects were also reported by Sun et al. (2018). The second highest yield performer, UMI 1205 × UMI 467, had a good favorable *gca* combination, comprising two good *gca* parents in its cross. This is in accordance with several findings, including Uddin et al. (2006) and Kumar et al. (2016), and the higher yield is ascribed due to the additive gene action of the favorable alleles from both parents.

The most successful hybrid attained from these crosses with *lpa* and higher tryptophan was UMI 1200 × UMI 467. Among all the hybrids, this had the lowest PA (4.92 mg/g) and the highest tryptophan content (0.07%) with a stable yield (129.91 g) across locations. Hence, this study confirms the simultaneous improvisation of PA and tryptophan in maize. Thus, the parental inbreds used here were grouped into heterotic groups for further use as a reference in breeding programs.

### Analyzing Nutritional Parameters of the Selected Hybrids

Among all the screened hybrids, UMI 1200 × UMI 467 followed by UMI 1200 × LPA-2-285 had the lowest PA with HIP, moderate starch, and tryptophan across locations. Among these two, UMI 1200 × UMI 467 had a uniform yield, *lpa*, and highest tryptophan. Reinforcing yield for commercialization, the hybrid DMR-QPM-09-13-1 × UMI 1099 followed by UMI 1205 × UMI 467 had the highest stable yield with medium PA and tryptophan. These high yielders with moderate *lpa* also had a higher starch content than the rest of them. To conclude, all the four hybrids, namely, DMR-QPM-09-13-1 × UMI 1099, UMI 1205 × UMI 467, UMI 1200 × UMI 467, and UMI 1200 × LPA-2-285 identified from this study also had a higher Fe and Zn than the checks (Figure 11). Hence, these hybrids could be forwarded in other trials for analyzing their elite performance and PA: mineral ratio to afford an enhanced bioavailability of minerals in maize diets (Hambidge et al., 2005).

### Heterotic Grouping of Parental Inbreds

The heterotic grouping of inbreds for PA facilitates maintaining the parental inbreds in their designated pools to recreate varying combinations for PA in near future. This reduces the necessity of analyzing their combining ability performances repeatedly. In this study, the method that relies on the combining ability adopted by Legesse et al. (2009) was followed. Based on this, two testers with diverse *gca* effects for PA have been selected (Table 5). The testers for the first group were UMI 447 and UMI 158, while those for the second group were UMI 467 and UMI 113. These testers had extreme negative and positive significant *gca* for phytate across locations. The lines that exhibit a significant negative *sca* with one of the testers are grouped with the tester with whom it established a negative *sca*. Those that show a negative *sca* with both of them fall under the combined group of both testers and *vice versa* are rejected (Table 6). This favors varying lines with the testers for producing a lower PA content in the hybrids.

**Table 6:** General and specific combining ability of parents and hybrids for PA across locations.

**Phytic acid**									
**Lines\Testers**	**T1**	**T2**	**T3**	**T4**	**T5**	**T6**	**T7**	**T8**	**Lines**
L1	−1.70^**^	2.07^**^	−1.07^**^	−1.72^**^	2.06^**^	0.59^*^	−1.74^**^	1.52^**^	−0.71^**^
L2	4.36^**^	2.38^**^	−0.53^*^	−0.79^**^	0.14	−2.27^**^	0.86^**^	−4.14^**^	0.00
L3	−0.69^**^	0.86^**^	0.80^**^	−3.09^**^	−1.31^**^	−0.88^**^	0.62^*^	3.69^**^	−0.27^**^
L4	−1.72^**^	−1.25^**^	0.21	4.69^**^	−1.61^**^	1.56^**^	−1.00^**^	−0.88^**^	0.06
L5	−1.57^**^	−0.17	−0.61^*^	0.21	0.65^*^	0.20	0.06	1.22^**^	−1.02^**^
L6	2.83^**^	−0.03	−0.18	−0.26	0.44	0.62^*^	−1.95^**^	−1.48^**^	0.39^**^
L7	−0.97^**^	−2.06^**^	−0.27	1.07^**^	1.73^**^	−1.51^**^	3.86^**^	−1.85^**^	0.57^**^
L8	0.26	−0.29	1.05^**^	0.45	−1.85^**^	2.30^**^	−1.36^**^	−0.55^*^	0.90^**^
L9	−0.79^**^	−1.51^**^	0.59^*^	−0.56^*^	−0.25	−0.60^*^	0.64^*^	2.47^**^	0.08
Testers	−0.22^**^	−0.82^**^	−0.93^**^	0.40^**^	1.05^**^	−0.12	−0.22^**^	0.85^**^	
SE (*gca* for line)		0.043			**SE (sca effects)**			0.122	
SE (*gca* for tester)		0.040							

* *Significant at 5%*;

** *significant at 1%*.

By adopting this method, two groups, namely, 1. UMI 447 and UMI 158 and 2. UMI 467 and UMI 113, were formed. The sum of the *sca* and the mean values for all the crosses in each group were estimated separately, and this revealed UMI 447 and UMI 158 as a desirable pool incorporating a lower mean and negative *sca* for all line × tester phytate combinations (Table 7). Hence, this group could be identified as a favorable heterotic pool for preserving the parental inbreds for *lpa*. This could be further elaborated by including more stable *lpa* inbreds and combiners in the future.

**Table 7:** The grouping of the lines in the tester groups based on their combining ability.

**Heterotic group-I**		**Heterotic group-II**		
**UMI 158 & UMI 447 Pool I**		**UMI 467 & UMI 113 Pool-II**		
UMI 158 sub-group	UMI 447 sub-group	UMI 467 sub-group	UMI 113sub- group	UMI 467 & UMI 113 sub-group
DMR-QPM-09-13-1	UMI 1200	UMI 1200	DMR-QPM-01-06-02	UMI 1201
DMR-QPM-11-17	UMI 1201	UMI 1205	DMR-QPM-06-12	DMR-QPM-04-05
UMI 1205	DMR-QPM-03-72	DMR-QPM-11-17	DMR-QPM-09-13-1	
DMR-QPM-01-06-02	DMR-QPM-04-05			
	DMR-QPM-06-12			

## Conclusion

The study focuses on simultaneous improvisation with reduced PA and increased tryptophan content in maize grain. The identified hybrids were found nutritionally superior with a compromise in their yield as compared to the check hybrids. Hence, introgressing elite parents such as UMI 1205 and UMI 1201 with *lpa* and higher tryptophan will lead to developing superior parents with desirable traits for crossing. Furthermore, implying these Near Isogenic Line (NILs) in the production of hybrids would be rewarding to overcome the yield constraints in commercialization. Also, as drastic reductions in phytate were found to alter the yield, these high-yielding moderate phytate and tryptophan hybrids, such as DMR-QPM-09-13-1 × UMI 1099 and UMI 1205 × UMI 467, could be evaluated for their efficiency in nutrient uptake by analyzing their mineral ratios in feeds. Therefore, this heterotic pool along with identified hybrids could be maintained as a reference population in the biofortification of maize, and it could help in framing the phases for producing elite *lpa* cum tryptophan hybrids in the future.

## Data Availability Statement

The original contributions presented in the study are included in the article/Supplementary Material, further inquiries can be directed to the corresponding author.

## Author Contributions

All authors listed have made a substantial, direct, and intellectual contribution to the work and approved it for publication.

## Conflict of Interest

The authors declare that the research was conducted in the absence of any commercial or financial relationships that could be construed as a potential conflict of interest.

## Publisher's Note

All claims expressed in this article are solely those of the authors and do not necessarily represent those of their affiliated organizations, or those of the publisher, the editors and the reviewers. Any product that may be evaluated in this article, or claim that may be made by its manufacturer, is not guaranteed or endorsed by the publisher.
