# Supplementary material for: Stability Analysis and Heterotic Studies in Maize (Zea mays L.) Inbreds to Develop Hybrids With Low Phytic Acid and High-Quality Protein
Source: Front Plant Sci. 2022 Jan 19;12:781469. doi: 10.3389/fpls.2021.781469 (PMC8823190; doi:10.3389/fpls.2021.781469)
Supplement: Supplementary file 1 [file Data_Sheet_1.PDF]

Table 8. Mean performance of parents raised in environment-1

PARENTS	50 DT	50 DS	ASI	CPH	PH	TL	TBR	CL	CG	NKr/R	NR/C	CW
L1	54.00	57.00	3.00	87.00	167.00	29.00	10.00	12.70	11.32	21.75	11.66	94.48
L2	57.00	60.00	3.00	84.83	158.83	33.67*	10.00	13.10	11.53	22.16	13.16	100.82**
L3	53.50	56.50	3.00	96.50*	172.17**	32.00	11.34	12.60	12.38	24.34	12.67	93.90
L4	53.00	57.00	4.00	91.83	160.83	32.17	11.66	14.45	10.70	28.50**	12.00	74.07
L5	51.50*	54.50**	3.00	88.33	157.67	35.00**	15.00*	15.61	12.48	27.34*	12.34	77.01
L6	53.00	56.50	3.50	76.11	138.89	23.39	11.22	15.43	13.09	24.00	15.55**	94.69
L7	50.50**	53.50**	3.00	99.50**	176.17**	26.66	14.66*	16.50	12.88	23.84	12.34	78.00
L8	54.00	57.00	3.00	79.83	152.50	28.16	12.84	16.75*	12.48	26.84	10.84	78.72
L9	49.00**	53.50**	4.50	79.72	149.39	30.11	11.34	16.60*	12.21	26.78	12.62	93.17
**MEAN**	**52.83**	**56.17**	**3.33**	**87.07**	**158.16**	**30.02**	**12.01**	**14.86**	**12.12**	**25.06**	**12.58**	**87.21**
T1	48.50**	52.00**	3.50	77.57	140.24	28.93	11.05	14.68**	10.31	20.76	12.03	63.78
T2	54.00	57.00	3.00*	82.19	162.58**	34.75**	9.63	11.57	10.09	21.18	12.13	58.81
T3	54.00	58.50	4.50	80.94	144.60	29.60	10.30	10.43	9.64	22.89*	11.33	67.82
T4	53.50	57.50	4.00	79.93	141.79	33.65*	10.22	10.61	8.34	18.81	10.10	47.46
T5	48.00**	53.00**	5.00	81.16	144.49	32.28	12.46	12.87	11.34*	19.93	12.58	65.43
T6	56.50	60.00	3.50	80.53	143.94	31.55	13.39*	12.35	11.16	20.64	12.74*	76.24**
T7	48.00**	53.00**	5.00	61.83	117.67	26.17	8.00	10.50	8.75	12.66	9.84	34.95
T8	49.00**	53.00**	4.00	82.60	165.32**	31.09	13.86*	12.80	12.57**	20.66	12.36	68.42*
**MEAN**	**51.44**	**55.50**	**4.06**	**78.35**	**145.08**	**31.00**	**11.12**	**11.98**	**10.28**	**19.69**	**11.64**	**60.32**
**CD 0.05**	1.03	1.05	0.87	8.97	11.77	3.56	2.12	1.73	1.05	2.12	1.03	8.05
**CD 0.01**	1.37	1.40	1.16	11.92	15.63	4.73	3.14	2.30	1.46	2.82	1.37	10.69

Table 8. Cont.,

PARENTS	SHW	SH%	SPY	100 SW	SL	SG	ST	SVI	STR	HIP	TRP	PA
L1	17.13	81.87*	77.35*	30.50**	1.05**	0.70*	0.40	3193.04	61.24	0.82	0.05	13.32
L2	19.21*	80.96	81.61**	28.00**	1.03**	0.62	0.39	4160.22	61.97	0.82	0.07	12.39
L3	13.25	85.89**	80.65**	26.16**	0.75	0.59	0.42*	3937.82	66.81*	0.84	0.06	12.04
L4	14.25	80.76	59.82	17.50	0.80	0.47	0.30	4106.73	66.47*	0.88	0.09**	12.13
L5	18.92	75.41	58.09	17.23	0.73	0.59	0.35	3801.93	69.41**	0.91	0.07	11.91
L6	19.02	79.90	75.66	20.34	0.91	0.66	0.44*	4376.57**	65.63	0.89	0.09**	11.23
L7	17.41	77.69	60.59	20.60	0.84	0.65	0.42*	4162.97	61.73	0.91	0.05	11.51
L8	18.97	75.90	59.74	20.57	0.72	0.62	0.33	4237.77	61.74	0.81	0.06	10.97
L9	19.26*	79.37	73.95	21.90	0.77	0.73**	0.35	4271.84*	59.57	0.88	0.08**	10.80**
**MEAN**	**17.49**	**79.75**	**69.72**	**22.53**	**0.84**	**0.62**	**0.37**	**4027.65**	**63.84**	**0.86**	**0.07**	**11.81**
T1	17.72*	72.23	46.07	18.44	0.86*	0.66	0.26	1311.50	56.67	1.54	0.05	2.84**
T2	16.14	72.53	42.67	16.61	0.73	0.61	0.33	1570.29	50.54	1.76**	0.05	2.71**
T3	18.40	72.88	49.42	19.06	0.72	0.65	0.33	1050.42	55.02	1.48	0.05	3.45
T4	14.10	70.28	33.36	17.55	0.66	0.63	0.36	502.98	50.61	1.84**	0.05	2.52**
T5	17.27	73.60	48.15	19.20	0.70	0.64	0.41**	1919.32	60.52*	1.34	0.05	5.75
T6	16.63	78.18**	59.63**	22.66**	0.81	0.64	0.41**	1742.54**	70.57**	1.28	0.05	8.46
T7	10.06	71.19	24.89	16.18	0.72	0.72*	0.31	1926.13**	55.02	1.67*	0.05	1.55**
T8	16.82	76.26*	52.21*	20.07	0.73	0.59	0.36	403.27	61.62*	1.01	0.06**	5.39
**MEAN**	**15.89**	**73.27**	**44.42**	**18.72**	**0.74**	**0.64**	**0.34**	**1303.31**	**57.57**	**1.49**	**0.05**	**4.08**
**CD 0.05**	1.72	2.76	7.61	1.39	0.11	0.07	0.04	225.16	2.26	0.17	0.01	0.73
**CD 0.01**	2.30	3.67	10.11	1.84	0.15	0.10	0.07	299.08	3.00	0.22	0.01	0.97

Table 9. Mean performance of parents raised in environment-2

PARENTS	50 DT	50 DS	ASI	CPH	PH	TL	TBR	CL	CG	NKr/R	NR/C	CW
**L1**	54.00	57.00	3.00	78.00	148.75**	23.00	10.25	13.32	11.34	21.11	12.17	87.57*
**L2**	57.00	60.00	3.00	85.67**	158.50**	24.00	8.17	12.17	10.93	20.79	12.17	80.06
**L3**	53.50	56.50	3.00	82.84**	150.83**	24.25	6.17	14.64**	10.93	21.91	12.17	84.21
**L4**	52.00	54.50**	2.50**	63.34	97.50	26.50	9.50	11.87	9.63	22.92	12.67	66.32
**L5**	51.50**	54.50**	3.00	67.33	128.67	30.67**	11.34	11.92	10.34	23.76	12.00	75.01
**L6**	53.00	57.00	4.00	74.50	132.00	28.59	11.00	13.52	11.19	24.57	12.50	85.58
**L7**	52.00	54.00**	2.00**	60.00	114.84	28.50	10.09	12.34	8.75	21.67	12.34	71.04
**L8**	53.00	57.00	4.00	69.17	118.67	31.67**	12.84**	14.77**	12.57**	25.83**	12.50	76.34
**L9**	49.00**	53.50*	4.50	72.00	124.08	24.00	10.34	12.33	10.17	26.97**	12.00	81.46
**MEAN**	**52.77**	**56.00**	**3.22**	**72.53**	**130.42**	**26.79**	**9.96**	**12.98**	**10.64**	**23.27**	**12.27**	**78.61**
**T1**	48.50**	51.50**	3.00*	84.17**	140.67**	29.83**	12.50	9.59	8.34	20.33	12.17	56.00
**T2**	54.00	57.00	3.00*	64.83	96.38	24.33	8.67	10.42	8.32	19.24	12.00	48.92
**T3**	55.50	59.00	3.50	41.33	72.83	19.75	12.33	10.34	9.50	22.84**	11.84	59.87
**T4**	51.50	57.50	6.00	40.50	83.09	17.09	8.34	9.50	7.67	15.90	9.67	32.47
**T5**	49.50**	53.50**	4.00	72.00	119.50	25.75	13.67	10.17	8.00	22.17**	12.50*	55.06
**T6**	53.00	57.00	4.00	71.17	141.23**	24.00	15.29**	12.78**	9.47	22.49**	12.00	75.66**
**T7**	49.00**	52.00**	3.00*	80.34**	146.50**	24.09	14.50**	8.32	7.74	16.83	12.00	51.74
**T8**	53.00	56.00	3.00*	65.59	127.21**	24.84	11.00	11.50	10.84**	20.07	11.84	70.33**
**MEAN**	**51.75**	**55.43**	**3.68**	**64.98**	**115.92**	**23.70**	**12.03**	**10.32**	**8.73**	**19.98**	**11.75**	**56.25**
**CD 0.05**	0.75	0.93	0.64	7.00	8.20	2.31	1.68	1.32	1.35	1.52	0.70	7.41
**CD 0.01**	1.00	1.23	0.85	9.30	10.89	3.07	2.24	1.76	1.80	2.02	0.94	9.85

Table 9. Cont.,

PARENTS	SHW	SH%	SPY	100 SW	SL	SG	ST	SVI	STR	HIP	TRP	PA
**L1**	17.58*	79.93	70.00	27.27**	0.80*	0.62	0.52**	3186.31	70.33	0.93*	0.06	9.90**
**L2**	17.35	78.33	62.71	24.81**	0.77	0.57	0.30	4140.74	71.34	0.87	0.06	11.33
**L3**	12.45	85.21*	71.77*	26.93**	0.72	0.74**	0.40	3936.14	63.55	0.79	0.05	13.09
**L4**	10.36	84.38	55.96	19.27	0.79	0.69**	0.29	4137.02	72.48	0.65	0.07	11.78
**L5**	11.83	84.23	63.18	22.17	0.85**	0.53	0.34	3791.87	72.24	0.84	0.09**	12.01
**L6**	15.73	81.62	69.85	22.75	0.75	0.60	0.40	4384.53**	73.40*	0.83	0.08**	11.45
**L7**	14.28	79.90	56.76	21.25	0.63	0.50	0.37	4182.47	72.61	0.94**	0.07	9.97**
**L8**	15.40	79.83	60.94	18.88	0.60	0.53	0.37	4311.53	70.27	0.80	0.08**	12.11
**L9**	10.35	87.29**	71.11	21.97	0.87**	0.80**	0.33	4280.77	71.89	0.90	0.07	10.38**
**MEAN**	**13.92**	**82.30**	**64.69**	**22.80**	**0.75**	**0.61**	**0.36**	**4039.03**	**70.89**	**0.83**	**0.068**	**11.33**
**T1**	10.25	81.68**	45.75	18.50	0.89**	0.56	0.22	1305.48	55.09	1.53	0.06	2.87**
**T2**	9.38	80.83	39.54	17.13	0.77	0.60	0.27	1866.32**	51.23	1.82**	0.05	2.74**
**T3**	12.18	79.66	47.69	17.66	0.73	0.63**	0.30	557.32	59.22	1.42	0.05	3.57
**T4**	7.77	76.09	24.70	16.09	0.60	0.57	0.30	1040.95	50.35	1.88**	0.06	2.52**
**T5**	12.85	76.67	42.21	15.24	0.70	0.50	0.32	1919.93**	52.94	1.54	0.07**	3.75
**T6**	16.19*	78.61	59.47**	22.04**	0.83**	0.60	0.30	1723.52**	70.90**	1.14	0.06	7.91
**T7**	15.75*	69.57	35.99	17.83	0.69	0.50	0.30	1924.65**	50.72	1.35	0.07**	5.02
**T8**	14.17	79.85	56.16**	23.66**	0.64	0.60	0.39**	470.90	65.82**	1.22	0.06	7.45
**MEAN**	**12.31**	**77.86**	**43.93**	**18.52**	**0.72**	**0.56**	**0.29**	**1351.13**	**57.03**	**1.48**	**0.056**	**4.47**
**CD 0.05**	3.32	2.83	6.68	1.21	0.05	0.05	0.05	250.53	2.47	0.08	0.01	0.88
**CD 0.01**	4.41	3.76	8.88	1.61	0.06	0.06	0.07	332.77	3.29	0.11	0.01	1.18

Table 10. Mean performance of parents raised in environment-3

PARENTS	50 DT	50 DS	ASI	CPH	PH	TL	TBR	CL	CG	NKr/R	NR/C	CW
L1	54.00	57.00	3.00	74.83	148.33	31.27	10.66	16.50**	13.75*	29.34**	13.84	104.65*
L2	57.00	59.50	2.50	80.83	162.67**	32.83	14.33**	13.80	11.90	25.84	13.84	94.76
L3	58.00	60.50	2.50	86.33**	170.08**	28.42	9.50	13.68	11.59	27.17	12.00	99.62*
L4	56.50	59.50	3.00	78.67	166.68**	34.42*	13.50	15.67*	12.67	23.67	14.34*	99.73*
L5	54.00	57.50	3.50	63.83	118.17	28.97	9.50	9.84	10.33	23.23	12.67	77.65
L6	51.50**	56.50**	5.00	69.50	133.00	35.05*	12.16	14.28	11.92	28.34*	13.66	100.55*
L7	53.00**	56.00**	3.00	76.50	143.00	28.53	10.17	13.98	13.78*	25.07	12.34	83.67
L8	57.00	59.00	2.00*	69.33	143.00	35.17*	14.34**	16.00**	12.00	26.09	12.00	83.51
L9	54.00	57.00	3.00	74.50	145.08	27.66	10.66	14.36	13.20	25.74	13.50	84.28
**MEAN**	**55.00**	**58.08**	**3.05**	**74.92**	**147.77**	**31.36**	**11.64**	**14.23**	**12.34**	**26.05**	**13.13**	**92.04**
T1	47.00**	51.50**	4.50	63.67	121.17	28.08	18.34**	12.83	11.17*	22.90	11.00	55.69
T2	58.50	61.50	3.00	64.67	125.83	32.42**	15.66**	14.50	8.33	16.82	11.66	47.90
T3	57.00	61.00	4.00	55.83	110.08	25.59	8.34	12.84	8.12	18.91	12.34	60.98
T4	57.00	59.00	2.00**	63.33	136.83	25.75	17.34**	9.84	7.84	15.84	10.66	42.56
T5	56.00	59.00	3.00	54.67	109.83	27.42	12.34	13.49	8.79	23.84**	11.66	55.21
T6	59.00	62.00	3.00	82.67**	164.92**	30.27	14.00	17.25**	13.18**	24.03**	12.00	80.91**
T7	59.00	61.00	2.00**	66.50	142.00	23.70	8.50	13.16	8.78	22.08	10.00	63.54
T8	56.00	64.00	8.00	79.50**	164.17**	31.76*	11.75	14.18	10.24	19.80	12.00	57.10
**MEAN**	**56.18**	**59.87**	**3.68**	**66.35**	**134.35**	**28.12**	**13.28**	**13.50**	**9.55**	**20.52**	**11.41**	**57.98**
**CD 0.05**	1.01	1.12	0.85	8.67	8.65	2.88	1.46	1.15	1.33	2.17	0.86	6.60
**CD 0.01**	1.34	1.49	1.13	11.51	11.49	3.83	1.94	1.52	1.77	2.88	1.04	8.77

Table 10. Cont.,

PARENTS	SHW	SH%	SPY	100 SW	SL	SG	ST	SVI	STR	HIP	TRP	PA
**L1**	23.77**	77.31	80.88*	19.94	0.72	0.73**	0.34	3174.69	71.67	0.85	0.06	11.76
**L2**	16.16	82.94*	78.59	21.99	0.81	0.51	0.29	4153.28	71.07	0.81	0.05	11.37
**L3**	18.52	81.40	81.09*	24.88**	0.73	0.75**	0.39	3955.15	70.18	0.91	0.05	11.06
**L4**	26.83**	73.09	72.89	21.50	0.83	0.70**	0.40*	4138.14	72.62*	0.94	0.08**	11.41
**L5**	12.16	84.28**	65.49	22.25	0.87*	0.65	0.34	3786.33	70.22	0.84	0.07*	10.37
**L6**	16.34	83.75**	84.21**	21.75	0.85	0.75**	0.29	4399.03**	67.16	0.88	0.09**	10.30
**L7**	18.83	77.47	64.83	21.00	0.85	0.42	0.29	4167.94	72.72*	0.81	0.06	10.86
**L8**	15.42	81.51	68.10	21.75	0.80	0.63	0.30	4289.37*	65.09	0.82	0.07*	10.84
**L9**	13.42	84.07**	70.86	20.39	0.84	0.57	0.40*	4272.72	71.66	0.96*	0.08**	9.71**
**MEAN**	**17.93**	**80.64**	**74.10**	**21.71**	**0.81**	**0.63**	**0.33**	**4037.40**	**70.27**	**0.83**	**0.06**	**10.85**
**T1**	12.33	77.87	43.35	17.23	1.00**	0.87**	0.20	1305.27	56.12	1.75**	0.05	2.84**
**T2**	10.99	77.06	36.92	18.82	0.77	0.60	0.27	1876.73**	54.93	1.81**	0.05	2.72**
**T3**	12.25	79.92	48.73	20.91**	0.72	0.53	0.33	556.89	65.36**	1.54	0.05	3.46*
**T4**	8.66	79.63	33.90	20.10	0.65	0.68**	0.33	1044.07	50.77	1.86**	0.05	2.51**
**T5**	9.36	83.08**	45.84	16.50	0.58	0.53	0.31	1915.99**	68.90**	1.13	0.05	5.43
**T6**	20.25**	74.99	60.66**	22.89**	0.73	0.73**	0.32*	1724.39**	70.08**	0.95	0.06*	8.66
**T7**	15.66**	75.35	47.87	19.92	0.75	0.45	0.30	1923.40**	56.39	1.40	0.05	4.00
**T8**	11.32	80.16	45.78	19.27	0.70	0.47	0.33	470.13	66.33**	1.14	0.05	5.50
**MEAN**	**12.60**	**78.50**	**45.38**	**19.45**	**0.73**	**0.60**	**0.29**	**1352.10**	**61.11**	**1.44**	**0.05**	**4.38**
**CD 0.05**	2.18	1.86	6.05	1.09	0.065	0.052	0.03	246.80	1.75	0.13	0.009	0.75
**CD 0.01**	2.89	2.47	8.03	1.45	0.10	0.070	0.05	327.82	2.66	0.17	0.011	0.99

Table 11. Mean performance of parents raised across the environments

PARENTS	50 DT	50 DS	ASI	CPH	PH	TL	TBR	CL	CG	NKr/R	NR/C	CW
**L1**	54.00	57.00	3.00	79.94	156.36*	27.76	10.31	14.17	12.14	24.06	12.56	95.57**
**L2**	57.00	59.83	2.83	83.78**	160.00**	30.17	11.00	13.02	11.45	22.93	13.06	91.88**
**L3**	55.00	57.83	2.83	86.89**	164.36**	28.22	8.50	13.64	11.63	24.47	12.28	92.58**
**L4**	53.83	57.00	3.17	77.95	141.67	31.03	11.56	13.99	11.00	25.03	13.00	80.04
**L5**	52.33**	55.50	3.17	69.83	134.83	31.54*	11.95	12.46	11.05	24.77	12.33	76.56
**L6**	52.50**	56.67	4.17	73.37	134.63	29.01	11.46	14.41	12.07	25.63	13.91**	93.60**
**L7**	51.83**	54.50**	2.67*	78.67	141.33	27.57	13.31*	14.27	11.81	23.52	12.33	77.57
**L8**	54.67	57.67	3.00	72.78	138.06	31.67*	15.00**	15.84**	12.37*	26.25**	11.78	79.52
**L9**	50.67**	54.67**	4.00	70.41	139.52	27.26	10.78	14.43	11.86	26.50**	12.70	86.30
**MEAN**	**53.53**	**56.74**	**3.20**	**77.06**	**145.64**	**29.35**	**11.53**	**14.02**	**11.70**	**24.79**	**12.66**	**85.95**
**T1**	48.00**	51.67**	3.67	75.13	134.03	28.61	14.13*	12.36	9.94	21.33*	11.73	58.49
**T2**	55.50	58.50	3.00**	70.56	128.26	30.50*	11.32	12.16	8.91	19.08	11.93	51.88
**T3**	55.50	59.50	4.00	59.37	109.17	25.64	11.32	11.20	9.09	21.54**	11.83	62.89*
**T4**	54.00	58.00	4.00	64.59	120.57	25.50	11.96	9.98	7.95	16.85	10.14	40.83
**T5**	51.17**	55.17**	4.00	69.28	124.61	28.48	12.82	12.17	9.37	21.98**	12.25*	58.56
**T6**	56.17	59.67	3.50	78.12**	150.03**	28.60	14.23*	14.13**	11.27**	21.73**	12.25*	77.61**
**T7**	52.00**	55.33**	3.33**	76.22*	135.39	25.48	11.33	10.66	8.42	17.84	10.61	50.07
**T8**	52.67*	57.67	5.00	75.90*	142.23**	29.23	12.37	12.83	11.21**	20.17	12.06	65.15
**MEAN**	**53.12**	**56.93**	**3.81**	**71.14**	**130.53**	**27.75**	**12.43**	**11.93**	**9.51**	**20.06**	**11.60**	**58.18**
**CD 0.05**	0.58	0.66	0.48	4.62	8.28	1.93	1.51	0.85	0.67	1.05	0.52	4.11
**CD 0.01**	0.78	0.87	0.63	6.14	11.01	2.56	2.00	1.13	0.90	1.40	0.69	5.46

Table 11. Cont.,

PARENTS	SHW	SH%	SPY	100 SW	SL	SG	ST	SVI	STR	HIP	TRP	PA
**L1**	19.49**	79.70	76.08**	25.90**	0.86**	0.68**	0.42**	3184.68	67.75	0.87	0.06	11.66
**L2**	17.58	80.74	74.30*	24.94**	0.87**	0.57	0.32	4151.41	68.13	0.83	0.06	11.69
**L3**	14.74	84.17**	77.84**	25.99**	0.73	0.69**	0.40**	3943.04	66.85	0.84	0.06	12.06
**L4**	17.15	79.41	62.89	19.42	0.81	0.62	0.33	4127.30	70.56**	0.82	0.08**	11.77
**L5**	14.30	81.31	62.25	20.55	0.82	0.59	0.34	3793.38	70.62**	0.87	0.08**	11.43
**L6**	17.03	81.76	76.57**	21.61	0.84*	0.67**	0.37*	4386.71**	68.73	0.87	0.09**	10.99
**L7**	16.84	78.35	60.73	20.95	0.77	0.52	0.36	4171.13	69.02	0.88	0.06	10.78*
**L8**	16.60	79.08	62.92	20.40	0.71	0.59	0.33	4279.55*	65.70	0.81	0.07	11.31
**L9**	14.33	83.58**	71.97	21.42	0.82	0.70**	0.36	4275.11*	67.70	0.91	0.08**	10.30**
**MEAN**	**16.45**	**80.89**	**69.50**	**22.35**	**0.80**	**0.62**	**0.35**	**4034.69**	**68.33**	**0.85**	**0.07**	**11.33**
**T1**	13.43	77.26	45.06	18.06	0.92**	0.69**	0.22	1307.42	55.96	1.60**	0.05	2.85**
**T2**	12.17	76.81	39.71	17.52	0.76	0.60	0.29	1771.12**	52.23	1.80**	0.05	2.72**
**T3**	14.28	77.48	48.61*	19.21	0.72	0.60	0.32	539.06	59.87	1.48	0.05	3.49**
**T4**	10.18	75.33	30.65	17.91	0.64	0.63*	0.33*	1045.15	50.58	1.86**	0.05	2.52**
**T5**	13.16	77.78	45.40	16.98	0.66	0.56	0.34**	1918.41**	60.78*	1.34	0.06**	4.98
**T6**	17.69**	77.26	59.92**	22.53**	0.79**	0.66**	0.34**	1730.15**	70.52**	1.12	0.06**	8.34
**T7**	13.83	72.03	36.25	17.98	0.72	0.56	0.30	1924.73**	54.05	1.47	0.06**	3.53**
**T8**	14.10	78.43*	51.05**	21.00**	0.69	0.56	0.36**	448.10	64.59**	1.12	0.06**	6.11
**MEAN**	**13.60**	**76.54**	**44.58**	**18.89**	**0.73**	**0.60**	**0.31**	**1335.51**	**58.71**	**1.47**	**0.05**	**4.31**
**CD 0.05**	1.56	1.47	3.84	0.66	0.04	0.03	0.02	185.84	1.39	0.08	0.01	0.40
**CD 0.01**	2.08	1.96	5.10	0.88	0.06	0.04	0.03	246.85	1.85	0.10	0.01	0.53

Table 12. General and Specific Combining ability Variance in Environment-1

	50 DT	50 DS	ASI	CPH	PH	TL	TBR	CL	CG	NKr/R	NR/C	CW
**GCA**	0.0689	0.0739	0.0054	1.2848	4.0214	-0.0718	0.0084	-0.0023	-0.0001	0.0201	0.0055	0.5781
**SCA**	0.2755	5.7548	1.1329	129.7521	316.1049	29.5027	5.9590	1.2802	0.7244	17.2062	1.0315	720.5444
**GCA/SCA**	0.2501	0.0128	0.0048	0.0099	0.0127	-0.0024	0.0014	-0.0018	-0.0001	0.0012	0.0053	0.0008
	**SHW**	**SH%**	**SPY**	**100 SW**	**SL**	**SG**	**ST**	**SVI**	**STR**	**HIP**	**TRP**	**PA**
**GCA**	0.0042	0.0166	0.5681	0.2160	0.0001	0.0001	0.0010	2606.1330	0.2938	-0.0001	0.0000	0.0094
**SCA**	12.9027	8.0344	654.1556	15.2229	0.0091	0.0076	0.0023	307320.5360	38.0518	0.0825	0.0001	2.1442
**GCA/SCA**	0.0003	0.0021	0.0009	0.0142	0.0110	0.0132	0.4348	0.0085	0.0077	-0.0012	0.0100	0.0044

Table 13. General and Specific Combining ability Variance in Environment-2

	50 DT	50 DS	ASI	CPH	PH	TL	TBR	CL	CG	NKr/R	NR/C	CW
**GCA**	0.0295	0.0210	-0.0021	1.1775	4.5780	0.0362	0.0468	0.0148	0.0075	0.0140	0.0062	3.7431
**SCA**	5.0954	4.3328	0.8322	108.5363	361.7898	12.8781	6.5559	3.3593	1.4272	14.5680	0.8728	675.7972
**GCA/SCA**	0.0058	0.0048	-0.0025	0.0108	0.0127	0.0028	0.0071	0.0044	0.0053	0.0010	0.0071	0.0055
	**SHW**	**SH%**	**SPY**	**100 SW**	**SL**	**SG**	**ST**	**SVI**	**STR**	**HIP**	**TRP**	**PA**
**GCA**	0.2160	0.0550	2.7464	0.3543	0.0001	0.0001	0.0001	3758.5095	0.2303	0.0010	0.0001	0.0008
**SCA**	14.6951	6.7838	559.4452	8.3277	0.0044	0.0062	0.0022	369525.6651	39.7902	0.0388	0.0010	3.5720
**GCA/SCA**	0.0147	0.0081	0.0049	0.0425	0.0227	0.0161	0.0455	0.0102	0.0058	0.0258	0.1000	0.0002

Table 14. General and Specific Combining ability Variance in Environment-3

	50 DT	50 DS	ASI	CPH	PH	TL	TBR	CL	CG	NKr/R	NR/C	CW
**GCA**	0.0416	0.0453	-0.0017	1.5492	6.3642	-0.0301	0.0403	0.0092	0.0207	0.0146	-0.0027	5.9505
**SCA**	4.6013	4.0253	0.5734	169.9190	409.5719	22.4165	6.8887	2.7180	0.8554	17.5893	1.1863	616.9318
**GCA/SCA**	0.0090	0.0113	-0.0030	0.0091	0.0155	-0.0013	0.0059	0.0034	0.0242	0.0008	-0.0023	0.0096
	**SHW**	**SH%**	**SPY**	**100 SW**	**SL**	**SG**	**ST**	**SVI**	**STR**	**HIP**	**TRP**	**PA**
**GCA**	0.1749	0.0091	4.3817	0.3055	0.0001	0.0001	0.0010	3811.3276	-0.1250	-0.0001	0.0000	-0.0041
**SCA**	17.6228	6.4963	513.3176	7.1107	0.0060	0.0120	0.0028	372601.9397	38.3783	0.0466	0.0001	2.5389
**GCA/SCA**	0.0099	0.0014	0.0085	0.0430	0.0167	0.0083	0.3571	0.0102	-0.0033	-0.0021	0.1000	-0.0016

Table 15. General and Specific Combining ability Variance across the environments

	50 DT	50 DS	ASI	CPH	PH	TL	TBR	CL	CG	NKr/R	NR/C	CW
**GCA**	0.0384	0.0346	0.0006	0.9258	3.7098	-0.0112	0.0075	0.0058	0.0077	0.0443	0.0037	3.7978
**SCA**	3.9765	3.3876	0.3930	70.8807	184.0500	12.4877	2.5552	0.6862	0.2165	6.5342	0.5136	410.6370
**GCA/SCA**	0.0097	0.0102	0.0015	0.0131	0.0202	-0.0009	0.0029	0.0085	0.0356	0.0068	0.0072	0.0092
	**SHW**	**SH%**	**SPY**	**100 SW**	**SL**	**SG**	**ST**	**SVI**	**STR**	**HIP**	**TRP**	**PA**
**GCA**	0.0929	0.0157	2.9495	0.2683	0.0001	0.0001	0.0001	328.5081	0.1215	-0.0001	0.0001	0.0022
**SCA**	5.3528	2.6819	355.5236	5.5219	0.0022	0.0003	0.0010	323362.1980	13.7734	0.0315	0.0010	1.5911
**GCA/SCA**	0.0174	0.0059	0.0083	0.0486	0.0455	0.3333	0.1000	0.0010	0.0088	-0.0032	0.1000	0.0014

Table 16. Mean performance of hybrids raised in environment-1

Code No	50 DT	50 DS	ASI	CPH	PH	TL	TBR	CL	CG	NKr/R	NR/C	CW	SHW
L1 x T1	52.50	55.50	3.00*	96.50	173.50	26.17	13.00	16.84	12.66	30.66	13.00	121.14	19.14
L1 x T2	49.00	52.00*	3.00*	92.83	167.67	36.50	17.00	16.66	13.00	32.65	14.34	140.18	18.56
L1 xT3	52.00	54.00	2.00**	100.00	165.67	23.27	12.22	16.86	13.04	25.23	14.16	107.88	20.35
L1 x T4	52.50	54.50	2.00**	95.17	182.83	31.83	14.50	15.36	13.50	31.79	13.33	140.70	26.25**
L1 x T5	50.00	54.50	4.50	95.17	188.50*	26.33	18.20*	19.19*	13.00	29.73	12.34	144.85	29.50**
L1 x T6	49.00	52.50	3.50	108.00*	194.67**	35.33	11.00	17.67	13.90	34.77*	14.00	177.08**	23.26
L1 x T7	49.50	52.00*	2.50**	121.37**	206.67**	32.50	14.16	18.43	13.87	31.84	13.00	156.64**	19.58
L1 x T8	52.50	54.50	2.00**	81.83	136.83	37.00*	12.33	13.95	12.66	22.16	15.00	93.69	14.80
L2 x T1	52.00	55.00	3.00*	106.83	217.17**	32.22	17.50*	18.86*	14.25	36.17**	16.16**	187.62**	24.41*
L2 x T2	47.50**	51.50**	4.00	113.50**	203.67**	29.83	15.34	17.50	13.85	33.01	14.34	164.94**	24.41*
L2 x T3	48.00*	52.00*	4.00	92.21	174.67	22.34	7.66	14.35	11.40	32.00	13.00	114.47	19.25
L2 x T4	52.50	56.50	4.00	119.83**	192.50*	28.50	13.50	17.47	13.33	33.00	15.84*	182.61**	22.91
L2 x T5	52.50	56.50	4.00	102.33	192.33*	36.00	14.34	17.53	13.30	32.83	14.00	148.51	23.73
L2 x T6	52.00	54.00	2.00**	132.00**	221.67**	30.17	13.66	16.93	13.01	33.67	13.66	150.16	18.00
L2 x T7	54.00	59.00	5.00	116.00**	200.38**	37.83*	12.67	15.67	13.00	28.85	14.33	157.76**	25.83**
L2 x T8	43.00**	49.00**	6.00	93.67	180.50	40.00**	13.67	16.36	10.09	30.50	14.34	171.21**	24.42*
L3 x T1	53.50	58.50	5.00	115.00**	204.17**	27.66	10.84	16.37	13.95	25.67	14.83	133.02	25.66**
L3 x T2	51.50	55.50	4.00	111.67**	199.33**	32.25	12.84	16.91	13.37	26.88	14.67	134.85	22.48
L3 xT3	52.00	54.00	2.00**	122.17**	215.67**	34.67	12.83	17.48	13.71	29.34	13.66	130.19	18.00
L3 x T4	52.00	56.50	4.50	92.50	168.67	30.00	13.00	17.07	13.73	33.50	15.00	198.14**	26.59*
L3 x T5	48.50	53.50	5.00	89.67	177.83	31.00	17.16	15.59	12.89	33.83	16.34**	120.57	20.41
L3 x T6	54.00	58.00	4.00	105.67	180.83	29.34	11.34	16.41	12.64	23.49	14.34	145.13	24.78*
L3 x T7	51.50	53.50	2.00**	105.73	200.90**	35.25	10.29	16.26	13.93	22.34	14.06	117.69	23.79
L3 x T8	47.50**	52.50	5.00	102.83	199.00**	40.83**	13.66	17.58	13.11	30.84	14.34	170.15**	22.90
L4 x T1	45.00**	52.00*	7.00	96.33	170.67	32.16	13.50	15.63	13.37	31.83	14.00	132.56	22.66
L4 x T2	41.50**	45.50**	4.00	92.50	170.67	31.83	12.67	17.13	11.75	34.83*	14.00	153.67*	15.83
L4 xT3	48.00*	52.00*	4.00	97.83	175.33	34.33	21.66**	15.53	12.00	32.85	15.00	147.64	21.50
L4 x T4	51.50	55.00	3.50	103.17	167.83	33.50	15.34	15.35	13.41	23.60	15.66*	116.56	22.59
L4 x T5	52.50	54.50	2.00**	107.17	180.17	38.00**	15.50	16.89	13.95	27.66	15.00	133.86	23.23
L4 x T6	53.00	58.00	5.00	109.00*	191.50*	32.83	12.84	16.43	13.90	32.65	14.34	167.60**	29.20**
L4 x T7	46.00**	51.00**	5.00	113.50**	164.17	31.34	13.34	19.10*	12.40	44.33**	16.00**	189.33**	20.91
L4 x T8	48.00*	52.00*	4.00	97.08	169.50	34.92	8.91	18.19	13.95	35.31**	15.84*	160.99	29.75**
L5 x T1	50.50	55.50	5.00	94.17	171.50	33.67	12.83	18.98*	14.16	27.17	14.34	120.76	24.95*
L5 x T2	45.00**	49.50**	4.50	88.50	163.94	30.50	16.50	19.98**	13.07	33.94	14.34	128.67	22.73
L5 xT3	45.50**	49.50**	4.00	80.33	134.83	37.83*	12.50	16.72	12.20	34.17	14.00	124.04	16.43
L5 x T4	47.50**	52.00*	4.50	98.33	168.17	46.00**	12.83	15.78	12.10	33.83	15.00	134.88	17.75
L5 x T5	47.50**	52.50	5.00	97.67	166.33	32.50	11.50	16.64	12.97	27.00	14.34	128.17	17.50
L5 x T6	45.50**	50.00**	4.50	110.42*	172.58	32.83	13.59	18.35	15.18**	34.23	15.00	182.18**	29.75**

L5 x T7	54.00	59.00	5.00	79.75	140.00	22.50	17.00	17.50	14.85*	32.55	13.84	138.69	26.07**
L5 x T8	54.00	56.00	2.00**	114.35**	132.51	20.00	15.21	16.21	14.00	31.50	12.00	106.92	25.62**
L6 x T1	52.50	59.00	6.50	81.50	149.57	32.83	12.83	18.70	12.80	36.02**	14.00	106.09	22.50
L6 x T2	45.50**	48.00**	2.50**	72.73	141.17	39.17**	12.34	18.44	12.15	33.72	14.66	88.36	15.42
L6 xT3	47.50**	51.00	3.50	84.67	168.67	41.33**	18.16*	17.84	13.66	32.33	15.16	150.44	23.08
L6 x T4	48.00*	53.00	5.00	78.00	169.67	34.83	12.50	17.25	14.11	39.50**	15.84*	148.30	23.00
L6 x T5	47.00**	51.50**	4.50	96.67	178.50	28.66	13.33	15.40	13.33	31.34	14.00	124.81	21.09
L6 x T6	49.00	53.50	4.50	113.33**	196.33**	34.00	15.83	16.18	13.84	29.00	14.34	148.76	21.75
L6 x T7	47.00**	53.00	6.00	95.67	192.83**	38.17**	16.17	18.90*	14.90*	35.73**	15.84*	178.81**	24.81*
L6 x T8	45.50**	49.00**	3.50	91.17	170.17	37.83*	17.17	16.34	12.84	31.33	16.66**	142.42	20.77
L7 x T1	48.00*	53.00	5.00	100.83	173.17	45.50**	15.66	17.47	14.03	38.58**	18.00**	198.39**	17.87
L7 x T2	50.50	54.00	3.50	106.00	192.17*	32.00	15.00	18.27	13.35	32.67	15.66*	158.36**	17.25
L7 xT3	51.00	53.00	2.00**	96.67	187.83	35.67	12.84	17.47	14.00	32.16	14.34	140.43	20.25
L7 x T4	49.00	53.00	4.00	105.22	183.17	30.59	11.25	15.75	13.74	30.04	13.91	107.19	19.91
L7 x T5	50.00	54.00	4.00	44.50	96.50	12.53	8.40	17.70	11.35	37.00**	17.00**	136.46	20.75
L7 x T6	52.50	55.00	2.50**	114.00**	187.00	33.83	13.00	17.11	14.38	33.02	14.67	156.92**	16.60
L7 x T7	51.50	54.00	2.50**	85.33	169.83	32.50	13.50	17.10	13.39	33.01	14.16	172.53**	18.73
L7 x T8	48.00*	51.00**	3.00*	105.50	179.67	36.33	15.17	18.65	13.59	31.33	16.00**	123.81	18.91
L8 x T1	51.00	53.50	2.50**	110.00	205.00**	35.50	15.34	16.46	13.63	31.23	12.84	120.10	27.16**
L8 x T2	49.00	54.00	5.00	101.83	184.67	27.66	15.00	16.86	13.09	31.66	14.16	145.97	19.05
L8 xT3	46.50**	50.50**	4.00	101.67	168.17	32.83	18.50**	13.55	11.18	34.66*	12.34	176.36**	27.00**
L8 x T4	52.50	56.50	4.00	91.67	165.00	32.83	15.00	15.20	13.36	36.00**	13.00	129.65	19.34
L8 x T5	47.00**	53.00	6.00	89.83	169.33	32.50	16.00	16.70	14.00	39.83**	16.67*	183.40**	23.50
L8 x T6	49.00	53.00	4.00	103.50	179.00	35.83	15.00	19.15*	13.66	37.74**	15.66*	220.71**	28.25**
L8 x T7	47.00**	52.00*	5.00	93.33	171.00	33.00	15.33	17.94	14.40	27.94	13.84	122.40	18.90
L8 x T8	45.00**	48.00**	3.00*	93.17	174.33	37.67*	17.50*	17.40	13.39	36.20**	15.34	154.24*	20.66
L9 x T1	48.50	53.00	4.50	90.17	173.17	37.17*	13.16	16.57	12.93	28.86	16.33**	98.69	16.74
L9 x T2	44.00**	48.50**	4.50	99.67	166.17	37.17*	15.66	16.85	12.39	31.76	16.00**	113.95	20.08
L9 xT3	47.00**	51.00**	4.00	89.50	170.50	40.00**	17.83*	18.77*	15.80**	38.95**	13.66	164.71**	27.18**
L9 x T4	48.00*	53.00	5.00	89.67	156.67	33.16	18.66**	17.98	14.97*	40.04**	15.84*	168.07**	27.10**
L9 x T5	45.00**	48.00**	3.00*	88.00	172.00	29.00	17.50*	17.40	12.18	34.29	15.00	161.75**	23.13
L9 x T6	46.00**	50.00**	4.00	102.50	187.17	35.67	18.16*	15.84	13.02	33.83	13.66	113.10	19.84
L9 x T7	51.00	54.50	3.50	94.83	180.67	39.33**	17.50*	16.80	13.85	35.23**	14.00	152.05	19.34
L9 x T8	48.00*	52.00*	4.00	113.33**	207.67**	33.50	14.00	14.75	13.75	23.49	14.83	117.69	23.25
Overall mean	**49.18**	**53.12**	**3.94**	**98.85**	**177.11**	**33.20**	**14.33**	**17.01**	**13.33**	**32.23**	**14.61**	**144.45**	**22.07**
CD at 5 %	1.03	1.0511	0.8703	8.973	11.7668	3.5635	3.1204	1.7303	1.2509	2.1247	1.0327	8.0503	2.2551
CD at 1 %	1.37	1.40	1.16	11.92	15.63	4.73	4.14	2.30	1.66	2.82	1.37	10.69	3.00

Table 16. Cont.,

	SH%	SPY	100 SW	SL	SG	ST	SVI	STR	HIP	TRP	PA
L1 x T1	84.20	101.99	25.60	0.95	0.80**	0.35	3926.83	63.64	1.48**	0.04	5.22**
L1 x T2	86.76	121.62	26.00	0.84	0.73*	0.30	3556.09	66.46	1.08	0.06	8.47
L1 xT3	81.14	87.53	24.50	0.78	0.70	0.40*	3338.29	63.25	0.80	0.05	9.86
L1 x T4	81.35	130.86	27.00	0.88	0.62	0.33	3460.96	70.12**	1.81**	0.07*	4.45**
L1 x T5	85.08	123.34	33.57**	0.85	0.70	0.31	2987.05	67.19	1.59**	0.06	5.30**
L1 x T6	86.87	153.82**	31.60**	0.80	0.77**	0.30	4495.40*	74.87**	0.89	0.06	9.02
L1 x T7	87.51*	137.05**	33.12**	0.98*	0.73*	0.35	4024.27	72.73**	0.91	0.07*	7.94
L1 x T8	84.19	78.89	23.75	0.90	0.72*	0.33	4888.67**	58.24	0.65	0.05	12.07
L2 x T1	86.99	163.20**	27.92*	0.95	0.77**	0.42*	4922.57**	71.70**	0.82	0.05	9.51
L2 x T2	85.19	140.52**	29.70**	0.85	0.70	0.34	4928.39**	75.89**	0.66	0.07*	10.48
L2 x T3	83.20	95.22	23.00	0.84	0.60	0.25	4106.36	66.70	0.72	0.06	9.05
L2 x T4	87.46*	159.70**	30.57**	0.91	0.78**	0.32	4615.37**	73.97**	0.83	0.05	8.69
L2 x T5	84.02	124.78	27.20	0.84	0.73*	0.40*	4231.86	63.65	0.88	0.05	7.75
L2 x T6	87.99*	132.16*	28.73**	0.99*	0.75**	0.30	4185.61	76.94**	0.88	0.06	8.26
L2 x T7	83.62	131.93*	31.90**	0.80	0.63	0.37	4891.72**	65.47	0.91	0.04	8.41
L2 x T8	85.72	146.79**	33.60**	0.88	0.64	0.33	4109.43	68.67	0.82	0.07*	8.74
L3 x T1	80.71	107.35	31.34**	0.78	0.63	0.37	4167.67	59.36	0.82	0.06	8.10
L3 x T2	83.33	112.37	28.51**	0.86	0.69	0.33	3965.01	61.82	1.17	0.05	5.98**
L3 xT3	86.17	112.19	28.00*	0.77	0.63	0.32	4170.43	75.03*	0.94	0.06	7.61
L3 x T4	86.58	171.56**	34.17**	0.93	0.63	0.30	4232.06	76.97**	0.84	0.07*	8.10
L3 x T5	83.03	100.15	18.12	0.80	0.45	0.39	3961.92	69.82*	0.87	0.06	8.41
L3 x T6	82.92	120.34	35.75**	0.78	0.66	0.35	4118.52	66.72	0.81	0.07*	8.51
L3 x T7	79.79	93.90	29.90**	0.69	0.66	0.42*	4096.11	62.73	1.22*	0.06	7.67
L3 x T8	86.53	147.25**	33.83**	0.88	0.70	0.32	4255.91	67.42	1.11	0.05	7.06*
L4 x T1	82.90	109.89	24.66	0.85	0.62	0.35	4071.62	57.77	0.97	0.04	8.22
L4 x T2	93.08**	142.83**	28.25**	0.85	0.65	0.30	4475.15*	66.52	0.85	0.08**	8.23
L4 xT3	89.05**	131.14*	25.57	0.73	0.64	0.44*	4291.90	69.20	0.88	0.06	7.54
L4 x T4	80.63	93.98	25.46	0.74	0.59	0.35	3417.22	51.69	0.92	0.08**	7.81
L4 x T5	82.65	110.63	26.70	0.85	0.47	0.34	3952.17	67.04	1.15	0.05	7.60
L4 x T6	82.58	138.40**	29.59**	1.03**	0.93**	0.40*	4593.32**	73.41**	1.29**	0.09**	7.82
L4 x T7	88.95**	168.42**	23.75	0.90	0.56	0.33	4725.22**	75.87**	0.84	0.05	9.78
L4 x T8	81.51	131.24*	23.51	1.00*	0.56	0.26	4173.86	69.40	0.90	0.07*	8.39
L5 x T1	79.34	95.81	24.65	0.91	0.53	0.30	3591.74	59.67	1.67**	0.06	6.49**
L5 x T2	82.32	105.94	21.78	0.75	0.51	0.33	3208.95	65.26	0.82	0.06	8.09
L5 xT3	86.77	107.62	22.54	0.88	0.65	0.42*	3091.39	47.89	0.92	0.06	7.23
L5 x T4	86.83	117.13	23.08	0.94	0.62	0.39	3132.36	54.81	0.91	0.09**	7.93

L5 x T5	86.32	110.67	28.64**	1.01**	0.66	0.37	3996.08	67.38	0.76	0.06	9.24
L5 x T6	83.69	152.43**	29.70**	0.99*	0.77**	0.47*	4724.93**	74.72**	1.40**	0.06	7.58
L5 x T7	81.20	112.61	25.00	0.92	0.70	0.38	4916.92**	65.30	0.88	0.05	8.75
L5 x T8	76.03	81.30	21.53	0.70	0.50	0.30	3262.89	62.24	0.62	0.05	12.78
L6 x T1	78.81	83.60	16.60	0.73	0.43	0.30	3016.99	61.63	0.72	0.09**	8.87
L6 x T2	82.58	72.94	15.73	0.73	0.53	0.32	3489.15	52.00	1.81**	0.05	5.03**
L6 xT3	84.65	127.36	25.97	0.85	0.67	0.33	4366.04	72.77**	0.81	0.09**	6.56**
L6 x T4	84.48	125.31	20.03	1.04**	0.69	0.30	4911.11**	71.95**	0.91	0.07*	8.12
L6 x T5	83.11	103.72	23.65	0.62	0.55	0.32	3997.96	71.47**	0.91	0.08**	8.20
L6 x T6	85.38	127.00	30.57**	0.98*	0.86**	0.35	4212.35	68.05	0.90	0.06	8.51
L6 x T7	86.12	154.00**	27.23	1.02**	0.65	0.42*	4615.06**	69.30	0.84	0.06	8.36
L6 x T8	85.41	121.65	23.30	0.82	0.65	0.43*	4930.49**	71.16**	0.94	0.09**	7.79
L7 x T1	90.99**	180.51**	25.99	0.94	0.69	0.50*	4948.16**	74.87**	0.82	0.06	8.14
L7 x T2	89.09**	141.10**	27.57	1.09**	0.72*	0.39	5121.43**	71.77**	0.77	0.06	8.87
L7 xT3	85.58	120.19	26.08	0.73	0.51	0.37	4621.72**	65.80	1.63**	0.07*	5.27**
L7 x T4	81.41	87.28	20.90	0.75	0.45	0.30	3495.45	63.74	0.85	0.08**	7.52
L7 x T5	84.79	115.71	18.43	0.76	0.66	0.42*	4740.97**	73.33**	0.69	0.06	11.14
L7 x T6	89.42**	140.32**	28.98**	1.12**	0.73*	0.40*	5191.44**	76.00**	1.11	0.05	8.39
L7 x T7	89.14**	153.79**	32.89**	0.86	0.70	0.40*	5069.65**	77.56**	0.96	0.06	8.15
L7 x T8	84.72	104.90	20.98	0.78	0.81**	0.32	4213.18	61.26	1.44**	0.04	6.14**
L8 x T1	77.38	92.93	23.20	0.80	0.59	0.34	3121.11	55.99	1.61**	0.07*	5.58**
L8 x T2	86.95	126.93	28.30**	0.90	0.69	0.32	4167.27	67.92	0.76	0.07*	9.40
L8 xT3	84.67	149.36**	34.95**	0.95	0.62	0.35	4989.99**	74.78**	0.94	0.06	7.97
L8 x T4	85.09	110.31	23.57	0.80	0.56	0.35	3911.74	61.69	0.90	0.06	8.46
L8 x T5	87.17	159.90**	24.09	0.90	0.62	0.37	5218.32**	73.18**	1.37**	0.06	7.41
L8 x T6	87.20	192.46**	32.57**	1.00*	0.74*	0.45*	5525.25**	77.24**	0.97	0.09**	8.72
L8 x T7	84.55	103.51	26.78	0.88	0.68	0.35	4503.90*	69.67*	0.85	0.04	8.36
L8 x T8	86.60	133.58**	24.08	0.84	0.65	0.30	4621.92**	68.27	0.75	0.07*	9.91
L9 x T1	82.98	81.95	17.39	0.60	0.45	0.30	3837.14	55.02	1.51**	0.06	4.98**
L9 x T2	82.36	93.87	18.47	0.77	0.48	0.32	3612.17	59.74	1.40**	0.05	5.75**
L9 xT3	83.50	137.54**	25.85	0.84	0.69	0.45*	4709.84**	59.17	1.33**	0.05	5.19**
L9 x T4	83.87	140.97**	22.23	0.97*	0.72*	0.40*	4930.83**	62.85	1.38**	0.05	6.79**
L9 x T5	85.71	138.62**	26.96	0.80	0.64	0.32	4619.98**	70.53**	0.92	0.07*	8.39
L9 x T6	82.48	93.27	20.18	0.78	0.59	0.30	3914.45	64.36	1.06	0.05	5.78**
L9 x T7	87.28	132.72*	26.90	0.80	0.65	0.32	4596.45*	75.38**	1.27*	0.05	6.38**
L9 x T8	80.22	94.44	27.14	0.75	0.47	0.39	3214.90	70.52**	0.88	0.05	7.48
Overall mean	**84.55**	**122.64**	**26.24**	**0.86**	**0.65**	**0.35**	**4218.04**	**67.17**	**1.02**	**0.06**	**7.91**
CD at 5 %	2.7626	7.613	1.3868	0.1132	0.0733	0.0538	225.1596	2.2568	0.1661	0.0096	0.7277
CD at 1 %	3.67	10.11	1.84	0.15	0.10	0.07	299.08	3.00	0.22	0.01	0.97

Table 17. *sca* of the 72 hybrids in Environment 1

Code No	50 DT	50 DS	ASI	CPH	PH	TL	TBR	CL	CG	NKr/R	NR/C	CW	SHW
L1 x T1	0.42	-0.07	-0.49	-2.55	-8.42 *	-5.40 **	-0.57	-0.35	-0.74	1.24	-0.86 *	-5.05	-2.57 **
L1 x T2	0.25	0.49	0.24	-4.87	-8.87 *	5.59 **	2.57 **	-0.82	0.23	2.68 **	0.62	12.82 **	-0.34
L1 xT3	1.69 **	1.54 **	-0.15	3.87	-7.75	-8.24 **	-2.41 *	0.49	0.16	-4.81 **	1.20 **	-22.52 **	-0.46
L1 x T4	0.42	-0.51	-0.93 **	-1.91	10.18 *	0.44	0.71	-0.86	0.04	0.69	-0.53	2.54	4.06 **
L1 x T5	-0.58	0.82 *	1.40 **	5.04	19.51 **	-1.20	3.82 **	2.32 **	0.13	-0.51	-1.66 **	11.54 **	7.60 **
L1 x T6	-2.69 **	-1.85 **	0.85 **	-2.95	4.65	4.10 **	-2.55 **	0.69	0.29	4.66 **	0.56	23.86 **	0.40
L1 x T7	-2.36 **	-2.79 **	-0.43	20.75 **	26.02 **	0.98	0.00	1.05	0.15	1.79 *	-0.37	11.83 **	-1.78 *
L1 x T8	2.86 **	2.38 **	-0.49	-17.40 **	-35.34 **	3.74 **	-1.57	-2.52 **	-0.25	-5.75 **	1.04 *	-35.03 **	-6.90 **
L2 x T1	0.60	-1.07 **	-1.67 **	-2.91	14.43 **	-0.35	4.44 **	1.72 *	1.27 **	4.10 **	1.49 **	37.04 **	1.26
L2 x T2	-0.56	-0.51	0.05	5.11	-1.25	-2.07	1.42	0.05	1.51 **	0.39	-0.20	13.18 **	4.07 **
L2 x T3	-1.62 **	-0.96 **	0.66 *	-14.61 **	-19.56 **	-10.16 **	-6.46 **	-1.99 **	-1.05 *	-0.68	-0.77	-40.31 **	-3.00 **
L2 x T4	1.10 **	0.99 **	-0.12	12.07 **	-0.97	-3.88 **	0.22	1.28	0.29	-0.75	1.17 **	20.06 **	-0.72
L2 x T5	2.60 **	2.32 **	-0.28	1.52	2.53	7.47 **	0.46	0.70	0.85	-0.05	-0.81 *	-9.18 **	0.39
L2 x T6	0.99 **	-0.85 *	-1.84 **	10.37 **	10.84 **	-2.06	0.63	-0.02	-0.16	0.91	-0.59	-27.45 **	-6.30 **
L2 x T7	2.83 **	3.71 **	0.88 **	4.69	-1.09	5.32 **	-0.98	-1.68 *	-0.30	-3.84 **	0.14	-11.44 **	3.03 **
L2 x T8	-5.95 **	-3.63 **	2.33 **	-16.25 **	-12.49 **	5.75 **	0.28	-0.07	-2.41 **	-0.07	-0.44	18.10 **	1.27
L3 x T1	0.98 **	1.37 **	0.39	9.16 **	5.99	-5.42 **	-1.43	-0.65	0.33	-2.13 **	-0.04	-1.62	2.31 **
L3 x T2	2.31 **	2.42 **	0.11	7.17 *	6.54	-0.17	-0.28	-0.41	0.39	-1.47	-0.06	-0.96	1.93 *
L3 xT3	1.26 **	-0.02	-1.28 **	19.25 **	26.00 **	1.65	-0.50	1.27	0.62	0.92	-0.30	-8.64 **	-4.46 **
L3 x T4	-0.52	-0.08	0.44	-11.37 **	-20.25 **	-2.90 *	0.52	1.01	0.06	4.02 **	0.14	51.53 **	2.75 **
L3 x T5	-2.52 **	-1.74 **	0.78 *	-7.25 *	-7.41	1.96	4.09 **	-1.12	-0.20	5.22 **	1.33 **	-21.18 **	-3.14 **
L3 x T6	1.87 **	2.09 **	0.22	-12.07 **	-25.44 **	-3.41 *	-0.90	-0.41	-1.18 **	-5.00 **	-0.11	-16.54 **	0.28
L3 x T7	-0.80 *	-2.85 **	-2.06 **	-1.69	4.00	2.22	-2.56 **	-0.96	0.00	-6.09 **	-0.32	-35.57 **	0.78
L3 x T8	-2.58 **	-1.19 **	1.39 **	-3.19	10.57 *	6.07 **	1.07	1.27	-0.02	4.54 **	-0.63	32.98 **	-0.46
L4 x T1	-4.40 **	-2.38 **	2.01 **	-5.93	-7.94	-1.91	-0.24	-1.46 *	0.07	-0.62	-1.20 **	-8.64 **	-0.82
L4 x T2	-4.56 **	-4.83 **	-0.26	-8.42 *	-2.55	-1.57	-1.92 *	-0.27	-0.91 *	1.84 *	-1.05 **	11.29 **	-4.85 **
L4 xT3	0.38	0.73 *	0.35	-1.51	5.24	0.33	6.86 **	-0.75	-0.77	-0.21	0.71	2.24	-1.09
L4 x T4	2.10 **	1.17 **	-0.93 **	2.88	-1.51	-0.39	1.38	-0.78	0.07	-10.53 **	0.48	-36.61 **	-1.38
L4 x T5	4.60 **	2.01 **	-2.60 **	13.83 **	14.50 **	7.97 **	0.95	0.11	1.19 **	-5.60 **	-0.33	-14.45 **	-0.45
L4 x T6	3.99 **	4.84 **	0.85 **	-5.16	4.80	-0.90	-0.88	-0.46	0.41	-0.49	-0.44	-0.63	4.56 **
L4 x T7	-3.17 **	-2.60 **	0.57	9.66 **	-13.17 **	-2.68 *	-0.99	1.80 **	-1.21 **	11.26 **	1.29 **	29.53 **	-2.23 *
L4 x T8	1.05 **	1.06 **	0.01	-5.36	0.64	-0.85	-5.16 **	1.81 **	1.14 *	4.36 **	0.54	17.26 **	6.26 **
L5 x T1	0.60	0.62	0.01	-1.46	10.39 *	1.23	-0.69	1.15	0.39	-4.20 **	0.01	-3.20	2.07 *
L5 x T2	-1.56 **	-1.33 **	0.24	-5.78	8.21 *	-1.27	2.13 *	1.84 **	-0.06	2.03 *	0.16	3.53	2.66 **
L5 xT3	-2.62 **	-2.27 **	0.35	-12.37 **	-17.77 **	5.47 **	-2.08 *	-0.30	-1.04 *	2.19 **	0.58	-4.12	-5.56 **
L5 x T4	-2.40 **	-2.33 **	0.07	4.68	16.32 **	13.75 **	-0.90	-1.08	-1.72 **	0.79	0.68	-1.05	-5.61 **
L5 x T5	-0.90 *	-0.49	0.40	10.96 **	18.16 **	4.10 **	-2.82 **	-0.88	-0.27	-5.18 **	-0.12	-2.90	-5.57 **

L5 x T6	-4.01 **	-3.66 **	0.35	2.89	3.38	0.74	0.10	0.72	1.22 **	2.17 **	1.10 **	31.19 **	5.72 **
L5 x T7	4.33 **	4.90 **	0.57	-17.45 **	-19.84 **	-9.88 **	2.90 **	-0.53	0.77	0.56	0.00	-3.88	3.55 **
L5 x T8	6.55 **	4.56 **	-1.99 **	18.54 **	-18.85 **	-14.12 **	1.36	-0.90	0.72	1.64 *	-2.42 **	-19.57 **	2.74 **
L6 x T1	3.54 **	4.87 **	1.33 **	-7.90 *	-26.17 **	-3.48 *	-1.48	1.01	-0.86	2.83 **	-1.28 **	-20.83 **	0.67
L6 x T2	-0.12	-2.08 **	-1.95 **	-15.33 **	-29.19 **	3.52 *	-2.83 **	0.44	-0.87	-0.01	-0.47	-39.74 **	-3.60 **
L6 xT3	0.32	-0.02	-0.34	-1.82	1.43	5.09 **	2.79 **	0.96	0.54	-1.46	0.79 *	19.32 **	2.15 *
L6 x T4	-0.96 **	-0.58	0.38	-9.43 **	3.20	-1.29	-2.03 *	0.52	0.40	4.63 **	0.56	9.41 **	0.69
L6 x T5	-0.46	-0.74 *	-0.28	16.19 **	15.69 **	-3.61 **	-1.79	-1.98 **	0.21	-2.67 **	-1.41 **	-9.23 **	-0.94
L6 x T6	0.43	0.59	0.16	12.03 **	12.49 **	-1.97	1.54	-1.31	-0.02	-4.88 **	-0.52	-5.19	-1.23
L6 x T7	-1.74 **	-0.35	1.38 **	4.68	18.37 **	1.91	1.27	1.01	0.93 *	1.91 *	1.05 *	33.27 **	3.33 **
L6 x T8	-1.01 **	-1.69 **	-0.67 *	1.59	4.17	-0.16	2.53 **	-0.64	-0.33	-0.35	1.29 **	12.98 **	-1.06
L7 x T1	-3.27 **	-2.26 **	1.01 **	5.89	-2.88	12.68 **	3.04 **	-0.29	0.35	5.54 **	2.31 **	58.20 **	-1.20
L7 x T2	2.56 **	2.80 **	0.24	12.40 **	21.51 **	-0.16	1.52	0.22	0.31	-0.93	0.12	17.00 **	1.00
L7 xT3	1.51 **	0.85 *	-0.65 *	4.64	20.30 **	2.91 *	-0.85	0.53	0.85	-1.49	-0.45	-3.95	2.08 *
L7 x T4	-2.27 **	-1.70 **	0.57	12.25 **	16.39 **	-2.06	-1.59	-1.04	0.00	-4.68 **	-1.77 **	-44.96 **	0.37
L7 x T5	0.23	0.63	0.40	-41.52 **	-66.61 **	-16.26 **	-5.03 **	0.27	-1.80 **	3.14 **	1.18 **	-10.84 **	1.49
L7 x T6	1.62 **	0.97 **	-0.65 *	7.15 *	2.86	1.34	0.40	-0.44	0.51	-0.71	-0.59	-10.29 **	-3.61 **
L7 x T7	0.45	-0.48	-0.93 **	-11.19 **	-4.94	-0.27	0.29	-0.85	-0.61	-0.65	-1.03 *	13.73 **	0.02
L7 x T8	-0.83 *	-0.81 *	0.01	10.38 **	13.37 **	1.82	2.22 *	1.62 *	0.39	-0.21	0.22	-18.90 **	-0.15
L8 x T1	1.42 **	-0.94 **	-2.36 **	11.69 **	23.06 **	1.56	-0.14	-0.51	0.08	-2.75 **	-1.61 **	-27.43 **	3.90 **
L8 x T2	2.75 **	3.61 **	0.86 **	4.87	8.11 *	-5.61 **	-1.33	-0.41	0.18	-2.86 **	-0.14	-2.73	-1.40
L8 xT3	-1.31 **	-0.83 *	0.47	6.27	-5.27	-1.03	1.96 *	-2.61 **	-1.83 **	0.07	-1.21 **	24.64 **	4.63 **
L8 x T4	2.92 **	2.61 **	-0.31	-4.68	-7.67	-0.92	-0.69	-0.81	-0.23	0.35	-1.44 **	-29.85 **	-4.41 **
L8 x T5	-1.08 **	0.44	1.53 **	0.45	0.32	2.61	-0.29	0.05	0.99 *	5.04 **	2.09 **	28.77 **	0.04
L8 x T6	-0.19	-0.22	-0.03	-6.71 *	-11.03 **	2.23	-0.45	2.38 **	-0.07	3.07 **	1.64 **	46.15 **	3.84 **
L8 x T7	-2.36 **	-1.67 **	0.69 *	-6.56 *	-9.67 *	-0.88	-0.74	0.76	0.54	-6.66 **	-0.12	-43.74 **	-4.01 **
L8 x T8	-2.14 **	-3.00 **	-0.86 **	-5.32	2.14	2.04	1.69	1.15	0.33	3.73 **	0.79 *	4.19	-2.60 **
L9 x T1	0.10	-0.13	-0.24	-5.98	-8.46 *	1.09	-2.92 **	-0.61	-0.89 *	-4.01 **	1.19 **	-28.48 **	-5.62 **
L9 x T2	-1.06 **	-0.58	0.49	4.86	-10.08 *	1.75	-1.27	-0.64	-0.79	-1.66 *	1.01 *	-14.40 **	0.53
L9 xT3	0.38	0.98 **	0.60	-3.73	-2.62	3.99 **	0.69	2.40 **	2.52 **	5.47 **	-0.56	33.34 **	5.71 **
L9 x T4	-0.40	0.42	0.82 *	-4.50	-15.70 **	-2.74 *	2.37 *	1.76 *	1.10 *	5.49 **	0.71	28.92 **	4.26 **
L9 x T5	-1.90 **	-3.24 **	-1.35 **	0.78	3.31	-3.04 *	0.61	0.53	-1.10 *	0.60	-0.27	27.46 **	0.58
L9 x T6	-2.01 **	-1.91 **	0.10	-5.55	-2.55	-0.07	2.11 *	-1.15	-0.99 *	0.27	-1.05 *	-41.10 **	-3.67 **
L9 x T7	2.83 **	2.15 **	-0.68 *	-2.89	0.31	3.30 *	0.83	-0.59	-0.28	1.73 *	-0.64	6.26 *	-2.68 **
L9 x T8	2.05 **	2.31 **	0.26	17.01 **	35.79 **	-4.27 **	-2.41 *	-1.72 *	0.43	-7.88 **	-0.40	-12.01 **	0.89

Table 17. Cont.,

	SH%	SPY	100 SW	SL	SG	ST	SVI	STR	HIP	TRP	PA
L1 x T1	1.42	-3.23	-0.45	0.10 *	0.11 **	0.01	354.19 **	1.56	0.19 **	-0.01 **	-1.89 **
L1 x T2	0.94	11.85 **	-0.83	-0.03	0.03	-0.01	-118.74	1.30	-0.08	0.00	0.78 **
L1 xT3	-3.92 **	-23.36 **	-3.67 **	-0.05	-0.01	0.05 **	-465.70 **	-2.70 **	-0.33 **	-0.01 **	2.62 **
L1 x T4	-2.92 **	-2.27	-0.13	-0.01	-0.09 **	0.01	-167.60 *	4.92 **	0.64 **	0.01 **	-2.96 **
L1 x T5	0.34	10.31 **	6.40 **	0.01	0.01	-0.03	-819.20 **	-1.98 *	0.44 **	0.00	-2.74 **
L1 x T6	1.39	22.71 **	-0.04	-0.15 **	-0.06 *	-0.05 **	327.48 **	2.50 **	-0.28 **	-0.00	1.08 **
L1 x T7	2.08	12.86 **	2.61 **	0.09 *	-0.01	-0.00	-196.75 *	2.40 **	-0.19 **	0.02 **	-0.13
L1 x T8	0.67	-28.87 **	-3.90 **	0.05	0.01	0.02	1086.31 **	-8.01 **	-0.38 **	-0.01 *	3.26 **
L2 x T1	3.32 **	36.03 **	0.93	0.09 *	0.10 **	0.07 **	685.71 **	6.32 **	-0.13 *	-0.01	1.32 **
L2 x T2	-1.52	8.80 **	1.94 **	-0.02	0.01	0.02	589.33 **	7.43 **	-0.18 **	0.02 **	1.73 **
L2 x T3	-2.74 *	-37.61 **	-6.11 **	-0.01	-0.09 **	-0.11 **	-361.85 **	-2.56 **	-0.07	0.00	0.74 **
L2 x T4	2.30 *	21.03 **	2.51 **	0.01	0.10 **	-0.01	322.59 **	5.45 **	-0.01	-0.01 **	0.21
L2 x T5	-1.60	-10.21 **	-0.90	-0.01	0.07 **	0.05 **	-238.60 **	-8.84 **	0.06	-0.01 *	-1.36 **
L2 x T6	1.63	-20.90 **	-3.85 **	0.02	-0.06 *	-0.06 **	-646.51 **	1.27	0.05	-0.00	-0.76 **
L2 x T7	-2.70 *	-14.22 **	0.46	-0.10 *	-0.09 **	0.01	6.47	-8.18 **	0.15 *	-0.01	-0.74 **
L2 x T8	1.31	17.08 **	5.02 **	0.02	-0.05 *	0.01	-357.14 **	-0.88	0.12	0.02 **	-1.14 **
L3 x T1	-1.07	-3.67	3.47 **	-0.01	0.03	0.01	308.77 **	-3.13 **	-0.29 **	0.01	1.10 **
L3 x T2	-1.50	-3.20	-0.13	0.06	0.07 *	0.01	3.91	-3.75 **	0.18 **	-0.01 **	-1.60 **
L3 xT3	2.12 *	-4.49	-1.98 **	-0.00	0.01	-0.05 **	80.19	8.65 **	-0.01	0.00	0.48
L3 x T4	3.31 **	49.04 **	5.23 **	0.09 *	0.02	-0.03	317.24 **	11.35 **	-0.15 *	0.00	0.79 **
L3 x T5	-0.70	-18.68 **	-10.85 **	0.02	-0.15 **	0.03	-130.59	0.22	-0.10	0.00	0.48
L3 x T6	-1.55	-16.57 **	2.30 **	-0.11 **	-0.08 **	-0.02	-335.65 **	-6.07 **	-0.17 **	0.01	0.67 *
L3 x T7	-4.64 **	-36.10 **	-2.41 **	-0.14 **	0.01	0.06 **	-411.17 **	-8.03 **	0.30 **	0.01	-0.30
L3 x T8	4.02 **	33.68 **	4.38 **	0.09 *	0.08 **	-0.01	167.30 *	0.76	0.25 **	-0.01 **	-1.63 **
L4 x T1	-0.42	-8.81 **	0.82	0.00	0.03	-0.00	121.12	-3.60 **	-0.14 *	-0.02 **	0.73 **
L4 x T2	6.73 **	19.59 **	3.63 **	-0.01	0.04	-0.02	422.45 **	2.07 *	-0.14 *	0.01 **	0.16
L4 xT3	3.46 **	6.77 *	-0.40	-0.10 *	0.02	0.08 **	110.05	3.94 **	-0.08	-0.00	-0.08
L4 x T4	-4.17 **	-36.22 **	0.54	-0.15 **	-0.02	0.02	-589.20 **	-12.81 **	-0.08	0.01 *	0.01
L4 x T5	-2.62 *	-15.89 **	1.75 **	0.01	-0.12 **	-0.02	-231.94 **	-1.43	0.18 **	-0.02 **	-0.82 **
L4 x T6	-3.42 **	-6.20 *	0.15	0.08	0.19 **	0.04 *	47.55	1.74 *	0.29 **	0.02 **	-0.51
L4 x T7	2.99 **	30.75 **	-4.55 **	0.02	-0.08 **	-0.03	126.33	6.24 **	-0.08	-0.00	1.32 **
L4 x T8	-2.54 *	10.00 **	-1.93 **	0.15 **	-0.05 *	-0.06 **	-6.35	3.85 **	0.04	0.01	-0.80 **
L5 x T1	-1.62	-5.02	2.13 **	0.05	-0.05 *	-0.08 **	113.14	2.50 **	0.53 **	0.01	-1.34 **
L5 x T2	-1.68	0.57	-1.51 **	-0.13 **	-0.09 **	-0.01	-371.85 **	5.01 **	-0.19 **	-0.00	-0.32
L5 xT3	3.54 **	1.13	-2.11 **	0.04	0.04	0.03	-618.56 **	-13.16 **	-0.05	-0.00	-0.73 **
L5 x T4	4.38 **	4.81	-0.52	0.02	0.02	0.03	-402.16 **	-5.49 **	-0.11	0.02 **	-0.21

L5 x T5	3.41 **	2.03	5.00 **	0.16 **	0.08 **	-0.01	283.87 **	3.11 **	-0.24 **	-0.00	0.48
L5 x T6	0.03	25.72 **	1.59 **	0.01	0.04	0.08 **	651.05 **	7.26 **	0.39 **	-0.00	-1.09 **
L5 x T7	-2.41 *	-7.18 *	-1.98 **	0.02	0.06 *	-0.01	789.93 **	-0.13	-0.07	-0.00	-0.05
L5 x T8	-5.67 **	-22.06 **	-2.59 **	-0.17 **	-0.11 **	-0.05 *	-445.43 **	0.90	-0.26 **	-0.01 **	3.25 **
L6 x T1	-3.15 **	-21.24 **	-4.19 **	-0.09 *	-0.16 **	-0.05 **	-913.35 **	-0.67	-0.40 **	0.01 **	1.87 **
L6 x T2	-2.42 *	-36.44 **	-5.84 **	-0.10 *	-0.09 **	-0.00	-543.39 **	-13.38 **	0.81 **	-0.02 **	-2.55 **
L6 xT3	0.42	16.87 **	3.05 **	0.04	0.05 *	-0.03	204.35 **	6.59 **	-0.14 *	0.02 **	-0.58 *
L6 x T4	1.03	8.97 **	-1.84 **	0.16 **	0.07 **	-0.03	924.85 **	6.53 **	-0.09	-0.01 **	0.81 **
L6 x T5	-0.81	-8.93 **	1.75 **	-0.20 **	-0.04	-0.04	-165.99 *	2.06 *	-0.07	0.01 *	0.28
L6 x T6	0.73	-3.72	4.19 **	0.05	0.13 **	-0.01	-313.26 **	-4.55 **	-0.10	-0.02 **	0.68 *
L6 x T7	1.50	30.20 **	1.97 **	0.16 **	0.00	0.05 **	36.34	-1.26	-0.08	-0.01	0.39
L6 x T8	2.71 *	14.28 **	0.91	-0.01	0.03	0.11 **	770.44 **	4.69 **	0.08	0.01 **	-0.90 **
L7 x T1	5.96 **	59.65 **	2.85 **	0.08 *	0.06 *	0.11 **	534.97 **	9.32 **	-0.35 **	0.00	0.87 **
L7 x T2	1.01	15.69 **	3.66 **	0.22 **	0.07 **	0.03	606.04 **	3.14 **	-0.28 **	-0.00	1.02 **
L7 xT3	-1.73	-6.34 *	0.82	-0.10 **	-0.13 **	-0.04 *	-22.82	-3.63 **	0.62 **	0.01 **	-2.14 **
L7 x T4	-5.12 **	-45.08 **	-3.31 **	-0.15 **	-0.19 **	-0.07 **	-973.66 **	-4.93 **	-0.20 **	0.01 **	-0.07
L7 x T5	-2.20 *	-12.96 **	-5.82 **	-0.09 *	0.04	0.03	94.16	0.67	-0.34 **	0.00	2.94 **
L7 x T6	1.69	-6.43 *	0.25	0.15 **	-0.04	-0.00	182.97 *	0.15	0.05	-0.01 **	0.29
L7 x T7	1.45	13.96 **	5.29 **	-0.03	0.02	-0.00	8.07	3.74 **	-0.02	0.01 *	-0.09
L7 x T8	-1.06	-18.50 **	-3.76 **	-0.08	0.17 **	-0.05 *	-429.73 **	-8.46 **	0.52 **	-0.02 **	-2.82 **
L8 x T1	-5.72 **	-31.08 **	-1.90 **	-0.06	-0.02	-0.02	-1124.28 **	-7.61 **	0.45 **	0.00	-1.97 **
L8 x T2	0.81	-1.62	2.42 **	0.03	0.05 *	-0.01	-180.31 *	1.23	-0.27 **	0.01 *	1.27 **
L8 xT3	-0.69	19.70 **	7.73 **	0.11 **	-0.01	-0.02	513.27 **	7.30 **	-0.06	-0.01 *	0.29
L8 x T4	0.50	-25.20 **	-2.60 **	-0.11 **	-0.06 *	0.01	-389.56 **	-5.04 **	-0.14 *	-0.01 **	0.60 *
L8 x T5	2.12 *	28.08 **	-2.13 **	0.05	0.01	0.01	739.33 **	2.47 **	0.35 **	-0.00	-1.06 **
L8 x T6	1.41	42.56 **	1.88 **	0.03	-0.02	0.08 **	684.60 **	3.34 **	-0.07	0.02 **	0.35
L8 x T7	-1.20	-39.47 **	-2.78 **	-0.01	0.02	-0.02	-389.87 **	-2.20 **	-0.11	-0.02 **	-0.16
L8 x T8	2.77 *	7.03 *	-2.62 **	-0.03	0.02	-0.03	146.82	0.50	-0.15 *	0.01 **	0.67 *
L9 x T1	1.29	-22.61 **	-3.66 **	-0.17 **	-0.10 **	-0.05 **	-80.27	-4.69 **	0.15 *	0.01 *	-0.69 *
L9 x T2	-2.37 *	-15.24 **	-3.35 **	-0.01	-0.09 **	-0.01	-407.44 **	-3.05 **	0.17 *	-0.00	-0.49
L9 xT3	-0.46	27.32 **	2.67 **	0.09 *	0.11 **	0.09 **	561.07 **	-4.42 **	0.13 *	-0.01	-0.61 *
L9 x T4	0.68	24.91 **	0.11	0.15 **	0.15 **	0.07 **	957.50 **	0.02	0.14 *	-0.01 *	0.82 **
L9 x T5	2.06	26.25 **	4.79 **	0.05	0.09 **	-0.04 *	468.96 **	3.72 **	-0.29 **	0.01 **	1.81 **
L9 x T6	-1.91	-37.18 **	-6.46 **	-0.09 *	-0.11 **	-0.06 **	-598.23 **	-5.64 **	-0.18 **	-0.01 **	-0.71 **
L9 x T7	2.93 **	9.19 **	1.40 **	-0.00	0.05	-0.05 **	30.65	7.42 **	0.10	0.01 *	-0.25
L9 x T8	-2.21 *	-12.65 **	4.50 **	-0.02	-0.10 **	0.06 **	-932.23 **	6.64 **	-0.23 **	-0.00	0.12

Table 18. Mean performance of hybrids raised in environment-2

Code No	50 DT	50 DS	ASI	CPH	PH	TL	TBR	CL	CG	NKr/R	NR/C	CW	SHW
L1 x T1	52.00	56.00	4.00	81.58**	158.67	30.67	17.10**	16.42	13.29	31.18	12.50	124.55	20.37
L1 x T2	51.00	54.00	3.00	87.67**	162.84	31.75	18.84**	17.67**	14.00**	34.72**	14.16	146.85**	24.12**
L1 xT3	52.00	54.00	2.00**	90.67**	165.23	30.56	18.23**	15.84	11.96	27.92	13.84	118.15	22.88*
L1 x T4	51.00	55.00	4.00	79.83**	165.50	32.33	18.67**	17.33*	14.67**	32.20	13.67	141.20	26.62**
L1 x T5	52.00	56.00	4.00	93.13**	168.63*	34.96**	18.75**	16.77	12.05	31.88	13.84	180.64**	25.57**
L1 x T6	50.50	56.00	5.50	85.00**	164.43	33.73**	14.00	17.12*	12.07	31.99	12.17	142.05	25.09**
L1 x T7	50.00	54.00	4.00	66.00	137.50	28.84	11.00	16.95	14.27**	30.38	13.00	159.20**	25.75**
L1 x T8	52.00	56.00	4.00	58.66	130.34	23.31	10.73	12.86	10.32	23.32	14.00	97.69	19.66
L2 x T1	53.00	56.00	3.00	81.75**	170.33**	32.24	11.17	15.33	10.67	37.00**	14.17	175.54**	15.33
L2 x T2	51.00	54.00	3.00	92.50**	166.17	30.34	15.67**	15.00	10.34	32.85*	14.67	139.40	15.00
L2 x T3	58.00	63.00	5.00	58.50	121.83	18.92	12.50	15.92	11.67	32.17	13.67	114.63	18.00
L2 x T4	53.00	56.00	3.00	84.00**	149.50	23.17	11.67	15.42	10.92	32.06	14.17	168.71**	23.16*
L2 x T5	56.00	59.00	3.00	63.33	122.00	25.84	19.67**	17.13*	14.20**	32.84*	14.22	142.80*	12.00
L2 x T6	54.00	58.00	4.00	85.59**	161.67	28.46	14.92	15.04	12.79	32.85*	13.00	154.80**	20.09
L2 x T7	50.00**	54.00	4.00	100.13**	198.21**	34.62**	9.84	17.97**	12.15	29.91	13.84	160.11**	20.00
L2 x T8	51.50	54.50	3.00	79.84*	144.50	36.96**	11.92	14.02	12.22	18.73	12.34	82.25	13.50
L3 x T1	54.00	57.00	3.00	82.00**	154.25	30.67	11.83	15.09	12.24	26.73	13.84	135.59	17.93
L3 x T2	50.00**	54.00	4.00	78.84*	152.67	28.17	12.67	16.67	13.50*	34.00**	14.33	135.98	19.07
L3 xT3	51.00	54.00	3.00	84.50**	173.67**	31.75	12.84	16.25	12.72	29.34	13.67	155.87**	24.17**
L3 x T4	52.00	56.00	4.00	94.00**	191.34**	27.92	11.00	14.97	11.75	30.17	14.50	180.62**	24.25**
L3 x T5	52.50	56.50	4.00	96.84**	190.67**	30.72	15.17	16.54	13.43*	31.00	14.34	167.40**	22.50
L3 x T6	54.50	58.50	4.00	86.50**	168.75*	27.59	9.34	17.84*	14.00**	23.17	14.17	125.38	18.00
L3 x T7	51.50	54.50	3.00	80.67*	169.67*	39.17	15.57*	21.00**	14.50**	32.00	14.06	148.46**	15.00
L3 x T8	50.50	54.50	4.00	105.17**	220.17**	30.00	15.83**	15.59	12.60	29.84	15.67**	173.61**	21.74
L4 x T1	46.00**	50.00**	4.00	74.17	163.17	35.92**	15.27*	15.67	11.00	29.00	14.34	135.48	17.67
L4 x T2	46.00**	50.50**	4.50	62.84	135.67	30.09	14.83	11.95	10.72	25.00	14.34	94.45	10.00
L4 xT3	51.50	56.00	4.50	63.50	130.17	24.83	10.34	10.99	9.52	29.97	14.50	122.08	10.50
L4 x T4	51.50	55.50	4.00	89.50**	167.84*	26.59	14.01	15.00	12.77	32.67*	15.33**	171.12	21.00
L4 x T5	52.00	55.00	3.00	83.67**	164.92	32.75	18.73**	13.84	10.75	27.00	13.84	116.61	19.50
L4 x T6	57.00	60.00	3.00	98.34**	193.33**	33.25*	16.17**	16.10	13.58*	33.21**	14.50	166.91**	23.93**
L4 x T7	51.50	55.50	4.00	92.34**	168.09**	30.04	14.09	17.50*	14.00**	20.50	12.19	86.01	20.06
L4 x T8	50.00**	55.00	5.00	80.00**	152.67	28.09	12.83	15.00	9.23	26.83	13.33	101.11	16.00
L5 x T1	53.00	56.00	3.00	68.67	138.33	26.83	15.00	14.75	11.00	30.84	13.84	129.79	14.67
L5 x T2	51.50	55.50	4.00	77.67	159.00	27.08	14.53	15.61	12.42	32.73*	14.67	116.64	14.67
L5 xT3	48.00**	51.00**	3.00	78.34**	143.67	33.84*	12.88	15.00	12.67	30.48	14.17	127.20	19.70
L5 x T4	50.50	56.00	5.50	72.17	136.33	30.83	16.34**	14.67	12.67	34.78**	14.17	130.74	23.50*
L5 x T5	50.00**	55.00	5.00	87.83**	153.67	28.09	12.67	12.83	11.17	32.21	13.33	104.60	14.50
L5 x T6	49.00**	53.00**	4.00	99.00**	177.67**	32.21	14.92	14.24	12.19	32.08	13.84	167.07**	26.00**

L5 x T7	52.50	56.50	4.00	78.50**	148.50	31.84	9.91	11.75	8.35	28.22	14.17	115.54	13.00
L5 x T8	54.00	57.00	3.00	77.12*	153.87	28.02	10.53	11.84	10.04	31.34	12.31	91.71	12.17
L6 x T1	53.00	56.00	3.00	85.17*	157.75	36.67**	14.00	16.67	12.25	33.00*	13.84	111.94	11.34
L6 x T2	51.00	53.00**	2.00**	81.67*	149.34	32.67	12.67	17.05*	12.64	28.03	14.67	99.91	23.50*
L6 xT3	49.50**	54.50	5.00	66.42	136.00	30.84	11.09	16.72	12.25	24.05	10.67	90.64	20.00
L6 x T4	49.00**	52.000**	3.00	61.50	105.21	26.90	13.00	15.00	11.75	33.17*	16.17**	166.82**	17.67
L6 x T5	51.00	53.00**	2.00**	67.62	102.40	21.76	13.71	16.96	10.01	31.88	12.71	109.60	14.79
L6 x T6	51.00	55.00	4.00	77.17*	135.00	27.00	13.00	16.40	11.27	27.17	14.00	135.97	21.67
L6 x T7	51.00	54.00	3.00	78.00*	156.50	39.08**	11.67	13.84	12.83	31.33	13.00	128.68	14.00
L6 x T8	51.00	56.00	5.00	72.34	128.67	31.67	13.34	16.17	11.00	31.34	13.00	94.70	16.58
L7 x T1	50.00	54.00	4.00	72.83	145.33	35.83**	13.83	16.00	10.50	38.21**	16.17**	188.88**	25.04**
L7 x T2	53.00	57.00	4.00	67.34	130.84	29.96	12.92	15.05	12.32	30.33	15.00**	149.10**	22.67*
L7 xT3	53.00	56.00	3.00	96.17**	187.33**	30.25	11.67	14.22	11.79	32.76*	15.84**	163.90**	24.73**
L7 x T4	53.00	55.50	2.50**	66.68	144.27	29.95	12.28	12.12	10.73	25.56	14.00	107.07	18.03
L7 x T5	54.00	56.00	2.00**	57.19	122.39	25.59	13.40	13.23	9.58	32.96*	13.84	124.94	15.49
L7 x T6	51.00	55.50	4.50	85.17**	158.50	32.58	15.83**	17.83*	10.34	34.87*	13.67	149.01**	20.53
L7 x T7	50.50	54.50	4.00	89.84**	178.50**	31.75	14.67	18.92**	14.10**	32.00	14.50	177.38	33.17**
L7 x T8	51.00	53.00**	2.00**	80.84**	166.00	36.17**	13.17	16.70	12.70	33.00	15.67**	117.65	20.84
L8 x T1	46.00**	51.00	5.00	93.34**	189.75**	33.25*	12.67	14.97	11.42	31.00	14.17	124.96	17.84
L8 x T2	50.50	56.00	5.50	84.48**	167.92*	34.67**	12.00	16.29	10.34	32.41	13.84	101.38	12.25
L8 xT3	50.00	54.50	4.50	84.17**	175.50**	30.42	12.67	17.75**	14.00**	38.83**	13.84	136.14	18.00
L8 x T4	50.00	54.00	4.00	70.84	138.67	30.42	11.67	15.75	12.92	34.67*	14.17	134.67	19.84
L8 x T5	51.00	55.00	4.00	74.67	147.33	30.25	15.00	16.25	11.95	31.75	14.67	118.23	15.34
L8 x T6	51.00	54.50	3.50	82.86**	180.50**	29.50	12.67	16.79	12.96	36.58**	14.00	194.02**	25.09**
L8 x T7	51.50	55.00	3.50	84.17**	149.84	30.34	12.84	15.17	12.15	30.29	14.17	120.99	15.50
L8 x T8	45.00**	50.00**	5.00	84.84**	173.00**	32.75	11.00	18.13**	13.08	38.84**	12.17	128.94	16.33
L9 x T1	50.50	53.50*	3.00	110.42**	201.00**	34.17**	14.34	17.00	13.25	29.18	14.67	90.86	12.05
L9 x T2	44.00**	49.00**	5.00	101.08**	169.50**	32.84*	13.91	15.20	12.04	27.95	16.34**	107.37	16.26
L9 xT3	48.50**	52.00**	3.50	86.84**	173.83**	27.42	13.50	17.59**	12.09	31.50	13.00	127.92	22.00
L9 x T4	48.00**	53.00**	5.00	85.84**	160.67	27.33	10.67	15.55	12.25	32.27	14.34	135.82	20.50
L9 x T5	49.00**	53.50*	4.50	90.58**	168.50*	33.00*	12.83	14.97	11.87	35.50**	16.00**	148.46**	18.83
L9 x T6	53.50	56.50	3.00	104.00**	192.34**	31.34	13.17	14.33	12.00	26.00	12.67	107.13	18.17
L9 x T7	49.50**	53.50*	4.00	100.25**	165.17	34.42**	13.00	16.78	14.10**	35.02**	14.00	170.74**	25.75**
L9 x T8	54.00	56.00	2.00**	100.93**	167.16*	35.54**	12.60	23.54**	13.17	37.78**	16.31**	174.54**	26.59**
Overall mean	51.13	54.86	3.74	68.98	158.60	30.68	13.56	15.78	12.08	31.03	14.02	134.56	19.26
CD at 5 %	0.75	0.93	0.64	7.00	8.20	2.31	1.68	1.32	1.35	1.52	0.70	7.41	3.32
CD at 1 %	1.00	1.23	0.85	9.30	10.89	3.07	2.24	1.76	1.80	2.02	0.94	9.85	4.41

Table 18. Cont.,

	SH%	SPY	100 SW	SL	SG	ST	SVI	STR	HIP	TRP	PA
L1 x T1	83.65	104.18	26.74	0.94**	0.80**	0.43**	3902.18	62.16	1.39**	0.06	5.24**
L1 x T2	83.58	122.74**	24.99	0.90**	0.70	0.37	3404.25	71.78**	0.97	0.08**	8.41
L1 xT3	80.64	95.27	24.67	0.80	0.70	0.40*	3174.87	60.82	0.83	0.06	5.16**
L1 x T4	81.13	126.42	26.03	0.77	0.67	0.30	2614.96	66.19	1.44**	0.08**	4.40**
L1 x T5	85.85	155.07**	34.01**	0.83	0.67	0.42**	4475.92*	66.62	0.95	0.07	10.27
L1 x T6	82.35	116.96	30.08**	0.95**	0.79**	0.40*	4359.95	72.59**	1.12*	0.07	7.63
L1 x T7	83.82	133.45**	33.79**	0.82	0.70	0.32	3938.72	64.54	1.32**	0.08**	5.20**
L1 x T8	79.88	78.04	23.92	0.87**	0.73**	0.32	4963.39**	69.42	0.82	0.07	9.52
L2 x T1	91.26**	160.21**	30.57**	0.90**	0.65	0.32	5097.26**	75.52**	0.85	0.09**	12.01
L2 x T2	89.24*	124.40**	25.82	0.80	0.69	0.30	4592.92**	77.17**	0.92	0.07	9.43
L2 x T3	84.30	96.63	22.00	0.70	0.69	0.32	4229.83	62.84	1.21**	0.06	6.41
L2 x T4	86.28	145.55**	32.05**	0.75	0.72**	0.32	4169.56	71.55*	1.04	0.05	7.49
L2 x T5	91.60**	130.80**	28.03**	0.77	0.63	0.32	4056.24	75.24**	0.80	0.07	9.06
L2 x T6	87.05	134.72**	31.58**	0.84	0.60	0.42**	4522.27*	56.95	1.26**	0.06	5.48**
L2 x T7	87.51	140.11**	33.87**	0.90**	0.80**	0.33	4798.69**	70.99	0.97	0.05	8.51
L2 x T8	83.59	68.75	29.78**	0.75	0.59	0.39	4128.19	70.10	1.44**	0.06	4.58**
L3 x T1	86.78	117.67	31.83**	0.80	0.63	0.34	4078.53	72.54**	1.17**	0.05	6.69**
L3 x T2	85.98	116.91	24.00	0.74	0.67	0.33	4068.25	72.04**	0.96	0.07	7.65
L3 xT3	84.50	131.70	32.87**	0.80	0.65	0.37	4366.02	75.44**	0.95	0.07	7.47
L3 x T4	86.58	156.38**	33.82**	0.84	0.64	0.50**	4163.33	77.22**	1.20**	0.07	4.91**
L3 x T5	86.57	144.91**	32.63**	0.79	0.67	0.42**	3967.48	73.35**	1.13*	0.05	7.34
L3 x T6	85.64	107.39	32.73**	0.85*	0.72**	0.39	4256.33	71.73**	1.01	0.07	6.60**
L3 x T7	89.90**	133.46**	29.66**	1.00**	0.70	0.43**	4189.33	72.72**	0.96	0.06	8.01
L3 x T8	87.48	151.88**	32.50**	0.80	0.66	0.43**	4113.13	77.74**	0.78	0.06	12.14
L4 x T1	86.96	117.81	28.34**	0.89**	0.70	0.41*	3792.52	53.41	1.36**	0.09**	6.00**
L4 x T2	89.44**	84.45	23.58	0.79	0.64	0.30	3922.85	55.61	1.27*	0.06	5.87
L4 xT3	91.39**	111.58	25.67	0.77	0.59	0.30	4226.27	65.41	1.02	0.08**	7.21
L4 x T4	87.73	150.12**	29.77**	0.90**	0.53	0.43**	3353.44	76.01**	0.77	0.07	13.02
L4 x T5	83.29	97.10	26.01	0.70	0.55	0.30	4531.17*	70.55*	1.11*	0.07	7.38
L4 x T6	85.67	142.99**	29.71**	0.80	0.73**	0.34	4564.78**	75.94**	0.87	0.06	9.38
L4 x T7	76.69	65.96	26.41	0.70	0.65	0.30	4327.62	54.84	1.12*	0.07	6.72*
L4 x T8	84.18	85.11	23.80	0.73	0.70	0.30	4219.19	61.74	1.02	0.08**	7.90
L5 x T1	88.69*	115.12	26.99	0.79	0.62	0.40*	3341.97	68.06	1.50**	0.07	5.05**
L5 x T2	87.42	101.98	21.24	0.93**	0.77**	0.30	3013.30	54.29	1.38**	0.08**	5.86**
L5 xT3	84.49	107.50	24.89	0.80	0.77**	0.37	3478.57	67.96	1.25**	0.09**	5.31**
L5 x T4	82.04	107.24	21.77	0.75	0.60	0.28	3190.31	63.38	0.96	0.05	7.46
L5 x T5	86.12	90.10	21.00	0.72	0.53	0.32	3904.18	58.23	0.94	0.06	8.55

L5 x T6	84.45	141.07**	31.80**	0.93**	0.75**	0.40*	3978.36	75.92**	0.97	0.07	6.93*
L5 x T7	88.75*	102.54	25.67	0.80	0.80**	0.40*	4597.35**	68.18	0.99	0.08**	6.69**
L5 x T8	86.69	79.54	20.60	0.70	0.50	0.30	3024.69	61.69	0.97	0.08**	8.92
L6 x T1	89.84**	100.61	22.00	0.82	0.82**	0.40*	3477.75	61.48	0.87	0.08**	10.87
L6 x T2	76.53	76.40	18.61	0.83	0.73**	0.32	3374.32	65.06	0.93	0.07	7.42
L6 xT3	78.36	70.64	27.58	0.80	0.72**	0.34	4449.56*	61.72	1.02	0.08**	7.15
L6 x T4	89.41**	149.15**	27.83*	0.87**	0.64	0.42**	4994.11**	76.94**	0.85	0.07	8.40
L6 x T5	86.49	94.81	23.42	0.60	0.50	0.42**	4241.18	67.50	0.78	0.08**	9.75
L6 x T6	84.06	114.30	30.05**	0.80	0.67	0.33	3237.09	72.03	0.81	0.07	8.77
L6 x T7	89.13*	114.68	28.18*	0.83	0.67	0.37	4840.41**	65.46	1.33**	0.07	6.09**
L6 x T8	82.50	78.13	19.21	0.72	0.70	0.32	5372.40**	53.94	1.00	0.07	7.63
L7 x T1	86.75	163.84**	26.53	0.70	0.65	0.40*	5172.44**	61.57	1.03	0.07	7.25
L7 x T2	84.80	126.44**	27.80*	0.70	0.62	0.43**	5493.58**	74.58**	1.22*	0.08**	5.56
L7 xT3	84.91	139.17**	26.83	0.87	0.69	0.30	5000.06**	77.52**	1.10	0.04	7.24
L7 x T4	83.15	89.04	24.89	0.80	0.40	0.30	3123.28	70.59	0.77	0.08**	9.92
L7 x T5	87.60	109.45	24.00	0.72	0.45	0.30	5369.93**	73.07	0.66	0.07	11.23
L7 x T6	86.22	128.48**	26.98	0.79	0.57	0.30	4935.49**	70.86*	1.35**	0.05	6.81*
L7 x T7	81.32	144.22**	31.09**	0.80	0.85**	0.40*	4713.58**	73.18**	0.74	0.04	12.08
L7 x T8	82.27	96.82	18.73	0.79	0.63	0.30	4023.35	70.32	1.02	0.06	7.44
L8 x T1	85.72	107.12	24.39	0.84	0.72**	0.30	2775.08	70.17	0.95	0.08**	8.81
L8 x T2	87.92	89.13	19.89	0.80	0.75**	0.40*	3833.98	61.93	1.00	0.08**	7.66
L8 xT3	86.78	118.14	22.00	0.75	0.59	0.30	5830.37**	73.55**	0.94	0.06	8.89
L8 x T4	85.34	114.84	23.39	0.80	0.80**	0.37	3942.05	73.92**	0.83	0.05	9.63
L8 x T5	87.03	102.89	22.11	0.74	0.67	0.39	4674.43**	62.85	0.97	0.06	7.97
L8 x T6	87.08	168.94**	32.99*	0.95**	0.80**	0.42**	5218.45**	76.84**	0.81	0.06	10.95
L8 x T7	87.19	105.49	24.59	0.79	0.60	0.30	4568.56**	63.04	1.00	0.05	7.20
L8 x T8	87.33	112.60	23.84	0.80	0.67	0.30	5036.24**	71.39**	1.13*	0.04	9.07
L9 x T1	86.75	78.82	18.45	0.82	0.67	0.33	3789.92	52.94	1.01	0.07	6.94*
L9 x T2	84.86	91.11	19.97	0.67	0.50	0.30	3633.46	70.99	1.42**	0.04	5.62
L9 xT3	82.78	105.92	25.88	0.85*	0.70	0.37	5122.18**	67.49	0.98	0.07	7.60
L9 x T4	84.92	115.32	24.97	0.85*	0.72**	0.48**	4222.06	72.47**	1.07	0.06	7.79
L9 x T5	87.32	129.63**	22.85	0.80	0.52	0.32	4846.86**	69.52	0.95	0.07	8.75
L9 x T6	83.05	88.96	27.01	0.84	0.72**	0.37	3689.33	57.26	0.99	0.07	7.23
L9 x T7	84.96	144.99**	29.57**	0.80	0.65	0.37	4657.49**	77.44**	0.96	0.08**	8.37
L9 x T8	84.75	147.96**	24.00	0.60	0.50	0.30	3558.89	65.75	0.67	0.04	11.27
Overall mean	**85.50**	**115.28**	**26.45**	**0.80**	**0.66**	**0.35**	**4198.83**	**68.08**	**1.03**	**0.07**	**7.87**
CD at 5 %	2.83	6.68	1.21	0.05	0.05	0.05	250.53	2.47	0.08	0.01	0.88
CD at 1 %	3.76	8.88	1.61	0.06	0.06	0.07	332.77	3.29	0.11	0.01	1.18

Table 19. *sca* of the 72 hybrids in Environment -2

Code No	50 DT	50 DS	ASI	CPH	PH	TL	TBR	CL	CG	NKr/R	NR/C	CW	SHW
L1 x T1	0.98 **	1.35 **	0.37	0.24	-3.65	-2.33 **	-0.28	0.06	0.81	-0.03	-1.04 **	-14.99 **	-1.03
L1 x T2	1.03 **	0.07	-0.97 **	8.09 **	9.93 **	0.83	1.15 *	1.47 **	1.22 *	4.41 **	0.12	21.37 **	2.12
L1 xT3	0.53 *	-1.26 **	-1.80 **	13.86 **	10.84 **	1.72	10.80 **	-0.33	-0.86	-2.27 **	0.78 **	-14.61 **	-1.61
L1 x T4	-0.08	-0.04	0.03	3.55	16.44 **	3.87 **	1.95 **	1.66 **	1.66 **	0.83	-0.21	-11.58 **	0.51
L1 x T5	-0.13	0.29	0.42	15.68 **	21.65 **	5.66 **	-0.26	0.79	-0.36	0.57	0.38	41.58 **	3.46 **
L1 x T6	-2.08 **	-0.60	1.48 **	-2.31	-3.84	3.02 **	-3.24 **	0.34	-1.03 *	1.70 **	-0.76 **	-11.35 **	-1.47
L1 x T7	-1.08 **	-0.88 *	0.20	-17.56 **	-24.08 **	-4.59 **	-4.97 **	-0.29	0.58	1.00	-0.03	14.16 **	1.01
L1 x T8	0.81 **	1.07 **	0.26	-21.55 **	-27.29 **	-8.16 **	-5.17 **	-3.71 **	-2.02 **	-6.21 **	0.76 **	-24.58 **	-2.99 *
L2 x T1	-0.02	-0.34	-0.32	0.02	10.38 **	1.19	-2.45 **	-0.38	-0.85	5.19 **	0.26	32.51 **	0.55
L2 x T2	-0.97 **	-1.62 **	-0.65 **	12.53 **	15.63 **	1.36	1.73 **	-0.55	-1.48 **	1.94 **	0.27	10.43 **	-0.38
L2 x T3	4.53 **	6.05 **	1.51 **	-18.69 **	-30.19 **	-7.97 **	-1.18 *	0.39	-0.19	1.37 *	0.25	-21.61 **	0.13
L2 x T4	-0.08	-0.73 *	-0.65 **	7.33 **	2.80	-3.35 **	-1.30 **	0.38	-1.14 *	0.09	-0.07	12.44 **	3.67 **
L2 x T5	1.87 **	1.60 **	-0.26	-14.50 **	-22.61 **	-1.51	4.40 **	1.79 **	2.75 **	0.93	0.40	0.25	-3.48 **
L2 x T6	-0.58 *	-0.28	0.29	-2.11	-4.24	-0.30	1.42 **	-1.09 *	0.65	1.95 **	-0.29	-2.09	0.15
L2 x T7	-3.08 **	-2.56 **	0.51 *	16.18 **	38.99 **	3.14 **	-2.39 **	1.37 **	-0.57	-0.07	0.44	11.58 **	1.88
L2 x T8	-1.69 **	-2.12 **	-0.43	-0.76	-10.76 **	7.44 **	-0.23	-1.91 **	0.84	-11.40 **	-1.26 **	-43.52 **	-2.52
L3 x T1	2.29 **	1.85 **	-0.44	-7.59 **	-29.07 **	-2.31 **	-1.41 **	-1.64 **	-0.51	-3.57 **	-0.63 *	-18.02 **	-0.05
L3 x T2	-0.65 *	-0.43	0.22	-8.99 **	-21.25 **	-2.73 **	-0.89	0.10	0.46	4.61 **	-0.63 *	-3.58	0.50
L3 xT3	-1.15 **	-1.76 **	-0.61 *	-0.55	-1.73	2.93 **	-0.46	-0.29	-0.36	0.06	-0.32	9.04 **	3.10 *
L3 x T4	0.24	0.46	0.22	9.48 **	21.27 **	-0.53	-1.58 **	-1.08 *	-1.53 **	-0.28	-0.30	13.77 **	1.56
L3 x T5	-0.32	0.29	0.61 *	11.14 **	22.69 **	1.44	0.30	0.19	0.75	0.61	-0.05	14.27 **	3.82 **
L3 x T6	1.24 **	1.40 **	0.17	-9.05 **	-20.53 **	-3.11 **	-3.77 **	0.69	0.64	-6.21 **	0.31	-42.09 **	-5.13 **
L3 x T7	-0.26	-0.88 *	-0.61 *	-11.14 **	-12.92 **	5.76 **	3.74 **	3.39 **	0.55	3.54 **	0.11	-10.66 **	-6.31 **
L3 x T8	-1.38 **	-0.93 **	0.44	16.71 **	41.54 **	-1.45	4.07 **	-1.36 **	-0.00	1.23 *	1.50 **	37.27 **	2.51
L4 x T1	-4.40 **	-4.22 **	0.18	-7.40 **	-1.98	3.50 **	0.53	1.18 *	-0.10	0.22	0.15	10.51 **	2.69 *
L4 x T2	-3.34 **	-2.99 **	0.35	-16.97 **	-20.08 **	-0.26	-0.22	-2.38 **	-0.68	-2.88 **	-0.35	-16.46 **	-5.57 **
L4 xT3	0.66 *	1.17 **	0.51 *	-13.53 **	-27.06 **	-3.44 **	-4.46 **	-3.32 **	-1.92 **	2.20 **	0.79 **	3.90	-7.56 **
L4 x T4	1.05 **	0.90 **	-0.15	13.00 **	15.93 **	-1.30	-0.07	1.19 *	1.14 *	3.73 **	0.81 **	32.91 **	1.32
L4 x T5	0.49	-0.27	-0.76 **	6.00 *	15.10 **	4.02 **	2.35 **	-0.28	-0.28	-1.88 **	-0.27	-7.88 **	3.82 **
L4 x T6	5.05 **	3.84 **	-1.21 **	10.80 **	22.22 **	3.11 **	1.56 **	1.19 *	1.86 **	5.34 **	0.92 **	28.08 **	3.80 **
L4 x T7	1.05 **	1.06 **	0.01	8.55 **	3.67	-2.81 **	0.75	2.13 **	1.70 **	-6.45 **	-1.49 **	-44.46 **	1.74
L4 x T8	-0.56 *	0.51	1.07 **	-0.44	-7.79 **	-2.81 **	-0.44	0.30	-1.73 **	-0.27	-0.56 *	-6.59 *	-0.22
L5 x T1	2.23 **	1.47 **	-0.76 **	-12.27 **	-18.72 **	-5.24 **	1.45 **	0.93	0.04	-1.51 **	-0.12	6.13 *	-0.26
L5 x T2	1.78 **	1.69 **	-0.09	-1.51	11.35 **	-2.92 **	0.66	1.94 **	1.16 *	1.29 *	0.22	7.04 *	-0.85
L5 xT3	-3.22 **	-4.14 **	-0.92 **	1.94	-5.46	5.92 **	-0.73	1.36 **	1.37 **	-0.85	0.69 *	10.32 **	1.69
L5 x T4	-0.33	1.08 **	1.41 **	-3.71	-7.47 *	3.29 **	3.44 **	1.53 **	1.17 *	2.28 **	-0.12	-6.16 *	3.87 **
L5 x T5	-1.88 **	-0.58	1.30 **	10.79 **	11.95 **	-0.29	-2.52 **	-0.61	0.27	-0.23	-0.54 *	-18.58 **	-1.12
L5 x T6	-3.33 **	-3.47 **	-0.15	12.10 **	14.66 **	2.42 **	1.50 **	-0.00	0.60	0.65	0.49	29.55 **	5.93 **

L5 x T7	1.67 **	1.75 **	0.08	-4.65	-7.82 **	-0.66	-2.24 **	-2.95 **	-3.82 **	-2.29 **	0.72 **	-13.62 **	-5.26 **
L5 x T8	3.06 **	2.19 **	-0.87 **	-2.68	1.51	-2.53 **	-1.55 **	-2.20 **	-0.79	0.67	-1.34 **	-14.69 **	-4.00 **
L6 x T1	2.48 **	2.28 **	-0.19	10.41 **	18.22 **	3.61 **	0.99 *	0.58	0.85	2.24 **	0.19	-6.09 *	-3.76 **
L6 x T2	1.53 **	0.01	-1.53 **	8.67 **	19.21 **	1.69	-0.66	1.12 *	0.94	-1.82 **	0.52	-4.06	7.82 **
L6 xT3	-1.47 **	0.17	1.64 **	-3.81	4.40	1.94 *	-1.99 **	0.81	0.51	-5.70 **	-2.50 **	-20.60 **	1.82
L6 x T4	-1.58 **	-2.10 **	-0.53 *	-8.20 **	-21.07 **	-1.62	0.64	-0.41	-0.19	2.26 **	2.18 **	35.55 **	-2.13
L6 x T5	-0.63 *	-1.77 **	-1.14 **	-3.24	-21.79 **	-7.59 **	-0.94	1.25 *	-1.32 *	1.02	-0.86 **	-7.95 **	-1.00
L6 x T6	-1.08 **	-0.66	0.42	-3.56	-10.49 **	-3.77 **	0.12	-0.10	-0.75	-2.67 **	0.96 **	4.08	1.43
L6 x T7	0.42	0.06	-0.36	1.02	17.70 **	5.60 **	0.05	-3.13 **	0.22	2.41 **	-0.14	5.15	-4.43 **
L6 x T8	0.31	2.01 **	1.69 **	-1.29	-6.17 *	0.14	1.79 **	-0.13	-0.26	2.26 **	-0.35	-6.06 *	0.24
L7 x T1	-1.65 **	-0.72 *	0.93 **	-5.20 *	-14.49 **	2.09 *	0.15	0.51	-0.66	4.99 **	1.19 **	40.89 **	4.83 **
L7 x T2	2.41 **	3.01 **	0.60 *	-8.93 **	-19.58 **	-1.70	-1.07 *	-0.28	0.86	-1.99 **	-0.48	15.17 **	1.87
L7 xT3	0.91 **	0.67 *	-0.24	22.67 **	35.44 **	0.67	-2.07 **	-1.09 *	0.29	0.55	1.34 **	22.69 **	1.43
L7 x T4	1.30 **	0.40	-0.90 **	-6.29 *	-2.30	0.74	-0.75	-2.70 **	-0.96	-7.82 **	-1.31 **	-54.16 **	-6.89 **
L7 x T5	1.24 **	0.23	-1.01 **	-16.94 **	-22.09 **	-4.45 **	-1.91 **	-1.89 **	-1.51 **	-0.36	-1.06 **	-22.57 **	-5.42 **
L7 x T6	-2.20 **	-1.16 **	1.04 **	1.17	-7.27 *	1.13	2.29 **	1.92 **	-1.44 **	2.56 **	-0.70 *	-12.84 **	-4.83 **
L7 x T7	-1.20 **	-0.44	0.76 **	9.59 **	19.42 **	-2.42 **	2.39 **	2.54 **	1.74 **	0.61	0.03	23.89 **	9.62 **
L7 x T8	-0.81 **	-1.99 **	-1.18 **	3.94	10.87 **	3.95 **	0.96 *	0.99	1.68 **	1.46 *	0.99 **	-13.07 **	-0.62
L8 x T1	-3.08 **	-2.28 **	0.81 **	9.89 **	18.76 **	-0.43	-0.11	-1.40 **	-0.58	-4.05 **	0.15	-8.21 **	2.66 *
L8 x T2	2.47 **	3.44 **	0.97 **	2.79	6.34 *	3.06 **	-1.08 *	0.08	-1.96 **	-1.75 **	-0.68 *	-17.72 **	-3.51 **
L8 xT3	0.47	0.61	0.14	5.26 *	12.44 **	0.89	-0.16	1.56 **	1.66 **	4.79 **	0.30	9.76 **	-0.25
L8 x T4	0.86 **	0.33	-0.53 *	-7.54 **	-19.07 **	1.27	-0.45	0.06	0.38	-0.54	-0.19	-11.74 **	-0.04
L8 x T5	0.81 **	0.67 *	-0.14	-4.88	-8.31 **	0.27	0.59	0.26	0.01	-3.41 **	0.73 **	-14.46 **	-0.54
L8 x T6	0.36	-0.72 *	-1.08 **	-6.55 *	3.56	-1.89 *	0.03	-0.00	0.34	2.44 **	0.59 *	47.00 **	4.77 **
L8 x T7	2.36 **	1.50 **	-0.86 **	-1.50	-20.42 **	-3.77 **	1.47 **	-2.09 **	-1.06 *	-2.94 **	0.66 *	-17.67 **	-3.01 *
L8 x T8	-4.25 **	-3.56 **	0.69 **	2.53	6.70 *	0.60	-0.30	1.54 **	1.22 *	5.46 **	-1.55 **	13.04 **	-0.08
L9 x T1	1.17 **	0.60	-0.57 *	11.90 **	20.56 **	-0.07	1.13 *	0.15	1.00	-3.48 **	-0.14	-42.74 **	-5.62 **
L9 x T2	-4.28 **	-3.18 **	1.10 **	4.32	-1.54	0.67	0.39	-1.50 **	-0.51	-3.81 **	1.03 **	-12.17 **	-2.00
L9 xT3	-1.28 **	-1.51 **	-0.24	-7.15 **	1.31	-2.66 **	0.24	0.91	-0.50	-0.15	-1.33 **	1.10	1.25
L9 x T4	-1.39 **	-0.29	1.10 **	-7.62 **	-6.52 *	-2.37 **	-1.88 **	-0.63	-0.53	-0.55	-0.81 **	-11.02 **	-1.87
L9 x T5	-1.44 **	-0.46	0.99 **	-4.04	3.40	2.46 **	-2.02 **	-1.51 **	-0.31	2.74 **	1.27 **	15.34 **	0.46
L9 x T6	2.61 **	1.65 **	-0.96 **	-0.48	5.94 *	-0.61	0.09	-2.94 **	-0.87	-5.75 **	-1.53 **	-40.34 **	-4.65 **
L9 x T7	0.11	0.38	0.26	-0.48	-14.54 **	-0.25	1.19 *	-0.96	0.65	4.19 **	-0.30	31.64 **	4.75 **
L9 x T8	4.50 **	2.82 **	-1.68 **	3.55	-8.60 **	2.83 **	0.86	6.47 **	1.06 *	6.80 **	1.80 **	58.20 **	7.68 **

Table 19. Cont.,

	SH%	SPY	100 SW	SL	SG	ST	SVI	STR	HIP	TRP	PA
L1 x T1	-0.83	-13.95 **	-1.04 *	0.05 *	0.05 *	0.05 *	310.33 **	-0.73	0.19 **	-0.02 **	-1.70 **
L1 x T2	0.94	19.25 **	0.54	0.05 *	-0.03	0.02	-177.52	6.04 **	-0.23 **	0.00	2.07 **
L1 xT3	-0.71	-13.00 **	-2.72 **	-0.05 **	-0.03	0.05 *	-911.44 **	-5.95 **	-0.27 **	-0.01 **	-1.07 **
L1 x T4	-1.15	-12.09 **	-2.71 **	-0.10 **	-0.02	-0.09 **	-793.05 **	-4.53 **	0.37 **	0.01 **	-1.72 **
L1 x T5	1.87	38.12 **	6.43 **	0.03	0.04	0.05 *	368.54 **	-0.61	-0.04	0.00	2.06 **
L1 x T6	0.18	-9.89 **	-1.82 **	0.03	0.02	0.01	397.61 **	3.89 **	0.02	-0.00	0.59 *
L1 x T7	1.24	13.14 **	3.02 **	-0.06 **	-0.07 **	-0.05 *	-231.37 *	-1.97 *	0.20 **	0.01 *	-1.74 **
L1 x T8	-1.52	-21.59 **	-1.70 **	0.06 **	0.04 *	-0.02	1036.89 **	3.84 **	-0.24 **	0.01	1.52 **
L2 x T1	1.78	31.97 **	1.61 **	0.07 **	-0.05 *	-0.04	910.32 **	9.35 **	-0.31 **	0.02 **	4.36 **
L2 x T2	1.61	10.80 **	0.19	0.01	0.01	-0.02	416.06 **	8.16 **	-0.23 **	0.00	2.38 **
L2 x T3	-2.04	-21.74 **	-6.58 **	-0.09 **	0.00	-0.01	-451.57 **	-7.21 **	0.14 **	-0.01 *	-0.53 *
L2 x T4	-1.00	8.77 **	2.12 **	-0.06 **	0.07 **	-0.04 *	166.46	-2.44 *	0.02	-0.01 **	-0.79 **
L2 x T5	2.63 *	3.74	-0.74	0.03	0.05 *	-0.02	-646.23 **	4.73 **	-0.15 **	0.01 *	0.14
L2 x T6	-0.12	-2.24	-1.50 **	-0.02	-0.11 **	0.06 **	-35.16	-15.03 **	0.21 **	-0.00	-2.27 **
L2 x T7	-0.07	9.70 **	1.91 **	0.07 **	0.08 **	-0.01	33.51	1.20	-0.10 **	-0.01 **	0.86 **
L2 x T8	-2.81 *	-40.99 **	2.98 **	0.00	-0.05 **	0.08 **	-393.40 **	1.23	0.42 **	0.00	-4.14 **
L3 x T1	-1.77	-17.97 **	0.83	-0.06 **	-0.07 **	-0.08 **	190.66 *	2.31 *	0.06	-0.02 **	-0.69 **
L3 x T2	-0.72	-4.08	-3.67 **	-0.08 **	-0.01	-0.06 *	190.46 *	-1.02	-0.15 **	0.00	0.86 **
L3 xT3	-0.92	5.94 *	2.25 **	-0.02	-0.03	-0.02	-16.31	1.34	-0.08 *	0.01 **	0.80 **
L3 x T4	0.23	12.21 **	1.85 **	-0.00	-0.00	0.08 **	459.30 **	-0.83	0.22 **	0.01	-3.09 **
L3 x T5	-1.48	10.45 **	1.82 **	0.02	0.09 **	0.02	-435.92 **	-1.21	0.22 **	-0.01 **	-1.31 **
L3 x T6	-0.59	-36.96 **	-2.40 **	-0.03	0.01	-0.03	-2.03	-4.30 **	0.00	0.01 **	-0.88 **
L3 x T7	3.25 **	-4.34	-4.34 **	0.15 **	-0.02	0.03	-276.77 **	-1.11	-0.07 *	0.00	0.62 *
L3 x T8	2.01	34.75 **	3.66 **	0.03	0.03	0.05 *	-109.39	4.83 **	-0.20 **	-0.00	3.69 **
L4 x T1	-0.58	7.82 **	1.93 **	0.07 **	0.03	0.06 *	-62.28	-6.90 **	0.19 **	0.01 *	-1.72 **
L4 x T2	3.75 **	-10.89 **	0.50	0.01	-0.01	-0.02	78.13	-7.55 **	0.11 **	-0.01 **	-1.25 **
L4 xT3	6.99 **	11.46 **	-0.36	-0.01	-0.06 **	-0.02	-122.98	1.22	-0.05	0.00	0.21
L4 x T4	2.39 *	31.59 **	2.40 **	0.10 **	-0.08 **	0.07 **	-317.52 **	7.87 **	-0.26 **	0.00	4.69 **
L4 x T5	-3.75 **	-11.71 **	-0.20	-0.02	0.00	-0.03	160.84	5.90 **	0.15 **	-0.00	-1.61 **
L4 x T6	0.45	24.29 **	-0.82	-0.04 *	0.05 *	-0.01	339.49 **	9.82 **	-0.19 **	-0.01 **	1.56 **
L4 x T7	-8.95 **	-46.20 **	-3.00 **	-0.11 **	-0.04	-0.04	-105.42	-9.09 **	0.04	0.00	-1.00 **
L4 x T8	-0.28	-6.37 *	-0.45	-0.00	0.10 **	-0.01	29.74	-1.27	0.00	0.01 **	-0.88 **
L5 x T1	0.74	6.39 *	2.99 **	-0.05 *	-0.08 **	0.04	38.30	7.22 **	0.28 **	-0.01 *	-1.57 **
L5 x T2	1.31	7.89 **	0.58	0.13 **	0.09 **	-0.03	-280.28 **	-9.39 **	0.17 **	0.01	-0.17
L5 xT3	-0.32	8.64 **	1.28 **	0.01	0.09 **	0.04	-319.55 **	3.25 **	0.13 **	0.01 **	-0.61 *
L5 x T4	-3.71 **	-10.03 **	-3.18 **	-0.06 **	-0.04	-0.09 **	70.49	-5.28 **	-0.12 **	-0.02 **	0.21

L5 x T5	-1.33	-17.46 **	-2.79 **	-0.02	-0.05 *	-0.03	84.99	-6.95 **	-0.07 *	-0.02 **	0.65 *
L5 x T6	-1.19	23.62 **	3.68 **	0.07 **	0.04 *	0.04	304.21 **	9.27 **	-0.14 **	0.00	0.20
L5 x T7	2.70 *	-8.36 **	-1.33 **	-0.03	0.08 **	0.05 *	715.45 **	3.73 **	-0.15 **	0.01 **	0.06
L5 x T8	1.81	-10.69 **	-1.23 **	-0.05 *	-0.13 **	-0.02	-613.62 **	-1.84	-0.10 **	0.01 **	1.22 **
L6 x T1	3.43 **	-2.33	-2.36 **	0.00	0.10 **	0.02	-508.17 **	-0.16	-0.17 **	-0.00	2.83 **
L6 x T2	-8.04 **	-11.89 **	-2.42 **	0.05 **	0.04 *	-0.03	-601.52 **	0.58	-0.11 **	-0.00	-0.03
L6 xT3	-4.92 **	-22.42 **	3.60 **	0.03	0.02	-0.01	-30.82	-3.80 **	0.07 *	0.00	-0.18
L6 x T4	5.20 **	37.68 **	2.51 **	0.07 **	-0.02	0.03	1192.03 **	7.48 **	-0.06	0.00	-0.26
L6 x T5	0.58	-6.95 **	-0.74	-0.12 **	-0.09 **	0.05 *	-260.27 **	1.51	-0.06	0.01 **	0.44
L6 x T6	-0.04	2.65	1.57 **	-0.04 *	-0.05 *	-0.05 *	1119.32 **	4.58 **	-0.13 **	-0.00	0.62 *
L6 x T7	4.62 **	9.58 **	0.82	0.02	-0.06 **	0.00	276.25 **	0.20	0.37 **	-0.00	-1.95 **
L6 x T8	-0.83	-6.30 *	-2.99 **	-0.02	0.05 **	-0.02	1051.83 **	-10.40 **	0.10 **	-0.00	-1.48 **
L7 x T1	0.25	36.06 **	0.93 *	-0.10 **	0.01	0.04 *	705.91 **	-6.01 **	-0.05	0.00	-0.97 **
L7 x T2	0.15	13.30 **	5.52 **	-0.06 **	-0.00	0.10 **	1037.13 **	4.15 **	0.15 **	0.02 **	-2.06 **
L7 xT3	1.55	21.26 **	1.61 **	0.11 **	0.07 **	-0.03	39.07	6.05 **	0.11 **	-0.02 **	-0.27
L7 x T4	-1.15	-47.28 **	-1.68 **	0.02	-0.18 **	-0.06 **	1159.41 **	-4.82 **	-0.18 **	0.02 **	1.07 **
L7 x T5	1.60	-17.15 **	-1.40 **	0.01	-0.07 **	-0.04	387.87 **	1.14	-0.22 **	0.01 **	1.73 **
L7 x T6	2.03	-8.02 **	-2.75 **	-0.04 *	-0.08 **	-0.06 **	98.46	-2.53 **	0.37 **	-0.01 *	-1.51 **
L7 x T7	-3.28 **	14.27 **	2.49 **	0.01	0.19 **	0.06 *	-331.19 **	1.98 *	-0.26 **	-0.02 **	3.86 **
L7 x T8	-1.15	-12.46 **	-4.71 **	0.07 **	0.06 **	-0.01	-777.83 **	0.04	0.08 *	0.00	-1.85 **
L8 x T1	-2.95 **	-10.87 **	0.49	-0.00	-0.01	-0.06 **	1447.39 **	4.83 **	-0.10 **	0.01 **	0.26
L8 x T2	1.09	-14.21 **	-0.68	-0.00	0.04 *	0.07 **	-378.40 **	-6.25 **	-0.04	0.01 **	-0.29
L8 xT3	1.25	10.02 **	-1.52 **	-0.05 *	-0.13 **	-0.03	1113.45 **	4.34 **	-0.02	0.00	1.05 **
L8 x T4	-1.13	-11.69 **	-1.48 **	-0.02	0.13 **	0.00	-96.58	0.76	-0.09 **	-0.01 *	0.45
L8 x T5	-1.14	-13.92 **	-1.59 **	-0.01	0.05 **	0.04	-63.57	-6.83 **	0.12 **	0.00	-1.85 **
L8 x T6	0.72	42.23 **	4.97 **	0.08 **	0.06 **	0.05 *	625.50 **	5.70 **	-0.14 **	0.00	2.30 **
L8 x T7	0.42	-14.67 **	-2.31 **	-0.05 *	-0.15 **	-0.05 *	-232.14 *	-5.91 **	0.03	-0.01 *	-1.36 **
L8 x T8	1.74	13.12 **	2.11 **	0.04 *	-0.00	-0.02	479.13 **	3.36 **	0.22 **	-0.02 **	-0.55 *
L9 x T1	-0.05	-37.12 **	-5.39 **	0.01	0.01	-0.04	-137.67	-9.92 **	-0.09 **	0.00	-0.79 **
L9 x T2	-0.09	-10.18 **	-0.54	-0.10 **	-0.13 **	-0.04	-284.05 **	5.29 **	0.33 **	-0.03 **	-1.51 **
L9 xT3	-0.88	-0.15	2.42 **	0.08 **	0.07 **	0.03	700.13 **	0.76	-0.03	0.01 *	0.59 *
L9 x T4	0.33	-9.15 **	0.17	0.06 **	0.12 **	0.10 **	478.30 **	1.79	0.10 **	-0.00	-0.56 *
L9 x T5	1.02	14.87 **	-0.79	0.08 **	-0.02	-0.04	403.74 **	2.32 *	0.05	0.00	-0.25
L9 x T6	-1.43	-35.69 **	-0.94 *	-0.00	0.05 **	-0.01	-608.76 **	-11.41 **	-0.01	0.01 **	-0.60 *
L9 x T7	0.07	26.89 **	2.74 **	-0.00	-0.02	0.01	151.66	10.97 **	-0.06	0.02 **	0.64 *
L9 x T8	1.03	50.53 **	2.33 **	-0.13 **	-0.09 **	-0.03	-703.35 **	0.20	-0.29 **	-0.02 **	2.47 **

Table 20. Mean performance of the 72 hybrids in Environment 3

Code No	50 DT	50 DS	ASI	CPH	PH	TL	TBR	CL	CG	NKr/R	NR/C	CW	SHW
L1 x T1	52.00	56.00	4.00	114.00**	199.17**	32.08	16.33**	19.90**	13.37	29.33	13.33	137.10	32.67**
L1 x T2	46.50**	50.50	4.00	78.33	161.92	33.92	13.50	15.78	13.05	24.67	14.00	110.18	15.17
L1 xT3	52.50	56.00	3.50	80.01	140.21	34.55	13.07	15.07	14.61*	24.79	12.00	83.22	13.83
L1 x T4	52.00	55.00	3.00	99.08**	208.43**	31.00	11.67	18.00**	16.50**	34.33*	15.00	170.45**	28.17**
L1 x T5	54.00	58.00	4.00	88.13	167.13	26.38	11.75	19.50**	13.63	34.83*	13.67	162.79**	24.50**
L1 x T6	52.00	55.00	3.00	91.00	185.50**	42.56**	11.50	16.08	14.68*	26.21	13.67	141.32	26.42**
L1 x T7	52.00	54.00	2.00**	90.83	183.92**	40.35**	13.17	19.17**	13.75	35.33**	13.67	199.67**	33.75**
L1 x T8	54.00	57.00	3.00	83.30	168.52	40.21**	14.43	14.05	15.00**	35.55**	15.10	174.52**	32.00**
L2 x T1	54.00	59.00	5.00	92.33	181.00**	34.42	13.67	16.23	12.55	34.50*	15.00	161.29**	17.77
L2 x T2	52.00	55.50	3.50	104.75**	201.33**	36.38*	14.83	17.80*	12.95	26.67	14.50	108.83	14.08
L2 x T3	57.50	61.00	3.50	57.33	115.67	12.00	3.00	14.33	11.00	27.83	13.33	115.86	16.83
L2 x T4	53.50	56.50	3.00	80.42	158.83	32.07	10.83	17.83*	12.92	35.76**	14.00	151.95**	17.42
L2 x T5	55.50	58.50	3.00	104.83**	191.62**	31.42	13.83	17.00	12.40	28.43	14.67	139.72	24.00**
L2 x T6	53.50	57.00	3.50	88.58	188.03**	38.40**	10.17	18.00**	14.33*	33.20	13.84	189.07**	24.00**
L2 x T7	50.00**	53.50*	3.50	104.42**	224.17**	31.70	13.00	16.63	13.50	33.92*	15.00	158.28**	24.67**
L2 x T8	51.50	54.50	3.00	75.79	166.13	36.46*	12.25	17.75*	13.00	28.71	15.00	122.24	15.75
L3 x T1	52.50	55.00	2.50*	109.00**	201.58**	25.53	12.33	19.33**	12.00	36.50**	14.83	169.42**	17.94
L3 x T2	50.00**	53.00**	3.00	82.33	161.92	28.62	17.00**	15.00	9.63	30.50	13.83	133.64	20.83
L3 xT3	51.50	54.50	3.00	113.00**	214.00**	36.52*	16.17**	14.85	12.12	26.08	15.04	126.98	21.17
L3 x T4	52.50	55.50	3.00	70.00	144.00	34.15	11.83	18.00**	17.67**	38.20**	15.67*	193.32**	27.08**
L3 x T5	53.50	56.50	3.00	89.33	184.97**	29.67	16.00*	13.43	11.90	24.56	14.50	102.36	16.67
L3 x T6	55.50	59.00	3.50	104.17**	195.25**	30.48	10.67	17.00	13.50	37.00**	14.50	185.94**	31.44**
L3 x T7	50.00**	54.00	4.00	82.83	170.83	39.58**	16.17**	16.55	12.93	29.50	13.00	150.25**	19.25
L3 x T8	51.50	56.00	4.50	81.75	176.75*	39.25**	12.17	17.00	13.33	33.25	14.48	152.45**	21.83
L4 x T1	46.00**	52.00**	6.00	87.50	182.92**	39.25**	14.50	18.23**	12.50	22.67	13.34	98.41	18.83
L4 x T2	46.50**	50.50**	4.00	74.17	154.92	33.85	10.83	16.17	11.67	32.00	15.33	129.93	20.44
L4 xT3	52.50	56.50	4.00	61.83	141.60	32.98	11.00	16.92	12.03	36.65**	15.00	127.59	16.33
L4 x T4	52.00	55.50	3.50	101.23**	180.15**	35.85	12.83	18.30**	14.47*	36.00**	17.67**	176.57**	25.00**
L4 x T5	55.00	57.50	2.50*	69.60	151.08	36.02	16.67**	13.53	12.83	26.17	13.67	97.10	16.69
L4 x T6	54.50	58.50	4.00	109.07**	198.13**	34.88	14.33	16.42	13.67	34.46*	14.34	145.77*	18.30
L4 x T7	51.50	54.50	3.00	94.08	193.62**	40.25**	14.67	17.00	12.67	32.83	15.33	120.57	20.33
L4 x T8	50.00**	54.50	4.50	91.50	181.83**	39.33**	12.67	15.73	12.73	30.25	15.00	135.02	20.00
L5 x T1	53.50	56.50	3.00	59.50	142.00	29.83	16.33**	15.10	12.57	27.67	15.67*	111.45	17.00
L5 x T2	50.00**	54.00	4.00	86.17	177.17*	35.02	10.33	13.00	12.17	24.67	13.00	110.12	20.50
L5 xT3	46.00**	50.00**	4.00	63.83	136.75	28.67	14.83	13.50	11.92	31.50	14.67	127.37	17.58
L5 x T4	48.50**	53.00**	4.50	70.42	141.50	31.83	15.67	15.00	12.00	33.50	14.33	132.58	14.67
L5 x T5	50.00*	55.50*	5.50	74.08	159.75	32.17	18.08**	14.43	13.37	29.04	16.33**	129.26	14.34
L5 x T6	50.50	53.50*	3.00	97.75**	176.25*	34.83	14.75	16.66	13.21	29.58	14.33	157.40**	18.75

L5 x T7	52.50	56.50	4.00	76.33	166.27	34.82	11.17	16.37	14.33*	28.58	13.33	125.32	18.67
L5 x T8	55.50	57.50	2.00**	79.37	139.39	31.28	11.88	15.58	13.62	27.32	14.26	121.79	18.32
L6 x T1	51.50	55.00	3.50	79.58	161.17	33.08	14.34	14.00	12.00	31.78	14.00	119.51	18.00
L6 x T2	51.00	55.00	4.00	67.17	153.75	36.92*	14.67	15.58	11.77	28.08	14.50	85.85	12.92
L6 xT3	50.50	54.50	4.00	49.00	102.33	37.17**	21.50**	15.33	12.40	27.98	13.84	111.21	17.00
L6 x T4	50.50	53.00**	2.50*	82.67	159.00	30.92	16.67**	16.87	11.92	37.83**	14.00	129.38	19.50
L6 x T5	52.00	56.00	4.00	66.92	146.58	31.00	15.83*	15.50	13.08	33.67	13.67	119.91	18.88
L6 x T6	51.00	55.00	4.00	123.83**	201.92**	27.07	16.67**	15.83	12.60	30.80	12.33	141.40	20.67
L6 x T7	52.00	56.00	4.00	95.25*	189.42**	33.00	17.83**	18.20**	13.33	37.83**	15.17	164.45**	26.92**
L6 x T8	52.50	57.00	4.50	81.50	158.17	32.98	15.67	15.08	12.45	28.00	14.33	93.87	14.00
L7 x T1	50.00**	53.50*	3.50	93.75	196.17**	44.92**	22.17**	17.97**	12.33	37.53**	17.00**	203.16**	22.67
L7 x T2	52.50	57.00	4.50	83.50	152.58	27.67	16.83**	16.11	13.33	32.33	13.67	134.21	14.67
L7 xT3	52.50	56.00	3.50	92.58	184.17**	32.97	12.83	19.08**	13.83	33.17	15.34	156.42**	25.83**
L7 x T4	52.00	56.00	4.00	96.85*	171.10	35.02	13.92	17.11	15.02**	33.51	14.20	150.17**	24.13**
L7 x T5	53.50	55.50	2.00**	82.67	162.47	25.14	12.37	14.21	13.53	31.55	14.95	138.78	23.17
L7 x T6	52.00	56.00	4.00	101.00**	181.35**	30.85	13.67	14.75	14.16	33.33	13.67	157.39*	21.17
L7 x T7	51.50	55.00	3.50	101.50**	181.58**	30.25	12.67	18.48**	13.03	31.50	12.67	156.95**	24.42**
L7 x T8	51.50	53.50*	2.00**	76.92	160.00	31.38	18.50**	16.37	11.53	26.50	12.00	116.86	15.67
L8 x T1	48.50**	51.50**	3.00	88.83	166.83	30.27	10.17	16.43	13.45	30.33	14.33	124.84	16.08
L8 x T2	52.00	55.50	3.50	86.58	149.35	32.30	14.67	15.00	11.57	36.50**	13.33	120.19	22.33
L8 xT3	54.50	57.50	3.00	90.00	159.50	37.00	16.50**	16.55	12.43	38.17**	14.00	150.96**	25.92**
L8 x T4	50.00**	54.50	4.50	66.17	136.62	28.42	15.33	15.42	13.00	31.37	16.33**	127.32	15.50
L8 x T5	51.00	55.00	4.00	93.17	159.08	32.00	19.00**	16.22	12.95	37.67**	14.67	160.42**	22.58
L8 x T6	52.50	56.50	4.00	99.75**	166.17	32.67	15.00	17.52*	14.60*	35.99**	16.00**	215.98**	24.33**
L8 x T7	51.00	54.50	3.50	82.83	149.00	29.83	14.83	16.17	12.67	35.17**	14.67	136.34	20.61
L8 x T8	47.50**	51.50**	4.00	85.50	153.50	37.48**	14.00	19.42**	12.83	36.17**	13.33	141.04	25.17**
L9 x T1	50.00**	53.00**	3.00	87.50	164.17	31.70	14.67	15.72	12.22	33.83	14.17	134.95	18.83
L9 x T2	44.00**	46.50**	2.50*	92.17	165.08	34.33	17.67**	15.60	11.50	37.06**	15.83**	117.28	17.58
L9 xT3	48.50**	51.50**	3.00	80.28	154.10	37.78**	12.50	15.65	12.52	36.84**	14.33	158.87**	18.00
L9 x T4	48.50**	52.50**	4.00	61.50	127.00	35.53	14.50	15.75	12.10	31.67	12.67	108.23	23.25*
L9 x T5	50.00**	53.00**	3.00	89.83	171.67	31.25	18.00**	19.00**	12.33	37.61**	16.33**	165.81**	21.83
L9 x T6	52.50	55.00	2.50*	88.67	160.92	28.67	12.33	19.35**	13.42	22.58	12.34	109.00	14.98
L9 x T7	49.50**	53.50*	4.00	59.42	116.39	28.33	16.50**	17.00	12.50	26.35	14.00	121.02	24.75**
L9 x T8	53.00	55.00	2.00**	66.12	113.38	31.00	12.22	10.74	8.94	24.84	16.34**	134.83	25.13**
Overall mean	**51.44**	**54.96**	**3.52**	**85.49**	**166.51**	**33.16**	**14.22**	**16.36**	**12.94**	**31.67**	**14.38**	**138.80**	**20.69**
CD at 5 %	1.0162	1.1274	0.8537	8.67	8.651	2.8846	1.467	1.1516	1.3385	2.175	1.0415	6.6069	2.1808
CD at 1 %	1.35	1.50	1.13	11.51	11.49	3.83	1.95	1.53	1.78	2.89	1.38	8.78	2.90

Table 20. Cont.,

	SH%	SPY	100 SW	SL	SG	ST	SVI	STR	HIP	TRP	PA
L1 x T1	76.16	104.43	26.70	0.83	0.60	0.33	3913.46	76.43**	1.47**	0.07**	5.24**
L1 x T2	86.23	95.01	27.50**	1.00**	0.67	0.33	3407.87	64.85	1.11	0.06**	7.95
L1 xT3	83.38	69.39	23.33	0.89	0.70	0.40**	3171.67	75.27**	0.89	0.05	11.91
L1 x T4	83.47	132.45**	27.77**	0.78	0.73**	0.40**	2614.53	75.73**	1.95**	0.04	4.46**
L1 x T5	84.95	138.29**	29.07**	0.98**	0.76**	0.33	4455.71	64.55	1.54**	0.05	5.29**
L1 x T6	81.30	114.90	32.10**	0.87	0.72*	0.37*	4376.92	73.35**	0.91	0.04	10.27
L1 x T7	83.10	165.92**	34.37**	0.99**	0.77**	0.40**	3921.27	70.68	0.86	0.04	7.94
L1 x T8	81.66	142.52**	26.58	0.70	0.50	0.30	4955.33**	67.25	0.82	0.05	12.48
L2 x T1	88.98**	143.51**	27.75**	0.92	0.53	0.42**	5123.40**	67.75	0.94	0.05	9.25
L2 x T2	87.07*	94.74	24.50	0.93	0.62	0.25	4604.54**	76.12**	0.83	0.05	9.99
L2 x T3	85.47	99.03	26.71	0.67	0.50	0.40**	4243.79	70.03	0.92	0.06**	8.81
L2 x T4	88.54**	134.53**	26.92	0.82	0.77**	0.30	4156.81	70.68	0.99	0.07**	8.68
L2 x T5	82.82	115.72	27.75**	0.87	0.77**	0.40**	4048.86	72.68*	0.93	0.05	8.45
L2 x T6	87.31*	165.07**	35.94**	0.97**	0.87**	0.47**	4535.13**	61.73	0.79	0.06**	9.92
L2 x T7	84.42	133.61**	26.27	1.00**	0.55	0.30	4792.52**	75.66**	0.98	0.06**	8.57
L2 x T8	87.10*	106.49	24.73	0.77	0.70	0.47**	4136.87	59.69	1.25**	0.06**	7.31*
L3 x T1	89.42**	151.48**	28.00**	0.93	0.83**	0.43**	4086.49	75.19**	0.82	0.08**	9.02
L3 x T2	84.43	112.81	26.77	0.92	0.70	0.37*	4076.28	63.20	1.20**	0.05	5.29**
L3 xT3	83.34	105.81	27.00	1.00**	0.68	0.49**	4372.38	77.26**	0.90	0.05	7.97
L3 x T4	88.61**	171.23**	27.80**	0.84	0.78**	0.40**	4141.11	75.73**	0.92	0.05	8.12
L3 x T5	83.72	85.69	24.08	0.73	0.50	0.30	3962.21	64.81	0.90	0.05	8.55
L3 x T6	83.12	154.50**	28.81**	0.92	0.85**	0.47**	4237.61	71.67	0.79	0.06**	9.87
L3 x T7	87.19*	131.00**	32.82**	1.00**	0.63	0.30	4210.17	68.63	0.97	0.07**	8.34
L3 x T8	85.68	130.62**	27.13*	0.80	0.77**	0.40**	4121.08	69.14	1.08	0.06**	8.32
L4 x T1	80.89	79.57	26.44	0.92	0.65	0.32	3790.47	72.00*	0.85	0.04	9.75
L4 x T2	84.27	109.49	22.34	0.65	0.68	0.25	3934.87	70.24	0.93	0.05	8.54
L4 xT3	87.18*	111.26	20.24	0.95*	0.62	0.20	4225.59	60.78	1.24**	0.05	7.52
L4 x T4	85.84	151.57**	23.84	1.00**	0.50	0.23	3329.19	76.92**	0.87	0.06**	9.69
L4 x T5	82.83	80.42	22.50	0.77	0.80**	0.30	4512.23*	64.73	1.02	0.05	8.16
L4 x T6	87.44*	127.47**	25.82	0.98**	0.73**	0.35	4583.57**	75.15**	0.98	0.05	8.72
L4 x T7	83.13	100.23	19.94	0.83	0.63	0.30	4329.34	62.04	1.38**	0.05	7.70
L4 x T8	85.19	115.02	25.36	0.70	0.47	0.27	4188.92	76.83**	0.94	0.05	7.64
L5 x T1	84.75	94.45	21.80	0.87	0.50	0.30	3333.70	72.05	0.92	0.04	8.53
L5 x T2	81.38	89.62	27.96**	0.87	0.73**	0.30	3021.76	73.42**	0.91	0.07**	8.16
L5 xT3	86.19	109.79	23.77	0.85	0.63	0.32	3484.41	60.32	1.25**	0.04	7.40*
L5 x T4	88.93**	117.92	24.56	0.97**	0.77**	0.33	3179.09	73.39**	0.76	0.06**	9.05

L5 x T5	88.89**	114.92	24.22	0.90	0.50	0.30	3901.23	73.45**	0.85	0.05	8.79
L5 x T6	88.09**	138.65**	32.73**	1.03**	0.83**	0.33	3963.82	65.58	1.34**	0.06**	6.26**
L5 x T7	85.10	106.65	28.00**	1.00**	0.53	0.33	4595.41**	62.69	0.94	0.04	9.36
L5 x T8	84.96	103.48	26.58	0.70	0.50	0.30	3032.15	71.19	0.70	0.06**	11.93
L6 x T1	84.93	101.51	22.81	0.93	0.80**	0.37*	3471.65	68.19	0.90	0.04	9.10
L6 x T2	84.96	72.93	17.92	0.97**	0.72*	0.30	3363.46	61.71	1.00	0.05	8.64
L6 xT3	84.71	94.21	24.34	0.83	0.57	0.30	4420.82	55.43	1.24**	0.04	6.43**
L6 x T4	84.94	109.88	20.75	0.88	0.47	0.40**	5006.50**	61.69	0.95	0.04	8.62
L6 x T5	84.27	101.04	21.97	0.88	0.68	0.42**	4234.16	77.68**	0.92	0.06**	8.53
L6 x T6	85.38	120.73	31.80**	0.83	0.57	0.30	3231.02	60.17	1.43**	0.07**	7.23**
L6 x T7	83.63	137.54**	23.98	0.95*	0.65	0.40**	4827.83**	72.78*	0.97	0.09**	9.62
L6 x T8	85.09	79.87	19.91	0.82	0.53	0.32	5396.56**	73.13**	0.99	0.07**	8.23
L7 x T1	88.84**	180.49**	23.43	0.83	0.57	0.50**	5171.61**	71.65	1.02	0.04	8.54
L7 x T2	89.07**	119.54	27.08*	0.93	0.80**	0.32	5475.80**	63.57	0.88	0.06**	9.41
L7 xT3	83.48	130.59**	25.70	0.80	0.70	0.32	5059.69**	68.09	1.28**	0.06**	5.30**
L7 x T4	83.93	126.04*	26.50	0.70	0.40	0.30	3123.54	70.09	0.86	0.06**	9.17
L7 x T5	83.31	115.61	24.52	0.80	0.50	0.30	5372.94**	71.68	0.83	0.04	10.23
L7 x T6	86.55	136.22**	29.92**	1.01**	0.90**	0.40**	4924.75**	71.04	0.86	0.04	8.68
L7 x T7	84.44	132.54**	33.22**	0.90	0.70	0.28	4734.11**	60.74	1.11	0.05	7.95
L7 x T8	86.59	101.19	31.82**	0.83	0.67	0.27	4018.42	67.10	1.29**	0.04	7.10**
L8 x T1	87.12*	108.76	25.11	0.95*	0.82**	0.32	2771.20	55.09	1.18*	0.07**	6.73**
L8 x T2	81.42	97.86	20.14	0.87	0.70	0.37**	3842.82	72.17*	0.96	0.04	8.53
L8 xT3	82.83	125.04*	23.43	0.88	0.53	0.32	5842.35*	56.77	1.23**	0.04	5.96**
L8 x T4	87.83**	111.82	21.83	0.85	0.57	0.40**	3935.22	67.08	0.94	0.06**	8.08
L8 x T5	85.92	137.83**	25.00	0.85	0.60	0.30	4680.20**	65.43	0.80	0.06**	7.93
L8 x T6	88.73**	191.66**	32.86**	1.00**	0.83**	0.47**	5224.45**	76.72**	1.12	0.04	8.46
L8 x T7	84.89	115.74	22.53	0.92	0.82**	0.40**	4567.49**	69.53	0.88	0.05	8.75
L8 x T8	82.14	115.87	24.04	0.75	0.60	0.30	5029.01**	76.83**	0.78	0.05	10.10
L9 x T1	86.07	116.12	24.23	0.87	0.55	0.32	3770.32	60.01	0.93	0.06**	4.95**
L9 x T2	84.96	99.70	16.97	0.73	0.72*	0.33	3657.69	71.86	1.17*	0.04	5.61**
L9 xT3	88.67**	140.87**	26.69	0.90	0.62	0.30	5131.53**	72.71*	0.98	0.05	7.37
L9 x T4	78.52	84.98	21.21	0.80	0.52	0.28	4238.30	75.94**	1.09	0.06**	6.31**
L9 x T5	86.83	143.98**	23.45	0.80	0.57	0.30	4860.47**	71.54	0.73	0.05	7.94
L9 x T6	86.24	94.02	33.80**	0.97**	0.72*	0.30	3695.09	77.45**	1.07	0.05	6.49**
L9 x T7	79.55	96.27	26.10	1.03**	0.70	0.30	4640.97**	77.03**	1.01	0.05	8.04
L9 x T8	81.37	109.71	27.04*	0.80	0.70	0.30	3548.59	65.26	0.94	0.05	9.60
Overall mean	**85.00**	**118.18**	**25.92**	**0.87**	**0.66**	**0.34**	**4199.17**	**69.23**	**1.01**	**0.05**	**8.23**
CD at 5 %	1.8659	6.0526	1.0966	0.0757	0.0528	0.0378	246.80	2.75	0.1318	0.009	0.7525
CD at 1 %	2.48	8.04	1.46	0.10	0.07	0.05	327.82	3.66	0.18	0.01	1.00

Table 21. *sca* of the 72 hybrids in Environment -3

Code No	50 DT	50 DS	ASI	CPH	PH	TL	TBR	CL	CG	NKr/R	NR/C	CW	SHW
L1 x T1	0.67	1.16 **	0.49	18.68 **	11.60 **	-3.34 **	2.43 **	2.08 **	-0.57	-1.20	-0.72	-11.52 **	7.57 **
L1 x T2	-3.33 **	-2.78 **	0.54	-10.67 **	-12.65 **	-1.28	0.06	-0.61	-0.30	-4.57 **	0.35	-15.12 **	-7.57 **
L1 xT3	0.28	0.49	0.21	-1.52	-19.95 **	0.40	0.62	-1.46 **	0.68	-5.62 **	-1.60 **	-54.11 **	-10.47 **
L1 x T4	0.51	0.16	-0.35	13.06 **	39.58 **	-3.73 **	-0.99	0.25	1.16 *	0.69	0.70	12.96 **	1.40
L1 x T5	0.84 *	1.60 **	0.76 *	-1.26	-9.25 **	-6.16 **	-2.93 **	2.80 **	-0.65	4.36 **	-0.48	19.06 **	-0.92
L1 x T6	-1.10 **	-1.40 **	-0.29	-14.52 **	-8.56 **	7.21 **	-0.69	-1.59 **	-0.50	-4.22 **	0.35	-27.65 **	-0.94
L1 x T7	0.45	-0.84	-1.29 **	-1.77	-1.44	4.14 **	-0.23	1.05 *	-0.83	4.04 **	0.15	42.98 **	4.92 **
L1 x T8	1.67 **	1.60 **	-0.07	-1.99	0.66	2.75 *	1.72 **	-2.53 **	1.01 *	6.52 **	1.24 **	33.40 **	6.00 **
L2 x T1	1.11 **	2.41 **	1.30 **	-0.96	-8.06 *	2.51 *	1.50 **	-1.34 **	0.10	3.47 **	0.33	16.67 **	-0.83
L2 x T2	0.61	0.47	-0.15	17.77 **	25.27 **	4.72 **	3.12 **	1.66 **	1.10 *	-3.07 **	0.24	-12.47 **	-2.16 **
L2 x T3	3.72 **	3.74 **	0.02	-22.17 **	-45.98 **	-18.63 **	-7.72 **	-1.95 **	-1.43 **	-3.07 **	-0.88 *	-17.46 **	-0.96
L2 x T4	0.44	-0.09	-0.53	-3.58	-11.52 **	0.86	-0.09	0.33	-0.93	1.61	-0.91 *	-1.54	-2.85 **
L2 x T5	0.78	0.35	-0.42	17.48 **	13.74 **	2.41 *	0.88	0.55	-0.39	-2.53 **	-0.09	-0.01	5.08 **
L2 x T6	-1.17 **	-1.15 **	0.02	-14.91 **	-7.53 *	6.58 **	-0.29	0.57	0.64	2.28 **	-0.09	24.10 **	3.14 **
L2 x T7	-3.11 **	-3.09 **	0.02	13.84 **	37.31 **	-0.98	1.33 *	-1.24 **	0.41	2.13 *	0.87 *	5.58 *	2.33 **
L2 x T8	-2.39 **	-2.65 **	-0.26	-7.47 *	-3.23	2.53 *	1.27 *	1.42 **	0.50	-0.82	0.53	-14.87 **	-3.75 **
L3 x T1	0.92 *	-0.09	-1.01 **	12.71 **	9.71 **	-7.74 **	-2.44 **	2.31 **	-0.50	4.65 **	0.10	16.41 **	-3.38 **
L3 x T2	-0.08	-0.53	-0.46	-7.64 *	-16.96 **	-4.42 **	2.70 **	-0.59	-2.27 **	-0.06	-0.49	3.96	1.88 *
L3 xT3	-0.97 *	-1.26 **	-0.29	30.50 **	49.53 **	4.52 **	2.86 **	-0.88 *	-0.37	-5.65 **	0.76	-14.74 **	0.66
L3 x T4	0.76	0.41	-0.35	-16.99 **	-29.17 **	1.58	-1.68 **	1.05 *	3.76 **	3.23 **	0.69	31.44 **	4.11 **
L3 x T5	0.09	-0.15	-0.24	-1.02	4.27	-0.71	0.45	-2.47 **	-0.94	-7.23 **	-0.32	-45.76 **	-4.97 **
L3 x T6	2.15 **	2.35 **	0.21	-2.32	-3.13	-2.71 *	-2.39 **	0.12	-0.25	5.26 **	0.51	12.58 **	7.87 **
L3 x T7	-1.80 **	-1.09 *	0.71 *	-10.73 **	-18.84 **	5.53 **	1.90 **	-0.77	-0.20	-3.12 **	-1.20 **	-10.84 **	-5.80 **
L3 x T8	-1.08 **	0.35	1.43 **	-4.51	4.58	3.95 **	-1.40 **	1.22 **	0.78	2.91 **	-0.05	6.95 **	-0.38
L4 x T1	-4.45 **	-2.59 **	1.86 **	-3.36	-0.83	2.40 *	0.34	1.07 **	0.06	-8.62 **	-1.87 **	-31.67 **	0.05
L4 x T2	-2.45 **	-2.53 **	-0.08	-10.38 **	-15.83 **	-2.77 *	-2.86 **	0.43	-0.18	2.01 *	0.53	23.17 **	4.02 **
L4 xT3	1.16 **	1.24 **	0.08	-15.23 **	-14.74 **	-2.59 *	-1.71 **	1.05 *	-0.39	5.50 **	0.25	8.81 **	-1.63 *
L4 x T4	1.38 **	0.91 *	-0.47	19.67 **	15.11 **	-0.30	-0.08	1.21 **	0.63	1.60	2.21 **	37.62 **	4.56 **
L4 x T5	2.72 **	1.35 **	-1.36 **	-15.32 **	-21.48 **	2.07	1.72 **	-2.51 **	0.06	-5.04 **	-1.63 **	-28.10 **	-2.41 **
L4 x T6	2.27 **	2.35 **	0.08	8.01 *	7.89 *	-1.89	1.88 **	-0.60	-0.02	3.29 **	-0.14	-4.66	-2.73 **
L4 x T7	0.83 *	-0.09	-0.92 **	5.95	12.08 **	2.62 *	1.00	-0.46	-0.40	0.78	0.66	-17.60 **	-2.18 **
L4 x T8	-1.45 **	-0.65	0.81 *	10.67 **	17.79 **	0.45	-0.30	-0.19	0.24	0.47	-0.01	12.43 **	0.32
L5 x T1	3.24 **	2.28 **	-0.95 **	-21.17 **	-23.60 **	-2.77 *	1.48 **	-0.48	0.05	-1.22	0.92 *	-16.68 **	0.23
L5 x T2	1.24 **	1.34 **	0.10	11.81 **	24.57 **	2.65 *	-4.06 **	-1.15 **	0.25	-2.92 **	-1.34 **	5.32 *	6.09 **
L5 xT3	-5.15 **	-4.88 **	0.27	-3.04	-1.44	-2.66 *	1.44 **	-0.79	-0.58	2.74 **	0.38	10.54 **	1.62 *
L5 x T4	-1.93 **	-1.22 **	0.72 *	-0.95	-5.39	-0.07	2.06 **	-0.51	-1.92 **	1.50	-0.65	-4.41	-3.76 **
L5 x T5	-2.10 **	-0.27	1.83 **	-0.65	5.34	2.46 *	2.45 **	-0.03	0.52	0.22	1.51 **	6.02 *	-2.75 **
L5 x T6	-1.54 **	-2.27 **	-0.73 *	6.88 *	4.15	2.30 *	1.61 **	1.22 **	-0.55	0.81	0.33	8.93 **	-0.27

L5 x T7	2.01 **	2.28 **	0.27	-1.61	2.87	1.44	-3.18 **	0.49	1.18 *	-1.07	-0.88 *	-10.89 **	-1.83 *
L5 x T8	4.24 **	2.73 **	-1.51 **	8.73 **	-6.50 *	-3.35 **	-1.79 **	1.24 **	1.05 *	-0.06	-0.28	1.17	0.65
L6 x T1	0.67	0.16	-0.51	-5.90	-8.59 **	0.02	-3.04 **	-2.43 **	-0.06	-0.12	-0.23	-2.40	0.22
L6 x T2	1.67 **	1.72 **	0.04	-11.99 **	-3.01	4.09 **	-2.24 **	0.58	0.30	-2.52 **	0.68	-12.74 **	-2.50 **
L6 xT3	-1.22 **	-1.01 *	0.21	-22.68 **	-40.01 **	5.38 **	5.59 **	0.20	0.35	-3.80 **	0.06	0.59	0.04
L6 x T4	-0.49	-1.84 **	-1.35 **	6.49 *	7.95 *	-1.44	0.54	0.52	-1.55 **	2.82 **	-0.48	-1.41	0.07
L6 x T5	-0.66	-0.40	0.26	-12.62 **	-11.99 **	0.83	-2.32 **	0.20	0.69	1.83 *	-0.65	2.88	0.78
L6 x T6	-1.60 **	-1.40 **	0.21	28.15 **	25.66 **	-5.92 **	1.01	-0.45	-0.70	-0.99	-1.16 **	-0.86	0.64
L6 x T7	0.95 *	1.16 **	0.21	12.50 **	21.87 **	-0.84	0.97	1.48 **	0.63	5.17 **	1.47 **	34.46 **	5.42 **
L6 x T8	0.67	1.60 **	0.93 **	6.05 *	8.11 *	-2.11	-0.51	-0.10	0.34	-2.39 **	0.31	-20.54 **	-4.67 **
L7 x T1	-1.39 **	-1.47 **	-0.08	-2.08	11.78 **	12.35 **	6.07 **	0.58	-0.63	5.19 **	2.56 **	50.21 **	1.91 *
L7 x T2	2.61 **	3.59 **	0.98 **	-6.02	-18.81 **	-4.67 **	1.20 *	0.15	0.97 *	1.29	-0.37	4.58	-3.72 **
L7 xT3	0.22	0.37	0.15	10.54 **	27.19 **	1.67	-1.80 **	2.99 **	0.89	0.96	1.36 **	14.76 **	5.89 **
L7 x T4	0.44	1.03 *	0.59	10.31 **	5.42	3.15 **	-0.92	-0.21	0.66	-1.94 *	-0.48	-11.66 **	1.72 *
L7 x T5	0.28	-1.02 *	-1.30 **	-7.23 *	-10.74 **	-4.54 **	-4.51 **	-2.05 **	0.22	-0.71	0.42	-9.29 **	2.10 **
L7 x T6	-1.17 **	-0.52	0.65 *	-5.03	-9.54 **	-1.64	-0.71	-2.49 **	-0.05	1.11	-0.03	-15.91 **	-1.84 *
L7 x T7	-0.11	0.03	0.15	8.39 **	-0.61	-3.10 **	-2.93 **	0.80 *	-0.57	-1.59	-1.23 **	-4.09	-0.07
L7 x T8	-0.89 *	-2.02 **	-1.13 **	-8.89 **	-4.69	-3.22 **	3.60 **	0.23	-1.48 **	-4.32 **	-2.23 **	-28.60 **	-5.98 **
L8 x T1	-1.83 **	-2.72 **	-0.89 **	-2.51	1.11	-2.52 *	-5.49 **	-0.78	0.89	-4.74 **	-0.50	-23.51 **	-4.77 **
L8 x T2	3.17 **	2.84 **	-0.33	1.56	-3.37	-0.26	-0.53	-0.78	-0.40	2.72 **	-1.09 **	-4.83	3.84 **
L8 xT3	3.28 **	2.62 **	-0.67 *	12.45 **	21.19 **	5.48 **	2.29 **	0.63	-0.11	3.22 **	-0.38	13.90 **	5.88 **
L8 x T4	-0.49	0.28	0.78 *	-15.88 **	-10.39 **	-3.68 **	0.92	-1.73 **	-0.96	-6.82 **	1.26 **	-29.91 **	-7.01 **
L8 x T5	-1.16 **	-0.77	0.39	7.77 *	4.55	2.10	2.56 **	0.12	0.06	2.66 **	-0.26	16.96 **	1.41
L8 x T6	0.40	0.73	0.33	-1.79	-6.06	-0.05	1.05 *	0.45	0.80	1.02	1.91 **	47.28 **	1.22
L8 x T7	0.45	0.28	-0.17	-5.78	-14.52 **	-3.74 **	-0.33	-1.35 **	-0.52	-0.67	0.37	-20.08 **	-3.98 **
L8 x T8	-3.83 **	-3.27 **	0.56	4.19	7.49 *	2.66 *	-0.47	3.44 **	0.23	2.60 **	-1.30 **	0.19	3.41 **
L9 x T1	1.05 **	0.85 *	-0.20	4.58	6.87 *	-0.92	-0.86	-1.01 *	0.66	2.58 **	-0.59	2.49	-1.00
L9 x T2	-3.45 **	-4.10 **	-0.65 *	15.56 **	20.78 **	1.95	2.61 **	0.31	0.54	7.11 **	1.49 **	8.14 **	0.11
L9 xT3	-1.34 **	-1.32 **	0.02	11.15 **	24.21 **	6.44 **	-1.57 **	0.22	0.97 *	5.71 **	0.04	37.70 **	-1.02
L9 x T4	-0.62	0.35	0.97 **	-12.12 **	-11.59 **	3.61 **	0.23	-0.91 *	-0.86	-2.70 **	-2.33 **	-33.10 **	1.76 *
L9 x T5	-0.78 *	-0.71	0.08	12.85 **	25.55 **	1.53	1.70 **	3.40 **	0.44	6.43 **	1.50 **	38.24 **	1.68 *
L9 x T6	1.77 **	1.29 **	-0.48	-4.46	-2.89	-3.88 **	-1.48 **	2.77 **	0.62	-8.56 **	-1.68 **	-43.81 **	-7.10 **
L9 x T7	0.33	1.35 **	1.02 **	-20.79 **	-38.71 **	-5.07 **	1.48 **	-0.02	0.31	-5.66 **	-0.22	-19.52 **	1.19
L9 x T8	3.05 **	2.29 **	-0.76 *	-6.77 *	-24.22 **	-3.65 **	-2.11 **	-4.74 **	-2.67 **	-4.91 **	1.79 **	9.87 **	4.39 **

Table 21. Cont.,

	SH%	SPY	100 SW	SL	SG	ST	SVI	STR	HIP	TRP	PA
L1 x T1	-6.61 **	-19.02 **	-0.95 *	-0.07 *	-0.07 **	-0.05 **	323.61 **	5.47	0.29 **	0.01 **	-2.62 **
L1 x T2	3.83 **	-7.48 **	1.53 **	0.12 **	-0.06 **	0.00	-176.73	-5.97	-0.07	0.01 **	-0.02
L1 xT3	0.82	-43.57 **	-3.75 **	0.02	0.06 **	0.05 **	-920.39 **	6.72	-0.39 **	0.00	4.32 **
L1 x T4	0.31	11.07 **	0.69	-0.07 *	0.10 **	0.05 **	-785.54 **	1.56	0.73 **	-0.01 **	-3.52 **
L1 x T5	2.58 **	20.04 **	1.84 **	0.14 **	0.11 **	-0.02	355.23 **	-7.32	0.42 **	0.01	-2.88 **
L1 x T6	-2.25 **	-26.64 **	-1.94 **	-0.09 **	-0.09 **	-0.04 **	415.96 **	0.78	-0.30 **	-0.01 *	1.88 **
L1 x T7	1.62 **	38.12 **	4.40 **	0.02	0.08 **	0.05 **	-244.89 *	3.37	-0.33 **	-0.01 **	-0.49
L1 x T8	-0.29	27.47 **	-1.83 **	-0.07 *	-0.13 **	-0.04 **	1032.75 **	-4.60	-0.34 **	0.00	3.33 **
L2 x T1	2.28 **	17.58 **	0.96 *	0.03	-0.13 **	0.01	930.40 **	2.79	-0.00	-0.01 **	0.71 **
L2 x T2	0.74	-10.24 **	-0.61	0.06 *	-0.09 **	-0.10 **	416.79 **	11.30 **	-0.11 *	-0.00	1.34 **
L2 x T3	-1.02	-16.43 **	0.48	-0.19 **	-0.12 **	0.03 *	-451.41 **	7.49	-0.13 *	0.01 *	0.54 *
L2 x T4	1.45 *	0.82	0.70	-0.02	0.15 **	-0.07 **	153.60	2.52	0.01	0.01 **	0.02
L2 x T5	-3.48 **	-5.02 *	1.38 **	0.03	0.13 **	0.04 **	-654.76 **	6.81	0.04	-0.01	-0.39
L2 x T6	-0.18	21.03 **	2.76 **	0.02	0.08 **	0.05 **	-28.98	-4.84	-0.19 **	0.00	0.84 **
L2 x T7	-0.99	3.32	-2.85 **	0.05	-0.12 **	-0.07 **	23.22	-19.90 **	0.03	-0.00	-0.54 *
L2 x T8	1.21 *	-11.05 **	-2.82 **	0.01	0.09 **	0.11 **	-388.86 **	-6.17	0.34 **	-0.00	-2.52 **
L3 x T1	3.48 **	19.24 **	0.98 *	0.02	0.12 **	0.01	197.82 *	4.54	-0.12 *	0.02 **	1.17 **
L3 x T2	-1.13 *	1.52	1.43 **	0.03	-0.07 **	0.00	192.86 *	-7.32	0.27 **	-0.01 **	-2.68 **
L3 xT3	-2.38 **	-15.95 **	0.55	0.12 **	0.01	0.09 **	-18.51	9.02 *	-0.14 *	-0.01	0.39
L3 x T4	2.29 **	31.22 **	1.34 **	-0.03	0.11 **	0.01	442.22 **	1.87	-0.05	-0.01 **	0.15
L3 x T5	-1.81 **	-41.35 **	-2.53 **	-0.13 **	-0.19 **	-0.08 **	-437.09 **	-6.75	0.02	-0.01 *	0.39
L3 x T6	-3.59 **	4.16	-4.60 **	-0.05	0.01	0.03 *	-22.17	-0.59	-0.17 **	0.00	1.49 **
L3 x T7	2.56 **	-5.60 *	3.48 **	0.03	-0.09 **	-0.09 **	-254.81 **	1.63	0.02	0.01 **	-0.08
L3 x T8	0.56	6.77 **	-0.66	0.02	0.10 **	0.02	-100.32	-2.40	0.17 **	-0.00	-0.82 **
L4 x T1	-3.95 **	-31.66 **	3.91 **	0.05	0.02	0.01	-59.05	2.22	-0.17 **	-0.01 **	1.62 **
L4 x T2	-0.20	19.22 **	1.49 **	-0.20 **	0.00	-0.00	90.59	0.59	-0.08	0.00	0.30
L4 xT3	2.55 **	10.51 **	-1.73 **	0.11 **	0.02	-0.07 **	-126.15	-6.59	0.12 *	0.00	-0.34
L4 x T4	0.62	32.57 **	1.88 **	0.18 **	-0.09 **	-0.04 **	-330.55 **	3.93	-0.18 **	0.01 *	1.44 **
L4 x T5	-1.60 **	-25.61 **	0.39	-0.05	0.19 **	0.04 **	152.07	-5.96	0.06	0.00	-0.28
L4 x T6	1.83 **	-1.86	-3.09 **	0.06 *	-0.03	0.03 *	362.92 **	3.76	-0.06	0.00	0.05
L4 x T7	-0.40	-15.35 **	-4.92 **	-0.10 **	-0.01	0.03 *	-96.49	-4.09	0.36 **	-0.01 *	-1.01 **
L4 x T8	1.17 *	12.18 **	2.06 **	-0.04	-0.11 **	0.01	6.66	6.15	-0.05	-0.00	-1.78 **
L5 x T1	-1.53 **	-16.84 **	-3.62 **	-0.05	-0.12 **	-0.04 **	32.01	3.09	-0.03	-0.01 **	0.18
L5 x T2	-4.52 **	-0.71	4.21 **	-0.03	0.06 **	0.01	-274.69 **	4.60	-0.04	0.02 **	-0.30
L5 xT3	0.12	8.98 **	-1.08 **	-0.04	0.05 **	0.01	-319.50 **	-6.23	0.20 **	-0.01 **	-0.68 *
L5 x T4	2.27 **	-1.14	-0.29	0.10 **	0.19 **	0.02	67.17	1.22	-0.22 **	0.01	0.58 *

L5 x T5	3.01 **	8.84 **	-0.78	0.03	-0.10 **	0.00	88.90	3.58	-0.04	0.00	0.13
L5 x T6	1.03	9.27 **	0.92 *	0.06 *	0.09 **	-0.03 *	291.00 **	-4.99	0.36 **	0.01 **	-2.62 **
L5 x T7	0.12	-8.99 **	0.25	0.02	-0.10 **	0.02	717.40 **	-2.62	-0.01	-0.01 **	0.44
L5 x T8	-0.49	0.59	0.39	-0.09 **	-0.07 **	0.00	-602.28 **	1.34	-0.22 **	0.00	2.29 **
L6 x T1	-0.05	-2.56	0.66	0.03	0.18 **	-0.00	-510.10 **	1.90	-0.14 **	-0.02 **	1.13 **
L6 x T2	0.35	-10.18 **	-2.55 **	0.08 **	0.04 *	-0.02	-613.04 **	-4.45	-0.03	-0.00	0.56 *
L6 xT3	-0.06	0.63	2.75 **	-0.05	-0.01	-0.05 **	-63.14	-8.45 *	0.10	-0.01 **	-1.27 **
L6 x T4	-0.43	-1.96	-0.84 *	0.02	-0.11 **	0.05 **	1214.53 **	-7.81	-0.12 *	-0.02 **	0.54 *
L6 x T5	-0.32	2.17	0.23	0.03	0.09 **	0.08 **	-258.23 **	10.48 *	-0.06	0.00	0.26
L6 x T6	-0.38	-1.43	3.26 **	-0.14 **	-0.18 **	-0.09 **	-1121.85 **	-7.73	0.36 **	0.01 **	-1.27 **
L6 x T7	-0.05	29.12 **	-0.50	-0.02	0.02	0.06 **	269.76 **	10.13 *	-0.08	0.03 **	1.08 **
L6 x T8	0.93	-15.79 **	-3.01 **	0.05	-0.04 *	-0.02	1082.07 **	5.94	-0.03	0.01 **	-1.03 **
L7 x T1	2.83 **	48.37 **	-3.56 **	-0.04	-0.08 **	0.14 **	698.75 **	3.71	0.02	-0.01 **	0.58 *
L7 x T2	3.43 **	8.37 **	1.76 **	0.08 **	0.10 **	0.01	1008.19 **	-4.24	-0.13 *	0.01 *	1.34 **
L7 xT3	-2.33 **	8.94 **	-0.73	-0.04	0.09 **	-0.02	84.61	2.56	0.17 **	0.01 **	-2.40 **
L7 x T4	-2.48 **	-13.86 **	0.08	-0.12 **	-0.21 **	-0.03 **	-1159.55 **	-1.06	-0.18 **	0.01 **	1.09 **
L7 x T5	-2.31 **	-11.31 **	-2.06 **	-0.02	-0.13 **	-0.02	389.45 **	2.83	-0.12 *	-0.01	1.96 **
L7 x T6	-0.26	-14.01 **	-3.46 **	0.08 **	0.12 **	0.02	80.77	1.48	-0.17 **	-0.01 *	0.19
L7 x T7	-0.27	-3.95	3.90 **	-0.03	0.04 *	-0.04 **	-315.06 **	-3.55	0.09	0.00	-0.59 *
L7 x T8	1.39 *	-22.54 **	4.06 **	0.09 **	0.07 **	-0.05 **	-787.17 **	-1.74	0.31 **	-0.00	-2.16 **
L8 x T1	1.77 **	-18.67 **	1.53 **	0.05	0.14 **	-0.07 **	-1453.15 **	-12.31 **	0.21 **	0.01 **	-1.00 **
L8 x T2	-3.56 **	-8.61 **	-1.77 **	-0.01	-0.03	0.04 **	-376.28 **	4.91	-0.01	-0.01 **	0.68 *
L8 xT3	-2.31 **	8.10 **	0.41	0.01	-0.11 **	-0.04 **	1115.80 **	-8.21 *	0.16 **	-0.00	-1.50 **
L8 x T4	2.09 **	-23.38 **	-1.19 **	-0.01	-0.07 **	0.04 **	-99.34	-3.53	-0.07	0.01	0.22
L8 x T5	0.97	15.61 **	1.83 **	-0.00	-0.06 **	-0.04 **	-54.78	-2.88	-0.12 *	0.01	-0.11
L8 x T6	2.60 **	46.13 **	2.89 **	0.04	0.03	0.07 **	628.99 **	7.71	0.11 *	-0.01 *	0.19
L8 x T7	0.83	-16.04 **	-3.38 **	-0.05	0.13 **	0.05 **	-233.17 *	5.78	-0.10 *	-0.00	0.44
L8 x T8	-2.39 **	-3.16	-0.31	-0.02	-0.03	-0.04 **	471.93 **	8.54 *	-0.17 **	-0.00	1.07 **
L9 x T1	1.80 **	3.56	0.08	-0.02	-0.08 **	-0.01	-160.30	-11.41 **	-0.05	0.01 **	-1.76 **
L9 x T2	1.07	8.10 **	-5.51 **	-0.13 **	0.03	0.06 **	-267.69 **	0.58	0.20 **	-0.01 **	-1.21 **
L9 xT3	4.61 **	38.80 **	3.10 **	0.05	0.02	-0.00	698.70 **	3.70	-0.10	0.00	0.94 **
L9 x T4	-6.13 **	-35.34 **	-2.38 **	-0.04	-0.07 **	-0.02	497.45 **	1.31	0.08	0.00	-0.51
L9 x T5	2.96 **	36.62 **	-0.29	-0.03	-0.04 *	0.01	419.21 **	-0.79	-0.20 **	0.00	0.93 **
L9 x T6	1.20 *	-36.64 **	3.26 **	0.02	-0.04 *	-0.05 **	-606.65 **	4.42	0.07	-0.00	-0.75 **
L9 x T7	-3.42 **	-20.64 **	-0.38	0.09 **	0.06 **	0.00	134.04	9.26 *	0.02	-0.00	0.76 **
L9 x T8	-2.08 **	5.55 *	2.12 **	0.05	0.12 **	0.01	-714.77 **	-7.06	-0.01	-0.00	1.61 **

Table 22. Mean performance of hybrids raised across the environments

Code No	50 DT	50 DS	ASI	CPH	PH	TL	TBR	CL	CG	NKr/R	NR/C	CW	SHW
L1 x T1	52.17	55.83	3.67	97.36**	177.11	29.64	15.48	17.72**	13.11	30.39	12.94	127.60	24.06**
L1 x T2	48.83**	52.17**	3.33	86.28	164.14	34.06	16.44*	16.71	13.35	30.68	14.16	132.40	19.28
L1 xT3	52.17	54.67	2.50**	90.23	155.37	29.46	17.84	15.92	13.20	25.98	13.33	103.08	19.02
L1 x T4	51.83	54.83	3.00**	91.36	185.59**	31.72	14.95	16.90	14.89**	32.78*	14.00	150.78**	27.01**
L1 x T5	52.00	56.17	4.17	92.14	174.75	29.22	16.24**	18.48**	12.89	32.14	13.28	162.76**	26.52**
L1 x T6	50.50	54.50	4.00	94.67*	183.87**	37.21**	12.17	16.96	13.55*	30.99	13.28	153.48**	24.92**
L1 x T7	50.50	53.33*	2.83**	92.74	176.03	33.90	12.78	18.18**	13.96**	32.52	13.22	171.83**	26.36**
L1 x T8	52.83	55.83	3.00**	74.60	145.23	33.51	12.50	13.62	12.66	27.01	14.70	121.97	22.15
L2 x T1	53.00	56.67	3.67	93.64	189.50**	32.96	14.11	16.81	12.49	35.89**	15.11**	174.82**	19.17
L2 x T2	50.17	53.67	3.50	103.58*	190.39**	32.18	15.28	16.77	12.38	30.84	14.50	137.72	17.83
L2 x T3	54.50	58.67	4.17	69.35	137.39	17.75	7.72	14.87	11.36	30.67	13.33	114.99	18.03
L2 x T4	53.00	56.33	3.33	94.75*	166.94	27.91	12.00	16.91	12.39	33.61**	14.67	167.76**	21.16
L2 x T5	54.67	58.00	3.33	90.17	168.65	31.08	15.94*	17.22	13.30	31.37	14.29	143.68*	19.91
L2 x T6	53.17	56.33	3.17*	99.06**	190.46**	32.34	12.92	16.66	13.38	33.24**	13.50	164.68**	20.70
L2 x T7	51.33	55.50	4.17	106.85**	207.59**	34.72*	11.83	16.76	12.88	30.89	14.39	158.72**	23.50**
L2 x T8	48.67**	52.67**	4.00	83.10	163.71	37.81**	12.61	16.04	11.77	25.98	13.89	125.23	17.89
L3 x T1	53.33	56.83	3.50	102.00**	186.67**	27.96	11.67	16.93	12.73	29.63	14.50	146.01**	20.51
L3 x T2	50.50	54.17	3.67	90.95	171.31	29.68	14.17	16.19	12.17	30.46	14.28	134.82	20.79
L3 xT3	51.50	54.17	2.67**	108.22**	201.11**	34.31*	13.95	16.19	12.85	28.25	14.12	137.68	21.11
L3 x T4	52.17	56.00	3.83	85.50	168.00	30.69	11.94	16.68	14.38	33.96**	15.05**	190.69**	25.97**
L3 x T5	51.50	55.50	4.00	91.95	184.49**	30.46	16.11**	15.18	12.73	29.80	15.06**	130.11	19.86
L3 x T6	54.67	58.50	3.83	98.78**	181.61**	29.13	10.45	17.08	13.38	27.89	14.33	152.15**	24.74**
L3 x T7	51.00	54.00	3.00**	89.74	180.47**	38.00**	14.01	17.94**	13.79**	27.94	13.71	138.80	19.35
L3 x T8	49.83*	54.33	4.50	98.25**	198.64**	36.69**	13.89	16.72	13.02	31.31	14.83	165.40**	22.16
L4 x T1	45.67**	51.33**	5.67	86.00	172.25	35.78**	14.42	16.51	12.29	27.83	13.89	122.15	19.72
L4 x T2	44.67**	48.83**	4.17	76.50	153.75	31.92	12.78	15.08	11.38	30.61	14.56	126.01	15.42
L4 xT3	50.67	54.83	4.17	74.39	149.03	30.72	14.33	14.48	11.18	33.16**	14.83	132.44	16.11
L4 x T4	51.67	55.33	3.67	98.47**	173.61	31.98	14.06	16.22	13.55*	30.76	16.22**	154.75**	22.86**
L4 x T5	53.17	55.67	2.50**	85.15	165.39	35.59**	16.97**	14.75	12.51	26.94	14.17	115.86	19.81
L4 x T6	54.83	58.83	4.00	105.47**	195.15**	33.66	14.45	16.32	13.72**	33.44**	14.39	160.09**	23.81**
L4 x T7	49.67**	53.67	4.00	99.97**	183.62**	33.87	14.03	17.87**	13.02	32.56	14.51	131.97	20.43
L4 x T8	49.33**	53.83	4.50	89.53	168.00	34.11	11.47	16.31	11.97	30.79	14.72	132.37	21.92
L5 x T1	52.33	56.00	3.67	74.11	150.61	30.11	14.72	16.28	12.58	28.56	14.61	120.66	18.87
L5 x T2	48.83**	53.00**	4.17	84.11	166.70	30.87	13.79	16.19	12.55	30.44	14.00	118.48	19.30
L5 xT3	46.50**	50.17**	3.67	74.17	138.42	33.45	13.40	15.07	12.26	32.05	14.28	126.20	17.90
L5 x T4	48.83*	53.67	4.83	81.53	152.00	36.22**	14.95	15.15	12.26	34.04**	14.50	132.73	18.64
L5 x T5	49.17*	54.33	5.17	86.53	159.92	30.92	14.08	14.63	12.50	29.42	14.67	120.68	15.45
L5 x T6	48.33**	52.17**	3.83	102.39**	175.50	33.29	14.42	16.41	13.53*	31.96	14.39	168.88**	24.83**

L5 x T7	53.00	57.33	4.33	78.19	151.59	29.72	12.69	15.21	12.51	29.78	13.78	126.52	19.25
L5 x T8	54.50	56.83	2.33**	90.28	141.92	26.43	12.54	14.54	12.55	30.05	12.86	106.81	18.70
L6 x T1	52.33	56.67	4.33	82.08	156.16	34.19	13.72	16.46	12.35	33.60**	13.95	112.51	17.28
L6 x T2	49.17*	52.00**	2.83**	73.85	149.75	36.25**	13.22	17.02	12.18	29.94	14.61	91.37	17.28
L6 xT3	49.17*	53.33**	4.17	66.69	135.67	36.45**	16.92**	16.63	12.77	28.12	13.22	117.43	20.03
L6 x T4	49.17*	52.67**	3.50	74.06	143.79	30.88	14.06	16.37	12.59	36.83**	15.33**	148.17**	20.06
L6 x T5	50.00	53.50*	3.50	77.07	142.49	27.14	14.29	15.95	12.14	32.29	13.46	118.10	18.25
L6 x T6	50.33	54.50	4.17	104.78**	179.42**	29.36	15.17	16.14	12.57	28.99	13.56	142.04	21.36
L6 x T7	50.00	54.33	4.33	89.64	179.58**	36.75**	15.22	16.98	13.69**	34.96**	14.67	157.31**	21.91
L6 x T8	49.67*	54.00	4.33	81.67	150.67	34.16	15.39	15.86	12.09	30.22	14.67	110.33	17.12
L7 x T1	49.33*	53.50*	4.17	89.14	171.55	42.08**	17.22**	17.14	12.29	38.11	17.06**	196.81**	21.86
L7 x T2	52.00	56.00	4.00	85.61	158.53	29.88	14.92	16.47	13.00	31.78*	14.78**	147.22*	18.20
L7 xT3	52.17	55.00	2.83**	96.81**	189.78**	32.96	12.44	16.92	13.21	32.70*	15.17**	153.58**	23.60**
L7 x T4	51.33	54.83	3.50	89.58	166.18	31.85	12.48	14.99	13.16	29.70	14.04	121.48	20.69
L7 x T5	52.50	55.17	2.67**	61.45	128.79	21.08	11.39	15.05	11.49	33.84**	15.26**	133.39	19.80
L7 x T6	51.83	55.50	3.67	100.06**	173.95	32.42	14.17	16.57	12.96	33.74**	14.00	154.44**	19.43
L7 x T7	51.17	54.50	3.33	92.22	196.64**	31.50	13.61	18.17**	13.51*	32.17	13.78	168.95**	25.44**
L7 x T8	50.17	52.50**	2.33**	87.75	168.56	34.63*	15.61*	17.24*	12.61	30.28	14.56	119.44	18.47
L8 x T1	48.50*	52.00**	3.50	97.39**	187.19**	33.01	12.72	15.95	12.83	30.85	13.78	123.30	20.36
L8 x T2	50.50	55.17	4.67	90.96	167.31	31.54	13.89	16.05	11.66	33.52**	13.78	122.52	17.88
L8 xT3	50.33	54.17	3.83	91.94	167.72	33.42	15.89*	15.95	12.54	37.22**	13.39	154.49**	23.64**
L8 x T4	50.83	55.00	4.17	76.22	146.76	30.56	14.00	15.46	13.10	34.01**	14.50	130.54	18.22
L8 x T5	49.67*	54.33	4.67	85.89	158.58	31.58	16.67**	16.39	12.97	36.42**	15.33**	154.02**	20.47
L8 x T6	50.83	54.67	3.83	95.37**	175.22	32.67	14.22	17.82**	13.74**	36.77**	15.22**	210.24**	25.89**
L8 x T7	49.83*	53.83	4.00	86.78	156.61	31.06	14.33	16.42	13.07	31.13	14.22	126.58	18.34
L8 x T8	45.83**	49.83**	4.00	89.50	170.28	35.97**	14.17	18.32	13.10	37.07**	13.61	141.40	20.72
L9 x T1	49.67*	53.17**	3.50	96.03**	179.44**	34.34*	14.06	16.43	12.80	30.63	15.06**	108.17	15.87
L9 x T2	44.00**	48.00**	4.00	97.64**	166.92	34.78*	15.75*	15.88	11.97	32.26	16.06**	112.87	17.98
L9 xT3	48.00**	51.50**	3.50	85.54	166.14	35.07**	14.61	17.33*	13.47*	35.76**	13.67	150.50	22.39*
L9 x T4	48.17**	52.83**	4.67	79.00	148.11	32.01	14.61	16.43	13.11	34.66**	14.28	137.37	23.62**
L9 x T5	48.00**	51.50**	3.50	89.47	170.72	31.08	16.11**	17.12	12.13	35.80**	15.78**	158.67**	21.26
L9 x T6	50.67	53.83	3.17*	98.39**	180.14**	31.89	14.56	16.50	12.81	27.47	12.89	109.74	17.66
L9 x T7	50.00*	53.83	3.83	84.83	154.07	34.03	15.67*	16.86	13.48*	32.20	14.00	147.94**	23.28**
L9 x T8	51.67	54.33	2.67**	93.46	162.73	33.35	12.94	16.34	11.95	28.70	15.83**	142.36	24.99**
**Overall mean**	**50.58**	**54.31**	**3.73**	**88.93**	**168.00**	**32.35**	**14.08**	**16.38**	**12.78**	**31.64**	**14.34**	**139.26**	**20.67**
CD at 5 %	0.58	0.66	0.48	4.62	8.28	1.93	1.51	0.85	0.67	1.05	0.52	4.11	1.56
CD at 1 %	0.78	0.87	0.63	6.14	11.01	2.56	2.00	1.13	0.90	1.40	0.69	5.46	2.08

Table 22. Cont.,

	SH%	SPY	100 SW	SL	SG	ST	SVI	STR	HIP	TRP	PA
L1 x T1	81.34	103.54	26.35	0.91**	0.73**	0.37*	3914.16	67.41	1.44**	0.06	5.23**
L1 x T2	85.52	113.12	26.16	0.91**	0.70**	0.33	3456.07	67.69	1.05	0.07**	8.28
L1 xT3	81.72	84.06	24.17	0.82	0.70**	0.40**	3228.28	66.45	0.84	0.05	8.97
L1 x T4	81.98	129.91*	26.93*	0.81	0.67	0.34	2896.82	70.68*	1.73**	0.07**	4.50**
L1 x T5	85.29	138.90**	32.22**	0.89*	0.71**	0.35	3972.89	66.12	1.36**	0.06	6.95**
L1 x T6	83.51	128.56	31.26**	0.87	0.76**	0.36	4410.76*	73.60**	0.97	0.06	8.97
L1 x T7	84.81	145.47**	33.76**	0.93**	0.73**	0.36	3961.42	69.31	1.03	0.06	7.03**
L1 x T8	81.91	99.82	24.75	0.82	0.65	0.32	4935.80**	64.97	0.76	0.06	11.36
L2 x T1	89.08**	155.64**	28.75**	0.92**	0.65	0.38**	5047.74**	71.66**	0.87	0.06	10.25
L2 x T2	87.16**	119.89	26.67	0.86	0.67	0.30	4708.61**	76.39**	0.80	0.07**	9.97
L2 x T3	84.32	96.96	23.90	0.73	0.60	0.32	4193.33	66.53	0.95	0.06	8.09
L2 x T4	87.42**	146.59**	29.85**	0.83	0.76**	0.31	4313.92	72.07**	0.95	0.06	8.29
L2 x T5	86.15	123.77*	27.66**	0.82	0.71**	0.37*	4112.32	70.52**	0.87	0.06	8.42
L2 x T6	87.45**	143.98**	32.08**	0.93**	0.74**	0.39**	4414.34*	65.21	0.97	0.06	7.88
L2 x T7	85.18	135.22**	30.68**	0.90**	0.66	0.33	4827.64**	70.70**	0.95	0.05	8.50
L2 x T8	85.47	107.34	29.37**	0.80	0.64	0.40**	4124.83	66.15	1.17**	0.06	6.88**
L3 x T1	85.63	125.50**	30.39**	0.84	0.70**	0.38**	4110.90	69.03	0.94	0.07**	7.93
L3 x T2	84.58	114.03	26.43	0.84	0.69	0.34	4036.51	65.69	1.11*	0.06	6.30**
L3 xT3	84.67	116.57	29.29**	0.86	0.66	0.39*	4302.94	75.91**	0.93	0.06	7.68
L3 x T4	87.26**	166.39**	31.93**	0.87	0.68	0.40**	4178.84	76.64**	0.99	0.06	7.04**
L3 x T5	84.44	110.25	24.94	0.77	0.54	0.37*	3963.87	69.33	0.97	0.06	8.10
L3 x T6	83.90	127.41**	32.43**	0.85	0.74**	0.40**	4204.16	70.04*	0.87	0.07**	8.33
L3 x T7	85.62	119.45	30.80**	0.90**	0.67	0.39**	4165.20	68.02	1.05	0.06	8.01
L3 x T8	86.56*	143.25**	31.15**	0.83	0.71**	0.38**	4163.38	71.43**	0.99	0.06	9.17
L4 x T1	83.58	102.43	26.48	0.88*	0.66	0.36	3884.87	61.06	1.06	0.06	7.99
L4 x T2	88.93**	112.26	24.72	0.76	0.66	0.28	4110.95	64.12	1.02	0.06	7.55
L4 xT3	89.21**	117.99	23.82	0.82	0.61	0.31	4247.92	65.13	1.05	0.06	7.42
L4 x T4	84.73	131.89**	26.36	0.88*	0.54	0.34	3366.62	68.21	0.85	0.07**	10.17
L4 x T5	82.92	96.05	25.07	0.77	0.61	0.31	4331.86	67.44	1.09	0.06	7.71
L4 x T6	85.23	136.28**	28.37**	0.94**	0.80**	0.36	4580.56**	74.83**	1.04	0.07**	8.64
L4 x T7	82.92	111.54	23.37	0.81	0.62	0.31	4460.72**	64.25	1.11*	0.06	8.07
L4 x T8	83.62	110.45	24.22	0.81	0.58	0.28	4193.99	69.32	0.95	0.07**	7.98
L5 x T1	84.26	101.79	24.48	0.86	0.55	0.33	3422.47	66.59	1.36**	0.06	6.69**
L5 x T2	83.71	99.18	23.66	0.85	0.67	0.31	3081.34	64.32	1.04	0.07**	7.37*
L5 xT3	85.82	108.30	23.73	0.85	0.68	0.37*	3351.46	58.72	1.14**	0.06	6.64**
L5 x T4	85.93	114.09	23.14	0.88*	0.66	0.33	3167.25	63.86	0.88	0.06	8.15

L5 x T5	87.11**	105.23	24.62	0.88*	0.57	0.33	3933.83	66.35	0.85	0.06	8.86
L5 x T6	85.41	144.05**	31.41**	0.98**	0.78	0.40**	4222.37	72.07**	1.24**	0.06	6.92**
L5 x T7	85.01	107.27	26.22	0.91**	0.68	0.37*	4703.23**	65.39	0.93	0.06	8.27
L5 x T8	82.56	88.11	22.90	0.70	0.50	0.30	3106.58	65.04	0.76	0.06	11.21
L6 x T1	84.53	95.24	20.47	0.83	0.68	0.36	3322.13	63.77	0.83	0.07**	9.61
L6 x T2	81.35	74.09	17.42	0.84	0.66	0.31	3408.98	59.59	1.24**	0.06	7.03*
L6 xT3	82.57	97.41	25.96	0.83	0.65	0.32	4412.14*	63.30	1.03	0.07**	6.71**
L6 x T4	86.28	128.11**	22.87	0.93**	0.60	0.37*	4970.58**	70.19**	0.90	0.06	8.38
L6 x T5	84.62	99.85	23.01	0.70	0.58	0.38**	4157.76	72.21**	0.87	0.07**	8.83
L6 x T6	84.94	120.68	30.81**	0.87	0.70**	0.33	3560.15	66.75	1.04	0.06	8.17
L6 x T7	86.29	135.41**	26.46	0.93**	0.66	0.39**	4761.10**	69.18	1.05	0.07**	8.02
L6 x T8	84.33	93.22	20.81	0.78	0.63	0.36	5233.15**	66.07	0.97	0.07**	7.89
L7 x T1	88.86**	174.95**	25.32	0.82	0.63	0.47**	5097.40**	69.36	0.96	0.06	7.98
L7 x T2	87.65**	129.03**	27.48**	0.91**	0.71**	0.38**	5363.60**	69.97*	0.96	0.07**	7.95
L7 xT3	84.66	129.98**	26.20	0.80	0.63	0.33	4893.82**	70.47**	1.34**	0.05	5.93**
L7 x T4	82.83	100.79	24.10	0.75	0.42	0.30	3247.42	68.14	0.83	0.07**	8.87
L7 x T5	85.23	113.59	22.32	0.76	0.54	0.34	5161.28**	72.69**	0.73	0.06	10.86
L7 x T6	87.39**	135.00**	28.62**	0.97**	0.73**	0.37*	5017.22**	72.63**	1.10*	0.05	7.96
L7 x T7	84.97	143.52**	32.40**	0.86	0.75**	0.36	4839.11**	70.49*	0.93	0.05	9.39
L7 x T8	84.53	100.97	23.84	0.80	0.70**	0.29	4084.98	66.23	1.25**	0.05	6.89**
L8 x T1	83.41	102.94	24.24	0.86	0.71**	0.32	2889.13	60.42	1.25**	0.07**	7.04**
L8 x T2	85.43	104.64	22.78	0.86	0.71**	0.36	3948.02	67.34	0.91	0.06	8.53
L8 xT3	84.76	130.85**	26.79	0.86	0.58	0.32	5554.24**	68.37	1.03	0.05	7.61
L8 x T4	86.09	112.32	22.93	0.82	0.65	0.37*	3929.67	67.56	0.89	0.06	8.72
L8 x T5	86.71*	133.54**	23.73	0.83	0.63	0.35	4857.65**	67.15	1.05	0.06	7.77
L8 x T6	87.67**	184.35**	32.81**	0.98**	0.79**	0.45**	5322.72**	76.93**	0.96	0.06	9.38
L8 x T7	85.54	108.24	24.63	0.86	0.70**	0.35	4546.65**	67.41	0.91	0.05	8.10
L8 x T8	85.36	120.68	23.99	0.79	0.64	0.30	4895.72**	72.16**	0.88	0.05	9.69
L9 x T1	85.26	92.29	20.02	0.76	0.55	0.32	3799.13	55.99	1.15**	0.06	5.62**
L9 x T2	84.06	94.89	18.47	0.72	0.57	0.32	3634.44	67.53	1.33**	0.04	5.66**
L9 xT3	84.98	128.11**	26.14	0.86	0.67	0.37*	4987.85**	66.46	1.09	0.06	6.72**
L9 x T4	82.44	113.76	22.80	0.87	0.65	0.39**	4463.73*	70.42**	1.18**	0.06	6.96**
L9 x T5	86.62	137.41	24.42	0.80	0.57	0.31	4775.77**	70.53**	0.87	0.06	8.36
L9 x T6	83.93	92.08	27.00*	0.86	0.67	0.32	3766.29	66.36	1.04	0.06	6.50**
L9 x T7	83.93	124.66**	27.53**	0.88*	0.67	0.33	4631.64**	76.62**	1.08	0.06	7.59*
L9 x T8	82.11	117.37	26.06	0.72	0.56	0.33	3440.79	67.18	0.83	0.05	9.45
Overall mean	**85.02**	**118.70**	**26.21**	**0.84**	**0.66**	**0.35**	**4205.35**	**68.16**	**1.02**	**0.06**	**8.00**
CD at 5 %	1.47	3.84	0.66	0.04	0.03	0.02	185.84	1.39	0.08	0.01	0.40
CD at 1 %	1.96	5.10	0.88	0.06	0.04	0.03	246.85	1.85	0.10	0.01	0.53

Table 23. *sca* of the 72 hybrids across locations

Code No	50 DT	50 DS	ASI	CPH	PH	TL	TBR	CL	CG	NKr/R	NR/C	CW	SHW
L1 x T1	0.69 **	0.81 **	0.12	5.51 **	0.35	-3.69 **	0.53	0.60	-0.17	0.01	-0.87 **	-10.52 **	1.32 *
L1 x T2	-0.68 **	-0.74 **	-0.07	-2.43	-3.54	1.71 *	1.26 **	0.02	0.39	0.84 *	0.36	6.35 **	-1.93 **
L1 xT3	0.84 **	0.26	-0.58 **	5.08 **	-6.96 *	-2.04 **	3.00 **	-0.43	-0.01	-4.24 **	0.13	-30.41 **	-4.18 **
L1 x T4	0.28	-0.13	-0.41 *	4.76 **	22.11 **	0.19	0.56	0.35	0.95 **	0.74	-0.01	1.31	1.99 **
L1 x T5	0.04	0.90 **	0.86 **	6.72 **	10.96 **	-0.56	0.21	1.97 **	-0.29	1.48 **	-0.59 **	24.06 **	3.38 **
L1 x T6	-1.96 **	-1.28 **	0.68 **	-6.21 **	-0.10	4.78 **	-2.16 **	-0.19	-0.41	0.71	0.05	-5.05 **	-0.67
L1 x T7	-0.99 **	-1.51 **	-0.50 **	0.52	-2.48	0.18	-1.73 **	0.60	-0.03	2.28 **	-0.09	22.98 **	1.38 *
L1 x T8	1.78 **	1.68 **	-0.10	-13.96 **	-20.33 **	-0.56	-1.68 **	-2.92 **	-0.42	-1.81 **	1.02 **	-8.74 **	-1.30 *
L2 x T1	0.57 **	0.34	-0.23	-0.85	6.17	1.12	1.16 **	-0.00	0.17	4.25 **	0.69 **	28.74 **	0.33
L2 x T2	-0.31	-0.56 *	-0.25	12.23 **	16.14 **	1.34	2.09 **	0.38	0.37	-0.25	0.11	3.71 *	0.51
L2 x T3	2.21 **	2.94 **	0.73 **	-18.44 **	-31.51 **	-12.25 **	-5.12 **	-1.18 **	-0.89 **	-0.79	-0.47 *	-26.46 **	-1.28 *
L2 x T4	0.49 *	0.05	-0.44 *	5.51 **	-3.11	-2.12 **	-0.39	0.66 *	-0.59 *	0.32	0.06	10.32 **	0.04
L2 x T5	1.75 **	1.43 **	-0.33	2.11	-1.71	2.79 **	1.92 **	1.01 **	1.07 **	-0.55	-0.16	-2.98	0.66
L2 x T6	-0.25	-0.76 **	-0.51 **	-4.46 **	-0.08	1.41 *	0.58	-0.18	0.38	1.71 **	-0.32	-1.81	-1.00
L2 x T7	-1.12 **	-0.65 **	0.47 **	12.00 **	22.51 **	2.49 **	-0.68	-0.52	-0.15	-0.60	0.49 *	1.91	2.41 **
L2 x T8	-3.34 **	-2.80 **	0.55 **	-8.11 **	-8.42 **	5.24 **	0.44	-0.18	-0.36	-4.10 **	-0.39	-13.43 **	-1.67 **
L3 x T1	1.40 **	1.04 **	-0.36 *	4.39 **	-3.87	-5.15 **	-1.76 **	0.00	-0.23	-0.35	-0.19	-1.08	-0.38
L3 x T2	0.53 *	0.49 *	-0.04	-3.52 *	-10.15 **	-2.44 **	0.51	-0.30	-0.48	1.03 *	-0.40 *	-0.19	1.44 *
L3 xT3	-0.29	-1.02 **	-0.72 **	17.33 **	25.00 **	3.03 **	0.63	0.03	-0.04	-1.55 **	0.05	-4.78 **	-0.23
L3 x T4	0.16	0.26	0.11	-6.85 **	-9.26 **	-0.61	-0.91 *	0.32	0.77 **	2.32 **	0.18	32.24 **	2.81 **
L3 x T5	-0.92 **	-0.53 *	0.38 *	0.78	6.92 *	0.90	1.61 **	-1.13 **	-0.13	-0.46	0.32	-17.55 **	-1.43 *
L3 x T6	1.75 **	1.95 **	0.20	-7.85 **	-16.13 **	-3.08 **	-2.36 **	0.13	-0.26	-1.98 **	0.23	-15.35 **	1.01
L3 x T7	-0.95 **	-1.61 **	-0.65 **	-8.22 **	-11.81 **	4.50 **	1.02 *	0.56	0.12	-1.89 **	-0.47 *	-19.02 **	-3.78 **
L3 x T8	-1.67 **	-0.59 *	1.09 **	3.94 *	19.30 **	2.85 **	1.25 **	0.38	0.26	2.89 **	0.28	25.73 **	0.56
L4 x T1	-4.41 **	-3.07 **	1.35 **	-5.37 **	-4.35	1.33	0.21	0.26	0.01	-3.01 **	-0.98 **	-9.93 **	0.64
L4 x T2	-3.45 **	-3.45 **	-0.00	-11.73 **	-13.77 **	-1.53 *	-1.67 **	-0.74 *	-0.58 *	0.32	-0.29	6.00 **	-2.14 **
L4 xT3	0.73 **	1.05 **	0.31	-10.26 **	-13.14 **	-1.90 **	0.23	-1.01 **	-1.02 **	2.50 **	0.59 **	4.98 **	-3.43 **
L4 x T4	1.51 **	0.99 **	-0.52 **	12.35 **	10.29 **	-0.66	0.41	0.54	0.61 *	-1.73 **	1.17 **	11.31 **	1.50 *
L4 x T5	2.60 **	1.03 **	-1.57 **	0.22	1.76	4.68 **	1.68 **	-0.89 **	0.32	-4.18 **	-0.74 **	-16.81 **	0.32
L4 x T6	3.77 **	3.68 **	-0.09	5.08 **	11.35 **	0.11	0.85 *	0.05	0.75 **	2.71 **	0.12	7.60 **	1.88 **
L4 x T7	-0.43 *	-0.55 *	-0.11	8.25 **	5.28	-0.96	0.25	1.16 **	0.03	1.87 **	0.15	-10.84 **	-0.89
L4 x T8	-0.32	0.31	0.63 **	1.45	2.59	-1.07	-1.97 **	0.64 *	-0.11	1.52 **	-0.01	7.70 **	2.12 **
L5 x T1	2.02 **	1.46 **	-0.56 **	-11.74 **	-10.47 **	-2.26 **	0.75	0.53	0.16	-2.31 **	0.27	-4.58 **	0.68
L5 x T2	0.49 *	0.57 *	0.08	1.40	14.70 **	-0.51	-0.42	0.88 **	0.45	0.13	-0.32	5.30 **	2.64 **
L5 xT3	-3.66 **	-3.77 **	-0.10	-4.96 **	-8.24 *	2.91 **	-0.46	0.09	-0.09	1.36 **	0.56 **	5.58 **	-0.75
L5 x T4	-1.55 **	-0.82 **	0.73 **	0.94	4.19	5.66 **	1.53 **	-0.02	-0.82 **	1.53 **	-0.03	-3.88 *	-1.84 **
L5 x T5	-1.63 **	-0.45	1.18 **	7.12 **	11.80 **	2.09 **	-0.96 *	-0.51	0.17	-1.73 **	0.28	-5.15 **	-3.15 **
L5 x T6	-2.96 **	-3.14 **	-0.17	7.52 **	7.22 *	1.82 **	1.07 **	0.65 *	0.42	1.21 **	0.64 **	23.23 **	3.79 **

L5 x T7	2.67 **	2.98 **	0.30	-8.01 **	-11.24 **	-3.03 **	-0.84 *	-1.00 **	-0.62 *	-0.94 *	-0.05	-9.46 **	-1.18
L5 x T8	4.62 **	3.16 **	-1.46 **	7.72 **	-7.97 *	-6.67 **	-0.66	-0.62	0.33	0.75	-1.35 **	-11.03 **	-0.20
L6 x T1	2.23 **	2.44 **	0.21	-1.08	-5.03	0.05	-1.18 **	-0.28	-0.02	1.65 **	-0.44 *	-9.77 **	-0.95
L6 x T2	1.03 **	-0.12	-1.14 **	-6.17 **	-2.36	3.10 **	-1.91 **	0.71 *	0.12	-1.45 **	0.24	-18.85 **	0.58
L6 xT3	-0.79 **	-0.29	0.50 **	-9.75 **	-11.10 **	4.14 **	2.13 **	0.66 *	0.47	-3.65 **	-0.55 **	-0.23	1.33 *
L6 x T4	-1.01 **	-1.50 **	-0.50 **	-3.85 *	-4.12	-1.46 *	-0.28	0.21	-0.45	3.24 **	0.75 **	14.52 **	-0.46
L6 x T5	-0.58 **	-0.97 **	-0.39 *	0.34	-5.73	-3.46 **	-1.68 **	-0.17	-0.14	0.06	-0.98 **	-4.77 **	-0.39
L6 x T6	-0.75 **	-0.49 *	0.26	12.59 **	11.02 **	-3.89 **	0.89 *	-0.62	-0.49	-2.85 **	-0.23	-0.66	0.28
L6 x T7	-0.12	0.29	0.41 *	6.12 **	16.64 **	2.22 **	0.76	-0.22	0.59 *	3.16 **	0.80 **	24.29 **	1.44 *
L6 x T8	-0.01	0.64 **	0.65 **	1.80	0.67	-0.71	1.27 **	-0.29	-0.09	-0.16	0.41 *	-4.55 **	-1.83 **
L7 x T1	-2.10 **	-1.48 **	0.62 **	-0.62	-4.19	9.04 **	3.09 **	0.27	-0.31	5.24 **	2.02 **	49.76 **	1.85 **
L7 x T2	2.53 **	3.13 **	0.60 **	-1.01	-8.14 *	-2.18 **	0.55	0.03	0.71 **	-0.54	-0.24	12.24 **	-0.29
L7 xT3	0.88 **	0.63 **	-0.25	13.76 **	28.46 **	1.75 *	-1.57 **	0.81 *	0.68 **	0.01	0.75 **	11.17 **	3.14 **
L7 x T4	-0.18	-0.09	0.09	5.08 **	3.71	0.61	-1.09 **	-1.32 **	-0.10	-4.82 **	-1.19 **	-36.93 **	-1.60 **
L7 x T5	0.58 **	-0.05	-0.63 **	-21.87 **	-33.99 **	-8.41 **	-3.82 **	-1.23 **	-1.03 **	0.69	0.18	-14.23 **	-0.61
L7 x T6	-0.58 **	-0.24	0.34 *	1.27	-9.00 **	0.28	0.66	-0.34	-0.33	0.99 *	-0.44 *	-13.01 **	-3.43 **
L7 x T7	-0.29	-0.29	-0.01	2.11	19.14 **	-1.93 **	-0.08	0.83 *	0.19	-0.55	-0.74 **	11.18 **	3.19 **
L7 x T8	-0.84 **	-1.61 **	-0.77 **	1.28	4.00	0.85	2.26 **	0.95 **	0.20	-1.02 *	-0.34	-20.19 **	-2.25 **
L8 x T1	-1.17 **	-1.98 **	-0.82 **	6.20 **	14.49 **	-0.46	-1.91 **	-0.90 **	0.13	-3.85 **	-0.66 **	-19.72 **	0.60
L8 x T2	2.80 **	3.30 **	0.50 **	2.92	3.68	-0.93	-0.98 *	-0.37	-0.73 **	-0.63	-0.64 **	-8.42 **	-0.36
L8 xT3	0.82 **	0.80 **	-0.02	7.47 **	9.44 **	1.78 *	1.36 **	-0.14	-0.09	2.69 **	-0.43 *	16.10 **	3.42 **
L8 x T4	1.10 **	1.08 **	-0.02	-9.71 **	-12.67 **	-1.11	-0.07	-0.83 *	-0.27	-2.34 **	-0.12	-23.83 **	-3.82 **
L8 x T5	-0.48 *	0.11	0.60 **	1.14	-1.16	1.66 *	0.96 *	0.14	0.35	1.43 **	0.86 **	10.42 **	0.31
L8 x T6	0.19	-0.07	-0.26	-4.84 **	-4.69	0.10	0.21	0.94 **	0.36	2.18 **	1.38 **	46.81 **	3.28 **
L8 x T7	0.15	0.04	-0.11	-4.77 **	-17.84 **	-2.80 **	0.13	-0.89 **	-0.35	-3.42 **	0.30	-27.16 **	-3.67 **
L8 x T8	-3.40 **	-3.27 **	0.13	1.60	8.76 **	1.77 *	0.31	2.04 **	0.59 *	3.93 **	-0.69 **	5.80 **	0.25
L9 x T1	0.78 **	0.44	-0.34	3.55 *	6.91 *	0.03	-0.88 *	-0.49	0.26	-1.64 **	0.16	-22.91 **	-4.08 **
L9 x T2	-2.93 **	-2.62 **	0.31	8.30 **	3.46	1.45 *	0.58	-0.61	-0.26	0.54	1.17 **	-6.15 **	-0.45
L9 xT3	-0.75 **	-0.62 **	0.13	-0.23	8.03 *	2.59 **	-0.22	1.18 **	0.99 **	3.68 **	-0.61 **	24.05 **	1.98 **
L9 x T4	-0.80 **	0.16	0.96 **	-8.22 **	-11.14 **	-0.50	0.24	0.08	-0.10	0.74	-0.81 **	-5.07 **	1.38 *
L9 x T5	-1.38 **	-1.47 **	-0.10	3.44 *	11.16 **	0.32	0.10	0.81 *	-0.33	3.26 **	0.83 **	27.01 **	0.91
L9 x T6	0.79 **	0.34	-0.45 *	-3.11	0.40	-1.52 *	0.24	-0.44	-0.41	-4.68 **	-1.42 **	-41.75 **	-5.14 **
L9 x T7	1.09 **	1.29 **	0.20	-8.00 **	-20.21 **	-0.67	1.16 **	-0.52	0.23	0.09	-0.39	6.13 **	1.09
L9 x T8	3.20 **	2.48 **	-0.72 **	4.28 **	1.39	-1.70 *	-1.22 **	0.00	-0.39	-1.99 **	1.06 **	18.69 **	4.32 **

Table 23. Cont.,

	SH%	SPY	100 SW	SL	SG	ST	SVI	STR	HIP	TRP	PA
L1 x T1	-2.01 **	-12.07 **	-0.81 **	0.03	0.03 *	0.00	329.38 **	2.26 **	0.22 **	-0.00	-2.07 **
L1 x T2	1.90 **	7.88 **	0.41	0.04 *	-0.02	0.01	-157.67 *	0.62	-0.12 **	0.00	0.94 **
L1 xT3	-1.27 *	-26.64 **	-3.38 **	-0.03	0.01	0.05 **	-765.84 **	-0.49	-0.33 **	-0.01 **	1.96 **
L1 x T4	-1.26 *	-1.09	-0.71 **	-0.06 **	-0.00	-0.01	-582.06 **	0.81	0.58 **	0.01 **	-2.73 **
L1 x T5	1.59 **	22.82 **	4.89 **	0.06 **	0.05 **	0.00	-31.81	-3.14 **	0.27 **	0.00	-1.19 **
L1 x T6	-0.23	-4.61 **	-1.26 **	-0.07 **	-0.04 **	-0.02 *	380.36 **	2.55 **	-0.19 **	-0.01 *	1.18 **
L1 x T7	1.65 **	21.38 **	3.34 **	0.02	0.00	-0.00	-224.34 **	0.15	-0.11 **	0.00	-0.79 **
L1 x T8	-0.38	-7.66 **	-2.47 **	0.01	-0.02 *	-0.02	1051.99 **	-2.77 **	-0.32 **	0.00	2.70 **
L2 x T1	2.46 **	28.52 **	1.17 **	0.06 **	-0.02 *	0.02	842.14 **	4.88 **	-0.15 **	-0.00	2.13 **
L2 x T2	0.28	3.12 *	0.50 *	0.02	-0.02 *	-0.03 **	474.06 **	7.69 **	-0.17 **	0.00	1.81 **
L2 x T3	-1.94 **	-25.26 **	-4.06 **	-0.10 **	-0.07 **	-0.03 *	-421.61 **	-2.03 **	-0.02	0.00	0.25 *
L2 x T4	0.92	10.21 **	1.78 **	-0.02	0.11 **	-0.04 **	214.22 **	0.57	0.01	-0.00	-0.19
L2 x T5	-0.82	-3.83 *	-0.08	0.01	0.08 **	0.03 *	-513.20 **	-0.37	-0.01	0.00	-0.54 **
L2 x T6	0.45	-0.71	-0.87 **	0.01	-0.03 *	0.02	-236.89 **	-7.47 **	0.03	-0.00	-0.73 **
L2 x T7	-1.25 *	-0.40	-0.16	0.01	-0.04 **	-0.03 *	21.07	-0.08	0.02	-0.01 *	-0.14
L2 x T8	-0.09	-11.66 **	1.73 **	0.01	-0.00	0.07 **	-379.80 **	-3.21 **	0.29 **	0.01 *	-2.60 **
L3 x T1	0.22	-0.80	1.76 **	-0.02	0.03 **	-0.02	232.41 **	1.40 **	-0.12 **	0.01 *	0.52 **
L3 x T2	-1.12 *	-1.92	-0.79 **	0.00	-0.00	-0.02	129.08	-3.87 **	0.10 **	-0.01 **	-1.13 **
L3 xT3	-0.39	-4.84 **	0.27	0.04 *	-0.00	0.01	15.13	6.50 **	-0.07 *	0.00	0.56 **
L3 x T4	1.94 **	30.82 **	2.81 **	0.02	0.04 **	0.02	406.25 **	4.29 **	0.01	-0.00	-0.71 **
L3 x T5	-1.33 *	-16.52 **	-3.85 **	-0.03	-0.08 **	-0.01	-334.53 **	-2.42 **	0.04	-0.00	-0.15
L3 x T6	-1.91 **	-16.46 **	-1.56 **	-0.07 **	-0.02	-0.01	-119.95	-3.50 **	-0.12 **	0.01 **	0.43 **
L3 x T7	0.39	-15.35 **	-1.09 **	0.01	-0.03 **	-0.00	-314.25 **	-3.62 **	0.08 *	0.00	0.08
L3 x T8	2.20 **	25.07 **	2.46 **	0.04 *	0.07 **	0.02 *	-14.14	1.22 *	0.08 *	-0.00	0.41 **
L4 x T1	-1.65 **	-10.88 **	2.22 **	0.04 *	0.03 *	0.02 *	-0.07	-2.60 **	-0.04	-0.01 *	0.21
L4 x T2	3.43 **	9.30 **	1.88 **	-0.07 **	0.01	-0.01	197.05 **	-1.47 **	-0.04	0.00	-0.27 *
L4 xT3	4.33 **	9.58 **	-0.83 **	0.00	-0.01	-0.00	-46.36	-0.32	-0.00	-0.00	-0.07
L4 x T4	-0.39	9.32 **	1.61 **	0.04 *	-0.07 **	0.01	-412.43 **	-0.18	-0.17 **	0.00	2.04 **
L4 x T5	-2.66 **	-17.74 **	0.65 **	-0.02	0.02	-0.01	26.99	-0.34	0.13 **	-0.01 **	-0.90 **
L4 x T6	-0.39	5.41 **	-1.26 **	0.03	0.08 **	0.02	249.99 **	5.26 **	0.01	0.01	0.37 **
L4 x T7	-2.12 **	-10.26 **	-4.16 **	-0.07 **	-0.04 **	-0.01	-25.19	-3.42 **	0.10 **	-0.00	-0.23
L4 x T8	-0.55	5.27 **	-0.11	0.04 *	-0.02 *	-0.02 *	10.02	3.07 **	0.00	0.01 **	-1.16 **
L5 x T1	-0.80	-5.15 **	0.50 *	-0.01	-0.09 **	-0.03 **	61.15	4.43 **	0.26 **	-0.00	-0.91 **
L5 x T2	-1.63 **	2.58	1.09 **	-0.01	0.02	-0.01	-308.94 **	0.23	-0.02	0.01 **	-0.26 *
L5 xT3	1.11 *	6.25 **	-0.64 **	0.00	0.06 **	0.03 **	-419.20 **	-5.22 **	0.09 **	0.00	-0.67 **
L5 x T4	0.98	-2.12	-1.33 **	0.02	0.06 **	-0.01	-88.17	-3.02 **	-0.15 **	-0.00	0.19

L5 x T5	1.70 **	-2.20	0.48 *	0.06 **	-0.02	-0.01	152.59 *	0.07	-0.12 **	-0.01	0.42 **
L5 x T6	-0.05	19.53 **	2.06 **	0.05 **	0.06 **	0.03 **	415.42 **	4.01 **	0.20 **	0.00	-1.17 **
L5 x T7	0.14	-8.18 **	-1.02 **	0.00	0.02	0.02 *	740.93 **	-0.78	-0.07 *	0.00	0.15
L5 x T8	-1.45 *	-10.72 **	-1.14 **	-0.10 **	-0.10 **	-0.02	-553.78 **	0.29	-0.20 **	0.00	2.26 **
L6 x T1	0.07	-8.71 **	-1.96 **	-0.02	0.05 **	-0.01	-643.87 **	0.51	-0.24 **	-0.00	1.95 **
L6 x T2	-3.37 **	-19.50 **	-3.60 **	0.01	0.00	-0.02	-585.98 **	-5.60 **	0.22 **	-0.01 **	-0.67 **
L6 xT3	-1.52 **	-1.64	3.13 **	0.01	0.02	-0.03 **	36.80	-1.73 **	0.01	0.01 *	-0.67 **
L6 x T4	1.94 **	14.90 **	-0.05	0.08 **	-0.02	0.02 *	1110.47 **	2.22 **	-0.09 **	-0.01 **	0.36 **
L6 x T5	-0.18	-4.57 **	0.41	-0.10 **	-0.01	0.03 **	-228.16 **	4.84 **	-0.06 *	0.00	0.33 **
L6 x T6	0.11	-0.83	3.01 **	-0.04 *	-0.03 **	-0.05 **	-851.48 **	-2.41 **	0.04	-0.00	0.01
L6 x T7	2.02 **	22.96 **	0.76 **	0.05 **	-0.01	0.04 **	194.12 **	1.91 **	0.07 *	0.01 **	-0.16
L6 x T8	0.93	-2.60	-1.70 **	0.01	0.02	0.02 *	968.11 **	0.24	0.05	0.01 *	-1.13 **
L7 x T1	3.01 **	48.03 **	0.07	-0.02	0.00	0.10 **	646.54 **	2.49 **	-0.13 **	-0.00	0.16
L7 x T2	1.53 **	12.45 **	3.65 **	0.08 **	0.06 **	0.04 **	883.79 **	1.17 *	-0.09 **	0.01 **	0.10
L7 xT3	-0.83	7.95 **	0.57 *	-0.01	0.01	-0.03 **	33.62	1.82 **	0.30 **	-0.01 *	-1.60 **
L7 x T4	-2.91 **	-35.41 **	-1.64 **	-0.09 **	-0.19 **	-0.06 **	1097.54 **	-3.44 **	-0.18 **	0.01 **	0.70 **
L7 x T5	-0.97	-13.81 **	-3.09 **	-0.03	-0.05 **	-0.01	290.49 **	1.71 **	-0.23 **	0.00	2.21 **
L7 x T6	1.16 *	-9.48 **	-1.99 **	0.06 **	-0.00	-0.01	120.74	-0.14	0.09 **	-0.01 **	-0.35 **
L7 x T7	-0.70	8.10 **	3.89 **	-0.02	0.08 **	0.00	-212.72 **	-0.39	-0.06 *	-0.00	1.06 **
L7 x T8	-0.27	-17.83 **	-1.47 **	0.03	0.10 **	-0.04 **	-664.91 **	-3.23 **	0.31 **	-0.01 *	-2.27 **
L8 x T1	-2.30 **	-20.21 **	0.04	-0.00	0.04 **	-0.05 **	1341.61 **	-4.87 **	0.19 **	0.01 **	-0.90 **
L8 x T2	-0.55	-8.15 **	-0.01	0.01	0.02	0.03 **	-311.66 **	0.12	-0.11 **	0.00	0.56 **
L8 xT3	-0.58	12.60 **	2.20 **	0.02	-0.08 **	-0.03 **	914.17 **	1.30 *	0.03	-0.00	-0.05
L8 x T4	0.49	-20.08 **	-1.75 **	-0.05 **	0.01	0.02 *	-195.16 **	-2.44 **	-0.10 **	-0.00	0.43 **
L8 x T5	0.65	9.93 **	-0.63 **	0.01	0.00	0.00	206.99 **	-2.25 **	0.12 **	0.00	-1.01 **
L8 x T6	1.58 **	43.64 **	3.25 **	0.05 **	0.02 *	0.07 **	646.36 **	5.74 **	-0.03	0.00	0.94 **
L8 x T7	0.02	-23.39 **	-2.82 **	-0.04 *	-0.00	-0.01	-285.06 **	-1.88 **	-0.06	-0.01 *	-0.35 **
L8 x T8	0.70	5.66 **	-0.28	-0.00	-0.01	-0.03 **	365.96 **	4.29 **	-0.03	-0.01 **	0.40 **
L9 x T1	1.01	-18.72 **	-2.99 **	-0.06 **	-0.05 **	-0.04 **	-126.08	-8.51 **	0.01	0.01 *	-1.08 **
L9 x T2	-0.46	-5.77 **	-3.13 **	-0.08 **	-0.06 **	0.01	-319.73 **	1.10 *	0.23 **	-0.01 **	-1.07 **
L9 xT3	1.09	21.99 **	2.73 **	0.08 **	0.07 **	0.04 **	653.30 **	0.17	0.00	0.01 *	0.31 *
L9 x T4	-1.71 **	-6.53 **	-0.70 **	0.06 **	0.06 **	0.05 **	644.42 **	1.19 *	0.10 **	-0.00	-0.09
L9 x T5	2.01 **	25.91 **	1.24 **	0.03	0.01	-0.02 *	430.63 **	1.91 **	-0.15 **	0.00	0.83 **
L9 x T6	-0.71	-36.50 **	-1.38 **	-0.02	-0.03 *	-0.04 **	-604.54 **	-4.05 **	-0.04	0.00	-0.68 **
L9 x T7	-0.14	5.15 **	1.25 **	0.03	0.02 *	-0.01	105.45	8.10 **	0.03	0.01 *	0.38 **
L9 x T8	-1.08	14.47 **	2.99 **	-0.03	-0.02	0.01	-783.45 **	0.09	-0.18 **	-0.01	1.40 **
